# Multipliers on bi-parameter Haar system Hardy spaces

**DOI:** 10.1007/s00208-024-02887-9

**Published:** 2024-05-25

**Authors:** R. Lechner, P. Motakis, P. F. X. Müller, Th. Schlumprecht

**Affiliations:** 1 Institute of Analysis, Johannes Kepler University Linz, Altenberger Strasse 69, 4040 Linz, Austria; 2 Department of Mathematics and Statistics, York University, 4700 Keele Street, Toronto, ON M3J 1P3 Canada; 3 Department of Mathematics, Texas A &M University, College Station, TX 77843-3368 USA; 4 Faculty of Electrical Engineering, Czech Technical University in Prague, Technika 2, 16627 Praha 6, Czech Republic

**Keywords:** 46B25, 47A68, 30H10, 60G46

## Abstract

Let $(h_I)$ denote the standard Haar system on [0, 1], indexed by $I\in \mathcal {D}$, the set of dyadic intervals and $h_I\otimes h_J$ denote the tensor product $(s,t)\mapsto h_I(s) h_J(t)$, $I,J\in \mathcal {D}$. We consider a class of two-parameter function spaces which are completions of the linear span $\mathcal {V}(\delta ^2)$ of $h_I\otimes h_J$, $I,J\in \mathcal {D}$. This class contains all the spaces of the form *X*(*Y*), where *X* and *Y* are either the Lebesgue spaces $L^p[0,1]$ or the Hardy spaces $H^p[0,1]$, $1\le p < \infty$. We say that $D:X(Y)\rightarrow X(Y)$ is a Haar multiplier if $D(h_I\otimes h_J) = d_{I,J} h_I\otimes h_J$, where $d_{I,J}\in \mathbb {R}$, and ask which more elementary operators factor through *D*. A decisive role is played by the *Capon projection* $\mathcal {C}:\mathcal {V}(\delta ^2)\rightarrow \mathcal {V}(\delta ^2)$ given by $\mathcal {C} h_I\otimes h_J = h_I\otimes h_J$ if $|I|\le |J|$, and $\mathcal {C} h_I\otimes h_J = 0$ if $|I| > |J|$, as our main result highlights: Given any bounded Haar multiplier $D:X(Y)\rightarrow X(Y)$, there exist $\lambda ,\mu \in \mathbb {R}$ such that

$$\begin{aligned} \lambda \mathcal {C} + \mu ({{\,\textrm{Id}\,}}-\mathcal {C})\text { approximately 1-projectionally factors through }D, \end{aligned}$$

i.e., for all $\eta > 0$, there exist bounded operators *A*, *B* so that *AB* is the identity operator ${{\,\textrm{Id}\,}}$, $\Vert A\Vert \cdot \Vert B\Vert = 1$ and $\Vert \lambda \mathcal {C} + \mu ({{\,\textrm{Id}\,}}-\mathcal {C}) - ADB\Vert < \eta$. Additionally, if $\mathcal {C}$ is unbounded on *X*(*Y*), then $\lambda = \mu$ and then ${{\,\textrm{Id}\,}}$ either factors through *D* or ${{\,\textrm{Id}\,}}-D$.

## Introduction

Let us consider a bounded linear operator $T:E \rightarrow E$ on a Banach space *E*, with Schauder basis $(x_n)$ and biorthogonal functionals $(x_n^*)$. All information carried by *T* is then encoded in the matrix,

$$\begin{aligned} ( \langle x_m^*, Tx_n \rangle )_{ m,n =1}^\infty . \end{aligned}$$

Indeed for $f\in E$ with basis expansion $f = \sum _{n =1}^\infty \langle x_n^*, f \rangle x_n$ we have

$$\begin{aligned} Tf = \sum _{n =1}^\infty \sum _{m=1}^\infty \langle x_m^* , Tx_n \rangle \langle x_n^* , f \rangle x_m. \end{aligned}$$

In the special case, where $\langle x_m^*, Tx_n \rangle = 0$ for $m \ne n$, the operator *T* acts directly as a multiplier on the basis $(x_n)$, and

$$\begin{aligned} Tf = \sum _{n =1}^\infty \langle x_n^*, Tx_n \rangle \langle x_n^*, f \rangle x_n . \end{aligned}$$

Accordingly, problems arising in operator theory are investigated alongside suitably chosen Schauder bases and biorthogonal systems in Banach spaces. Thus the Haar system $(h_I)$ and its tensor product arises with the factorization of operators on Lebesgue spaces, Hardy spaces, or mixed norm spaces like $L^p(L^q)$.

Expressing a given operator problem in terms of Haar functions and exploiting their relative simplicity often leads straight to its combinatorial core. In this regard the books Lindenstrauss–Tzafriri [43], Bourgain [5], Hytönen, van Neerven, Veraar and Weis [25, 26] and [50, 52] point out the following notable examples:

Johnson’s [27] factorization of operators on $$L^p $$ through $$\ell ^p$$, the work of Enflo–Maurey [44] and Enflo–Starbird [18] on complemented subspaces of $$L^p$$, Capon’s [13] proof that the space $$L^p(L^q)$$ is primary, the construction by Bourgain, Rosenthal and Schechtman [6] of uncountably many complemented subspaces of $$L^p$$, the subspace stability theorem for $$L^p$$, and for rearrangement invariant function spaces by Johnson, Maurey, Schechtman and Tzafriri [28], Kalton’s $$L^1$$-embeddings [30, 31], the construction of resolving operators in Jones’s [29] work on the uniform approximation property, Pisier’s [56, 57] characterization of martingale cotype and operator valued martingale transforms in the work of Girardi and Weis [22].

Our recent work [39], on $L^1(L^p)$ is typical of this line of research. Establishing that $L^1(L^p)$ is primary amounts to factoring the identity on $L^1(L^p)$ through *T* or ${{\,\textrm{Id}\,}}-T$, for any *T* on $L^1(L^p)$. We reduced the general, functional analytic task to concrete, probabilistic and combinatorial problems, and solved the latter in [39]. The Local Theory of Banach spaces provided a framework and tools, see Milman and Schechtman [47]. Related quantitative, finite dimensional factorization problems arose in the work of Bourgain and Tzafriri [7, 8] on restricted invertibility of operators.

The initial motivation for the present paper was the open problem of whether $L^p(L^1)$, $1< p < \infty$ and the related $H^1(L^1 )$ and $L^1(H^1 )$ are primary spaces. Our main results establish the required factorization theorems in the special case of Haar multipliers on the bi-parameter Haar system. We thereby obtain a first step towards proving that these spaces are primary. Our approach works in a unified way and includes also the spaces $L^1(L^p)$, $1< p < \infty$ considered in our previous work [39]. Even more, our factorization results include the large class of bi-parameter Haar system Hardy spaces (see Definition 2.8 and Theorem 2.14 for the factorization result).

### Concepts, methods and techniques

In the preceding work [39] we proved that $L^1(L^p)$ is primary. We described there the problem’s origin in Lindenstrauss’s [41] famous research program set forth around 1970, its history and its connection to the indecomposable Banach spaces constructed by Gowers and Maurey [23].

We now discuss ideas, methods and techniques pertaining to factorization of operators on classical Banach spaces. Our review covers the main steps of the development. It begins with the pioneering work of Enflo–Maurey and gradually builds the stage on which current research is taking place.

#### The Enflo–Maurey theorem [44]

For $1\le p < \infty$, Enflo via Maurey [44] proved that $L^p$ is primary. Given a linear operator $T:L^p \rightarrow L^p$ they proved that there exists $\alpha \in \mathbb {R}$ such that for any $\varepsilon > 0$ there exist bounded operators $A:L^p \rightarrow L^p$ and $B:L^p \rightarrow L^p$ such that

1.1 $$\begin{aligned} AB = {{\,\textrm{Id}\,}}, \qquad \Vert A\Vert \cdot \Vert B\Vert \le C_0 , \end{aligned}$$

and

1.2 $$\begin{aligned} \Vert ATB -\alpha {{\,\textrm{Id}\,}}\Vert < \varepsilon , \end{aligned}$$

where $C_0 < \infty$ is a universal constant independent of $p,T,\alpha ,\varepsilon$. We then say that *T* is an approximate projectional factor of $\alpha {{\,\textrm{Id}\,}}$, or equivalently that $\alpha {{\,\textrm{Id}\,}}$ approximately projectionally factors through *T*. Separately, we say that *A*, *B* are exterior projectional factors of $\alpha {{\,\textrm{Id}\,}}$. (We refer to Definition 2.2.)

**Step 1** The first step of the Enflo–Maurey method exhibits a sequence of scalars $(\alpha _I: I \in \mathcal {D})$ such that for $\varepsilon > 0$ there exists a block basis of the Haar system $(\tilde{h}_I)$ such that

$$\begin{aligned} A_1:f \mapsto \sum _{I\in \mathcal {D}} \langle \tilde{h}_I, f\rangle h_I/|I|, \quad \text {and}\quad B_1:f \mapsto \sum _{I\in \mathcal {D}} \langle h_I, f \rangle \tilde{h}_I /|I|, \end{aligned}$$

are contractions on $L^p$ such that $A_1B_1 = {{\,\textrm{Id}\,}}$, and $S_1 = A_1TB_1$ is a small perturbation of a Haar multiplier satisfying

$$\begin{aligned} \bigl | \langle S_1 h_I, h_I \rangle - \alpha _I |I| \bigr |< \varepsilon |I|^ 5 , \text { if } I\in \mathcal {D}, \quad \text {and}\quad | \langle S_1 h_I, h_J \rangle | < \varepsilon ( |I| \cdot |J|)^5, \text { if }I \ne J\in \mathcal {D}. \end{aligned}$$

The block basis $(\tilde{h}_I)$ constructed by Enflo–Maurey is what we call “a faithful Haar system” (see Definition 2.16). This means that the orthogonal projection

$$\begin{aligned} P(f) = \sum _{I\in \mathcal {D}} \langle \tilde{h}_I, f \rangle \tilde{h}_I/|I|, \end{aligned}$$

coincides with the conditional expectation, ${{\,\mathrm{\mathbb {E}}\,}}( f | \mathcal {F} )$, where $\mathcal {F}$ is the $\sigma$-algebra generated by the sub-system $(\tilde{h}_I: I \in \mathcal {D} )$, and $f \in \langle \{ h_I: I \in \mathcal {D} \} \rangle$, where $\langle V \rangle$ denotes the linear span of a set of vectors *V*.

**Step 2** The Enflo–Maurey proof utilizes Liapunov’s convexity theorem to pass to Haar multipliers with stable entries. It yields a single scalar $\alpha$ and a block basis of the Haar basis $(\tilde{k}_I)$ (in fact a faithful Haar system) such that

$$\begin{aligned} A_2:f&\mapsto \sum _{I\in \mathcal {D}} \langle \tilde{k}_I, f \rangle h_I/|I|,\\ B_2:f&\mapsto \sum _{I\in \mathcal {D}} \langle h_I, f \rangle \tilde{k}_I/|I|, \end{aligned}$$

are contractions on $L^p$ for which $A_2B_2 = {{\,\textrm{Id}\,}}$ and $S_2 = A_2S_1B_2$ is a small perturbation of $\alpha {{\,\textrm{Id}\,}}$ on $L^p$ satisfying

$$\begin{aligned}&\bigl | \langle S_2 h_I, h_I \rangle - \alpha |I| \bigr |< \varepsilon |I|^ 5, \quad I \in \mathcal {D},\\&| \langle S_2 h_I, h_J \rangle | < \varepsilon (|I|\cdot |J|)^5, \quad I \ne J \in \mathcal {D} . \end{aligned}$$

#### Capon’s theorem on Banach spaces with symmetric bases [14]

Capon [14] proved that for any Banach space *E* with a symmetric basis $(e_i)$, the Bochner Lebesgue space $L^p(E)$ is primary for $1\le p < \infty$. For every bounded linear operator $T:L^p(E)\rightarrow L^p(E)$, Capon [14] determines $\alpha \in \mathbb {R}$ such that *T* is an approximate factor of $\alpha {{\,\textrm{Id}\,}}$. The norms of the exterior projectional factors obtained by Capon depend only on the symmetry constant of the basis $(e_i)$ in *E*. In the course of proving this result, Capon [14] was lead to introduce the following property: *E* together with the symmetric basis $(e_i)$ satisfies condition $\mathcal {P}_p$ if there exists $K < \infty$ such that for any finite sequence $f_i \in \langle \{ h_I \} \rangle$,

1.3 $$\begin{aligned} \left\| \sum _i ( {{\,\mathrm{\mathbb {E}}\,}}_i f_i) e_i \right\| _{L^p(E)} \le K \left\| \sum _i f_i e_i \right\| _{L^p(E)} , \end{aligned}$$

where ${{\,\mathrm{\mathbb {E}}\,}}_i$ denotes the orthogonal projection onto $\langle \{ h_I: |I| \ge 2^{-i} \} \rangle$. In short, *E* with the symmetric basis $(e_i)$ satisfies $\mathcal {P}_p$ if the projection

$$\begin{aligned} C(f) = \sum _i ({{\,\mathrm{\mathbb {E}}\,}}_if_i) e_i, \end{aligned}$$

introduced by Capon [14], extends boundedly to $L^p(E)$, in which case we write $E\in \mathcal {P}_p$.

As noted by Capon for $1 \le r < \infty$ and $1< p < \infty$ we find that $\ell ^r$ with the standard unit vector basis satisfies $\mathcal {P}_p$, in short $\ell ^r\in \mathcal {P}_p$. By contrast, she provides examples showing that Capon’s projection *C* does not extend boundedly on the spaces

$$\begin{aligned} L^1(\ell ^r), \quad \text {and hence}\quad \ell ^r\notin \mathcal {P}_1, \quad 1< r < \infty . \end{aligned}$$

Now we turn to review Capon’s method of factoring operators $T:L^p(E) \rightarrow L^p(E)$ in the special case when $E \notin \mathcal {P}_p$. This case is of particular interest to our present paper, which is predominantly motivated by factoring operators on the following spaces

$$\begin{aligned} L^p(L^1) \text { with }1< p < \infty , \quad H^1(L^1), \quad L^1(H^1) . \end{aligned}$$

**Step 1** First, Capon reduces all matters to *E*-*diagonal* operators. For $\varepsilon > 0$, she determines exterior projectional factors $A:L^p(E)\rightarrow L^p(E)$ and $B:L^p(E)\rightarrow L^p(E)$ such that

$$\begin{aligned} ATB \text { is }\varepsilon \text {-close to an }E\text {-diagonal operator } S:L^p(E)\rightarrow L^p(E). \end{aligned}$$

Recall that *S* is *E*-diagonal, if there exists a sequence of operators $S_i:L^p \rightarrow L^p$ such that

$$\begin{aligned} S(f) = \sum _i ( S_if_i) e_i, \end{aligned}$$

where $f \in L^p(E)$ is given by $f = \sum _i f_i e_i$.

It is worth pointing out that the concept of *E*-diagonal operators corresponds to operator valued Haar multipliers. See Girardi [21] and Wark [60, 61].

**Step 2** After a further reduction, Capon verifies that one may assume that each of the operators $S_i$ is a Haar multiplier, that is,

$$\begin{aligned} S_i (h_I) = d_{I,i} h_I, \quad d_{I,i}\in \mathbb {R},\ I\in \mathcal {D},\ i \in \mathbb {N}. \end{aligned}$$

**Step 3a** Under the mild assumption that the space *E* does not contain a copy of $$\ell ^1$$, the operators $$S = (S_i) $$ admit a weakly convergent subsequence. Specifically, there exists a subsequence $$(n_i)$$ and $$S_\infty :L^p \rightarrow L^p$$ such that

1.4 $$\begin{aligned} \lim _{i\rightarrow \infty } \langle S_{n_i}(f), g \rangle = \langle S_{\infty }(f), g \rangle , \end{aligned}$$

for $$f\in L^p$$, $$g \in L^r$$, with $$1/p +1/r =1 $$ and $$ 1 \le p < \infty $$. One needs the additional assumption on *E* for the case $$p = 1$$. When $$1< p < \infty $$ the limiting $$S_\infty $$ exists without conditions on *E*.

Now Capon obtains a further block basis $(\tilde{h}_I)$ generating contractive exterior projectional factors $A, B:L^p \rightarrow L^p$ such that $\tilde{S}_\infty = AS_\infty B$ and $\tilde{S}_i = A S_{n_i} B$ are each Haar multipliers, and that the associated sequences of coefficients $(\tilde{d}_{I})$ and $(\tilde{d}_{I,i})$ satisfy joint stabilization conditions as follows: For $I\in \mathcal {D}$, the limit

$$\begin{aligned} \lambda (I) = \lim _{j\rightarrow \infty } \sum _{J \subset I,\, |J| =2^{-j}} \tilde{d}_J |J| \end{aligned}$$

exists and satisfies

$$\begin{aligned} | \tilde{d}_{I} |I| - \lambda (I) | < \varepsilon |I|^ 5, \quad \text {for }I\in \mathcal {D}. \end{aligned}$$

We then say that the Haar multiplier $\tilde{S}_\infty$ is stable. Simultaneously, $\tilde{S}_i$ is stable from level *i* onwards, meaning that

$$\begin{aligned} \lambda _i(I) = \lim _{j\rightarrow \infty } \sum _{J \subset I , \, |J| =2^{-j} } \tilde{d}_{J,i} |J| \end{aligned}$$

exists and satisfies

1.5 $$\begin{aligned} \bigl | \tilde{d}_{I,i} |I| - \lambda _i(I) \bigr | < \varepsilon |I|^5, \quad \text {for }I\in \mathcal {D}, |I| \le 2^{-i}, i\in \mathbb {N}. \end{aligned}$$

The set functions $\lambda (\cdot )$ and $\lambda _i(\cdot )$, as defined initially on dyadic intervals in [0, 1), may be extended uniquely to absolutely continuous measures, still denoted $\lambda$ and $\lambda _i$. Moreover, their respective densities form a bounded sequence in $L^{\infty }$.

**Step 3b** Capon’s proof continues with a close examination of the coefficients $(\tilde{d}_{I})$ and $(\tilde{d}_{I,i})$ and gives explicit formulas for the densities of $\lambda$ and $\lambda _i$.

For $t\in [0,1)$ and $k \in \mathbb {N}$ let the dyadic interval $I = I_k(t)$ be determined by the conditions $|I | = 2^{-k}$, $t \in I$. Then put

$$\begin{aligned} m_i (t) = \lim _{k\rightarrow \infty } \tilde{d}_{I_k(t), i} \quad \text {and}\quad m_\infty (t) = \lim _{k\rightarrow \infty } \tilde{d}_{I_k(t)}. \end{aligned}$$

In view of the above stability conditions, the limits used to form $m_i$ and $m_\infty$ are well defined and

$$\begin{aligned} \lambda (I) = \int _I m_\infty (t) dt, \quad \text {and}\quad \lambda _i (I) = \int _I m_i (t) dt, \quad \text {for }I\in \mathcal {D}, i\in \mathbb {N}. \end{aligned}$$

Moreover, $\sup _i \Vert m_i\Vert _{L^{\infty }} < \infty$, hence there exists a subsequence $(n_i)$ and $m\in L^\infty$ such that

1.6 $$\begin{aligned} \lim _{i\rightarrow \infty } \langle m_{n_i}, f \rangle = \langle m , f \rangle , \quad f \in L^1. \end{aligned}$$

**Step 3c** All further development of Capon’s proof depends on the relation between the two bounded functions $m_\infty$ and *m* just defined. Under the assumption that $E \notin \mathcal {P}_p$, she proves that the limits defining $m_\infty$ and *m* are necessarily interchangeable, and that

1.7 $$\begin{aligned} m(t) = \lim _{i\rightarrow \infty } \lim _{k\rightarrow \infty } \tilde{d}_{I_k(t), n_i} = \lim _{k\rightarrow \infty } \lim _{i\rightarrow \infty } \tilde{d}_{I_k(t), n_i} = m_\infty (t) . \end{aligned}$$

**Step 4** We continue assuming that $E\notin \mathcal {P}_p$. Exploiting the crucial identity $m = m_\infty$, Capon improves upon (1.4), and shows that strong convergence holds

1.8 $$\begin{aligned} \lim _{i\rightarrow \infty } \Vert \tilde{S}_{n_i}(g) - \tilde{S}_{\infty }(g)\Vert _{L^p} = 0, \qquad g\in L^ p. \end{aligned}$$

Consequently, for a suitable refinement of the subsequence $$(n_i) $$, still denoted $$(n_i) $$, we have

1.9 $$\begin{aligned} \left\| \sum _{i=1}^\infty \bigl ( \tilde{S}_{n_i}(f_i) e_i - \tilde{S}_{\infty }(f_i) e_i \bigr ) \right\| _{L^p(E)} < \varepsilon \Vert f\Vert _{L^p(E)}, \end{aligned}$$

where

$$\begin{aligned} f = \sum _i f_i e_i \in L^p(E). \end{aligned}$$

**Step 5.** Finally applying the Enflo–Maurey factorization to the limiting operator $\tilde{S}_{\infty }$, Capon readily obtains a simultaneous factorization for the entire sequence of convergent operators $(\tilde{S}_i )$.

#### Capon’s theorem on mixed norm spaces [13].

With the results published by Capon in 1983, the important classical Banach spaces

$$\begin{aligned} L^p(\ell ^q), \quad 1 \le p,q < \infty , \end{aligned}$$

were shown to be primary. At about the same time, Capon turned to the reflexive mixed norm spaces, and in 1982 published the proof [13] that the spaces

$$\begin{aligned} L^p(L^q ), \quad 1< p,q < \infty , \end{aligned}$$

are primary. Here Capon’s [13] approach utilizes that for $1< p,q < \infty$ the tensor product Haar system,

$$\begin{aligned} h_I \otimes h_J, \quad I, J \in \mathcal {D}, \end{aligned}$$

forms an unconditional basis in $L^p(L^q)$. Let $T:L^p(L^q) \rightarrow L^p(L^q)$ be a bounded linear operator and $\varepsilon > 0$. Capon [13] determines a scalar sequence $(a_{I,J}: I, J \in \mathcal {D})$ satisfying

$$\begin{aligned} |a_{I ,J}| \ge 1/2 \end{aligned}$$

and exterior projectional factors $$A,B:L^p(L^q) \rightarrow L^p(L^q)$$ such that for $$S = ATB $$ or $$S = A({{\,\textrm{Id}\,}}-T )B $$ we have

$$\begin{aligned} \left\| Sf - \sum _{I,J} a_{I , J} \langle h_I \otimes h_J, f \rangle h_I \otimes h_J / ( |I| \cdot |J | ) \right\| _{ L^p(L^q )} \le \varepsilon \Vert f\Vert _{L^p(L^q)}. \end{aligned}$$

Thus either *T* or ${{\,\textrm{Id}\,}}-T$ projectionally factors through a Haar multiplier with entries satisfying $|a_{I, J}| \ge 1/2$.

Capon’s exterior projectional factors *A*, *B* are determined by a bi-parameter block basis

$$\begin{aligned} \tilde{h}_{I , J} = \sum _{(K,L)\in \mathcal {A}_{I,J}} h_K \otimes h_L, \end{aligned}$$

where $(\mathcal {A}_{I,J}: \, I,J\in \mathcal {D})$ are pairwise disjoint collections of dyadic rectangles. We have

$$\begin{aligned} A:f&\mapsto \sum _{I, J \in \mathcal {D}} \langle \tilde{h}_{I,J}, f \rangle h_I\otimes h_J / (|I|\cdot |J|),\\ B:f&\mapsto \sum _{I, J \in \mathcal {D}} \langle h_I\otimes h_J, f \rangle \tilde{h}_{I,J} / (|I| \cdot |J |). \end{aligned}$$

We emphasize, that the block basis $(\tilde{h}_{I, J})$ satisfies Capon’s *local* product condition; it is, however, *not* of tensor product structure, and the unconditionality of the Haar system in $L^p(L^q)$ is a crucial ingredient in Capon’s [13] proof of the norm estimates

1.10 $$\begin{aligned} \Vert A \Vert \cdot \Vert B\Vert \le C_0(p,q). \end{aligned}$$

The constants $$C_0(p,q) $$ are unbounded as $$p \text { or } q \rightarrow 1 \text { or } \infty $$.

Exploiting Capon’s proof that $L^p(L^q)$, $1< p,q < \infty$ is primary, the third named author [49] obtained the analogous result for the bi-parameter Hardy space $H^1(H^1)$. However, proving primarity for any of the spaces

$$\begin{aligned} L^1(L^p ) \quad \text {or}\quad L^p(L^1), \qquad 1 \le p < \infty , \end{aligned}$$

is out of reach for the approach developed by Capon [13]. Only in 2022 the present authors [39] proved that the spaces $$L^1(L^p) $$, $$1< p < \infty $$ are primary.

Finally, neither Capon’s approach nor the one in our 2022 paper [39] applies to the limiting spaces

$$\begin{aligned} L^1(H^1) \quad \text {and}\quad H^1(L^1) . \end{aligned}$$

#### The present paper

The present paper is motivated by the open problem of whether primarity holds for any of the spaces

$$\begin{aligned} L^p(L^1),\ 1< p < \infty , \qquad L^1 (H^1) \qquad \text {or}\qquad H^1(L^1). \end{aligned}$$

One of our main results in this paper yields that Haar multipliers on these spaces are approximate projectional factors of $\alpha {{\,\textrm{Id}\,}}$ for some $\alpha \in \mathbb {R}$.

We approach these problems in a unified way, covering also the spaces $L^1(L^p)$ treated in [39]. We consider

$$\begin{aligned} \mathcal {F} = \{ L^p(L^q),\ H^p(H^q),\ L^p(H^q),\ H^p(L^q) : 1 \le p,q < \infty \}, \end{aligned}$$

and a subset

$$\begin{aligned} \mathcal {G} = \{ L^1(L^p),\ L^1(H^p),\ H^p(L^1) : 1 \le p < \infty \} . \end{aligned}$$

By way of example we show that *Capon’s projection* $\mathcal {C}$ associated to the bi-parameter Haar system is unbounded on $Z \in \mathcal {G}$, where

$$\begin{aligned} \mathcal {C}(f) = \sum _{I \in \mathcal {D}} \sum _{J \in \mathcal {D},\, |I| \le |J|} \frac{\langle h_I \otimes h_J, f \rangle }{|I|\cdot | J|} h_I \otimes h_J, \qquad \text {for}\ f\in \langle \{ h_I \otimes h_J \} \rangle . \end{aligned}$$

On the other hand, the bi-parameter Haar system is an unconditional basis in $Z \in \mathcal {F} \setminus \mathcal {G}$ and hence any bounded multiplier array acting on the bi-parameter Haar system gives rise to a continuous operator on the spaces in $\mathcal {F} {\setminus } \mathcal {G}$.

**Factoring through multipliers of the bi-parameter Haar system** Let $Z \in \mathcal {F}$ and let $D:Z\rightarrow Z$ be a bounded multiplier acting on the bi-parameter Haar system by a multiplier array, $(d_{I,J}: I, J \in \mathcal {D})$, that is,

$$\begin{aligned} D (h_I \otimes h_J ) = d_{I,J } h_I \otimes h_J, \quad I,J \in \mathcal {D} . \end{aligned}$$

Given the multiplier array $(d_{I,J})$, we prepare the statement of our main factorization theorems by defining the *factorization functionals* $\lambda$ and $\mu$.

1. Any two fixed integers $i, j \in \mathbb {N}_0$ give rise to a pavement of the unit square by the following collection of pairwise disjoint dyadic rectangles,
  $$\begin{aligned} \mathcal {R}(i, j) = \{ (I,J) \in \mathcal {D}\times \mathcal {D} : |I| = 2 ^{-i}, |J| =2^{-j} \}. \end{aligned}$$
  The cardinality of $\mathcal {R}(i, j)$ equals $2^ {i+j}$ and $|I| \cdot |J| = 2^ {-i-j}$ for $(I,J) \in \mathcal {R}(i, j)$. We use $\mathcal {R}(i, j)$ to define an average of the entries in the multiplier array as follows
  $$\begin{aligned} E_{i, j} = \sum _{(I,J)\in \mathcal {R}(i, j)} |I| \cdot |J| d_{I,J} = 2^{-i-j} \sum _{(I,J)\in \mathcal {R}(i, j)} d_{I,J}. \end{aligned}$$
2. We fix a non-principal ultrafilter $\mathcal {U}$ on $\mathbb {N}$ and form iterated row and column limits of the matrix $(E_{i,j})$ along $\mathcal {U}$. This gives rise to two distinct limits of the averages $E_{i, j}$
  1.11 $$\begin{aligned} \lambda _\mathcal {U}(D) = \lim _{j\rightarrow \mathcal {U}} \lim _{i\rightarrow \mathcal {U}} E_{i, j} \quad \text {and}\quad \mu _\mathcal {U}(D) = \lim _{i\rightarrow \mathcal {U}} \lim _{j\rightarrow \mathcal {U}} E_{i, j}. \end{aligned}$$

**Summary of the main results** The assertions of our factorization theorem for $D:Z \rightarrow Z$ are linked directly to $\lambda = \lambda _\mathcal {U}(D)$ and $\mu = \mu _\mathcal {U}(D)$, and critically depend on the norm of Capon’s projection on *Z*.

1. If Capon’s projection $\mathcal {C}$ does not extend to a bounded operator on *Z* then, we show that necessarily the iterated limits defining $\lambda$ and $\mu$ are interchangeable and we have equality,
  $$\begin{aligned} \lambda = \lim _{j\rightarrow \mathcal {U}} \lim _{i\rightarrow \mathcal {U}} E_{i, j} = \lim _{i\rightarrow \mathcal {U}} \lim _{j\rightarrow \mathcal {U}} E_{i, j} = \mu . \end{aligned}$$
  For $\varepsilon > 0$, there exist two faithful Haar systems $(\tilde{h}_I: I\in \mathcal {D})$ and $(\tilde{k}_J: J\in \mathcal {D})$ such that their respective tensor products
  $$\begin{aligned} \bigl ( \tilde{h}_I \otimes \tilde{k}_J : I, J\in \mathcal {D} \bigr ) \end{aligned}$$
  generate bounded exterior projectional factors
  1.12 $$\begin{aligned} \begin{aligned} A :f&\mapsto \sum _{I, J \in \mathcal {D}} \frac{\langle \tilde{h}_I \otimes \tilde{k}_J , f\rangle }{|I|\cdot |J|} h_I\otimes h_J,\\ B :f&\mapsto \sum _{I , J \in \mathcal {D}} \frac{\langle h_I\otimes h_J, f\rangle }{|I|\cdot |J|}\tilde{h}_I\otimes \tilde{k}_J. \end{aligned} \end{aligned}$$
  satisfying
  1.13 $$\begin{aligned} \Vert A D B - \mu {{\,\textrm{Id}\,}}\Vert _{L^p(L^1)} \le \varepsilon . \end{aligned}$$
2. If $\mathcal {C}$ extends to a bounded operator on *Z*, then for $\varepsilon > 0$, there exist of two faithful Haar systems $(\tilde{h}_I)$ and $(\tilde{k}_J)$ such that, by means of (1.12), their tensor products generate bounded exterior projectional factors $A,B:Z \rightarrow Z$ satisfying
  1.14 $$\begin{aligned} \bigl \Vert ADB - \bigl ( \lambda \mathcal {C} + \mu ({{\,\textrm{Id}\,}}_Z - \mathcal {C}) \bigr )\bigr \Vert < \varepsilon . \end{aligned}$$
  We may split the information encoded by (1.14) into the following three cases:
  - If $(\lambda , \mu )\notin \{(1,0), (0,1)\}$ then ${{\,\textrm{Id}\,}}_Z$ factors through
    1.15 $$\begin{aligned} \lambda \mathcal {C} + \mu ({{\,\textrm{Id}\,}}_Z - \mathcal {C}) \quad \text {or}\quad (1-\lambda ) \mathcal {C} + (1-\mu ) ({{\,\textrm{Id}\,}}_Z - \mathcal {C}). \end{aligned}$$
    We note in passing that the identities $\lambda _\mathcal {U}({{\,\textrm{Id}\,}}- D) = 1-\lambda _\mathcal {U}(D)$ and $\mu _\mathcal {U}({{\,\textrm{Id}\,}}- D) = 1-\mu _\mathcal {U}(D)$ are used in the second alternative of (1.15).
  - If $$(\lambda , \mu ) = (1,0)$$ then
    $$\begin{aligned} \Vert ADB - \mathcal {C}\Vert < \varepsilon . \end{aligned}$$
  - If $$ (\lambda , \mu ) = (0,1)$$ then
    $$\begin{aligned} \Vert ADB - ({{\,\textrm{Id}\,}}_Z - \mathcal {C})\Vert < \varepsilon . \end{aligned}$$

**Remarks:**

- Exploiting bi-tree semi-stabilization, related in spirit to the Semenov–Uksusov [58] characterization of bounded Haar multipliers on $$L^1 $$, and the concentration of measure phenomenon for empirical processes, the full combinatorial and probabilistic force of our efforts is directed at proving the existence of two faithful Haar systems such that in (1.12), their respective tensor products generate bounded exterior projectional factors *A*, *B*, for which the factorization estimates (1.13) respectively (1.14) hold true.
- The tensor product structure of the block basis defining *A*, *B* is then crucial in our proof that
  1.16 $$\begin{aligned} \Vert A \Vert \cdot \Vert B\Vert \le 1, \end{aligned}$$
  independent of $Z \in \mathcal {F}$. This should be contrasted with Capon’s constants $C_0(p,q)$ in (1.10) which are unbounded as $p \text { or } q \rightarrow 1 \text { or } \infty$.

**Haar system Hardy spaces** Initially, our work was concerned with the factorization of operators defined on the well known spaces in the family

$$\begin{aligned} \mathcal {F} = \{ L^p(L^q),\ H^p (H^q),\ L^p(H^q),\ H^p(L^q) : 1 \le p,q < \infty \}. \end{aligned}$$

In the present paper, we exhibit a novel method for constructing tensor product bases and exterior projectional factors on a class of bi-parameter function spaces that includes the family $\mathcal {F}.$ This opened the door for a *unified approach* to solving factorization problems.

In this paper, we consider operators on the class of bi-parameter function spaces called *Haar System Hardy Spaces*, denoted $\mathcal {H} \mathcal {H}(\delta ^2)$. See Definition 2.8. The class of Haar system Hardy spaces is broad enough to contain the family $\mathcal {F}$, and is of independent interest. Let

$$\begin{aligned} Z \in \mathcal {H}\mathcal {H}(\delta ^2). \end{aligned}$$

The main result of the present paper asserts that for any bounded multiplier operator $D:Z \rightarrow Z$ acting by an array $(d_{I,J})$ on the bi-parameter Haar system $(h_I\otimes h_J)$ at least one of the following statements is true:

(i) $D:Z \rightarrow Z$ is an approximate projectional factor of $\lambda {{\,\textrm{Id}\,}}_Z$,
(ii) $D:Z \rightarrow Z$ is an approximate projectional factor of $\lambda \mathcal {C} + \mu ({{\,\textrm{Id}\,}}_Z - \mathcal {C})$, where $\mathcal {C}$ denotes Capon’s projection.

The first assertion holds when $\mathcal {C}$ does not extend to a bounded operator on *Z*. The second one holds when $\mathcal {C}$ is bounded on *Z*. The factorization functionals $\lambda = \lambda _\mathcal {U} (D)$ and $\mu = \mu _\mathcal {U} (D)$, depending on the ultrafilter $\mathcal {U}$ and the multiplier array $(d_{I,J})$, are predetermined by (1.11).

### Organization of the paper

In Sect. 2 we state the main results of the paper and discuss in detail their usefulness in proving that (some) bi-parameter Haar system Hardy spaces are primary.

Section 3 is devoted to presenting the main combinatorial and probabilistic results, forming our novel stabilization method for the entries of a multiplier array $(d_{I,J}).$ The inductive proof is facilitated by showing that our stability estimates persist under specific iterated blockings of faithful Haar systems.

In Sect. 4 we present the basic properties of Haar system Hardy spaces, particularly the boundedness of exterior projectional factors generated by tensor product Haar systems.

In Sect. 5 we investigate the pointwise multiplication operators generated by a stabilized Haar multiplier *D*. Those play a mediating role between the stabilized multiplier array and its derived factorization functionals. We thus obtain the crucial identity $\lambda _{\mathcal {U}} = \mu _{\mathcal {U}}$, when the operator norm of Capon’s projection $\mathcal {C}$ is unbounded on *Z*.

## Main results

In this section, we introduce the necessary notation, state our main results, and lay out the pathway towards proving them.

### Reduction of classes of operators: main results for $L^p(L^1)$, $L^1(H^1)$, and $H^1(L^1)$

A Banach space *X* is called *primary* if whenever $X\simeq Y\oplus Z$, where *Y*, *Z* are closed subspaces of *X*, then $X\simeq Y$ or $X\simeq Z$. We focus on Banach spaces of functions of two parameters. The spaces $L^p(L^q)$, $1<p,q<\infty$ were proved to be primary by Capon in [13]. $H^1(H^1)$ was proved to be primary by the third author in [49]. Among spaces of this type that involve $L^1$, only $L^1(L^p)$ was proved to be primary in [39]. The purpose of this paper is to prove results about the factoring properties of classes of operators of Banach spaces in a general class, called Haar system Hardy spaces, that contains the mixed Lebesgue-Hardy spaces $L^p(L^1)$, $H^1(L^1)$, $L^1(H^1)$, and explain how these results fit within a scheme of proof of the primarity of these spaces, which is not yet known.

We begin by recalling the necessary notions to formally state our main results.

#### Notation 2.1

Let $$\mathbb {N} = \{1,2,\ldots \}$$ and $$\mathbb {N}_0 = \mathbb {N}\cup \{0\}$$.

- For any set of vectors *V*, we denote its linear span by $\langle V \rangle$.
- We denote by $\mathcal {D}$ the collection of all dyadic intervals in [0, 1), namely
  $$\begin{aligned} \mathcal {D} = \left\{ \left[ \frac{i-1}{2^j},\frac{i}{2^j}\right) : j\in \mathbb {N}_0,\ 1\le i\le 2^j\right\} . \end{aligned}$$
  Moreover, we put $\mathcal {D}_j = \{ I \in \mathcal {D}: |I| = 2^{-j}\}$.
- For $I\in \mathcal {D}$, let $I^+$ denote the left half of *I* and $I^-$ denote the right half of *I*, i.e., $I^+$ is the largest $J\in \mathcal {D}$ with $J\subsetneq I$ and $\inf J = \inf I$, and $I^- = I\setminus I^+\in \mathcal {D}$.
- The Haar system $(h_I)_{I\in \mathcal {D}}$ is defined by
  $$\begin{aligned} h_I = \chi _{I^+} - \chi _{I^-}, \qquad I\in \mathcal {D} \end{aligned}$$
  and we denote by $\mathcal {V}(\delta )$ its linear span.
- We let $(k_J:J\in \mathcal {D})$ be a copy of the Haar system $(h_I:I\in \mathcal {D})$ and define for $I,J\in \mathcal {D}$
  $$\begin{aligned} h_I\otimes k_J:[0,1)^2\rightarrow \mathbb {R}, \quad (s,t)\rightarrow h_I(s)k_J(t). \end{aligned}$$
  We call $(h_I\otimes k_J:I,J\in \mathcal {D})$ the *bi-parameter Haar system* and denote by $\mathcal {V}(\delta ^2)$ its linear span. More generally for $f,g:[0,1)\rightarrow \mathbb {R}$ we write
  $$\begin{aligned} f\otimes g: [0,1)^2\rightarrow \mathbb {R}, \quad (s,t)\mapsto f(s)g(t). \end{aligned}$$
- For $f,g\in \mathcal {V}(\delta )$, or $f,g\in \mathcal {V}(\delta ^2)$ we define
  $$\begin{aligned} \langle f,g \rangle = \int _0^1 f(s)g(s) \,d s, \quad \text {respectively}\quad \langle f,g \rangle = \int _0^1\int _0^1 f(s,t) g(s,t)\, \textrm{d} s \textrm{d} t. \end{aligned}$$
- A linear operator $D:\mathcal {V}(\delta )\rightarrow \mathcal {V}(\delta )$, or $D:\mathcal {V}(\delta ^2)\rightarrow \mathcal {V}(\delta ^2)$, is called a *Haar multiplier* if every one-parameter Haar vector $h_I$, respectively if every bi-parameter Haar vector $h_I\otimes k_J$ is an eigenvector of *D*. We denote the space of all Haar multipliers by ${\text {HM}}(\delta )$ and ${\text {HM}}(\delta ^2)$, respectively. The eigenvalues of a Haar multiplier *D* are called the *coefficients of D*.

For $1\le p < \infty$ let $L^p$ denote the Banach space of *p*-integrable functions on [0, 1) with mean zero, i.e., the closure of $\mathcal {V}(\delta )$ under the $\Vert \cdot \Vert _p$-norm. For $1\le p,q < \infty$ we define the mixed-norm Lebesgue space $L^p(L^q)$ to be the completion of $\mathcal {V}(\delta ^2)$ under the norm

2.1 $$\begin{aligned} \Vert f\Vert _{L^p(L^q)} = \left( \int _0^1 \left( \int _0^1|f(t,s)|^q\textrm{d} s \right) ^{p/q}\textrm{d} t \right) ^{1/p}. \end{aligned}$$

More generally, for a given Banach space *E* we define the Bochner-Lebesgue space $L^p(E)$ to consist of all Bochner-measurable *E*-valued functions $f:[0,1]\rightarrow E$ for which

$$\begin{aligned} \Vert f\Vert _{L^p(E)} := \left( \int _0^1 \Vert f(t) \Vert _E^p \textrm{d} t \right) ^{1/p} < \infty . \end{aligned}$$

Endowing $L^p(E)$ with the norm $\Vert \cdot \Vert _{L^p(E)}$ gives rise to a Banach space. For $E = L^q$, we obtain (2.1). If $E = H^1$ we obtain $L^1(H^1)$ where $H^1$ denotes the completion of $\mathcal {V}(\delta )$ under the norm

2.2 $$\begin{aligned} \left\| \sum _{I\in \mathcal {D}} a_{I} h_I \right\| _{H^1} = \int _{0}^{1} {{\,\mathrm{\mathbb {E}}\,}}\left| \sum _{I\in \mathcal {D}} \sigma _I a_{I} h_I(s) \right| \textrm{d} s, \end{aligned}$$

where $\sum _{I\in \mathcal {D}} a_{I} h_I \in \mathcal {V}(\delta )$ and where ${{\,\mathrm{\mathbb {E}}\,}}$ denote the expectation with respect to the Haar measure over all $\sigma = (\sigma _I)_{I\in \mathcal {D}}\in \{\pm 1\}^\mathcal {D}$.

Next, we define the vector-valued dyadic Hardy space $H^1(L^1)$ as the completion of $\mathcal {V}(\delta ^2)$ under the norm

2.3 $$\begin{aligned} \left\| \sum _{I,J\in \mathcal {D}} a_{I,J} h_I\otimes k_J \right\| _{H^1(L^1)} = \int _{0}^{1} \int _{0}^{1} {{\,\mathrm{\mathbb {E}}\,}}\left| \sum _{I,J\in \mathcal {D}} \sigma _I a_{I,J} h_I(s) k_J(t) \right| \textrm{d}t\textrm{d}s. \end{aligned}$$

By evaluating the inner expectation with respect to $\sigma$ using Khintchine’s inequality, we see that the above norm is equivalent to

2.4 $$\begin{aligned} \int _0^1\int _0^1 \left( \sum _{I\in \mathcal {D}} \left( \sum _{J\in \mathcal {D}}a_{I,J}k_J(s) \right) ^2 h^2_I(t) \right) ^{1/2} \textrm{d}t\textrm{d}s. \end{aligned}$$

We link the above expression to the literature on vector-valued Hardy spaces $H^1(E)$. Given an $L^1(E)$-convergent dyadic *E*-valued martingale $f = (f_j)_{j=0}^{\infty }$ we define its $H^1(E)$-norm to be

2.5 $$\begin{aligned} \Vert f\Vert _{H^1(E)} = {{\,\mathrm{\mathbb {E}}\,}}\int _{0}^{1} \left\| \sum _{j=0}^{\infty } \sigma _j (f_{j+1}(t) - f_j(t)) \right\| _E \textrm{d}t, \end{aligned}$$

where now, ${{\,\mathrm{\mathbb {E}}\,}}$ denote the expectation with respect to the Haar measure over $(\sigma _j)_{j\in \mathbb {N}}\in \{\pm 1\}^\mathbb {N}$. For $E = L^1$, the expression in (2.5) coincides with the one in (2.3). We refer to [53, 24] and [57].

We study the factorization properties of operators on such spaces. Factors of the identity are by now classical and have been considered in varying contexts, e.g., in [17, 34, 39], and [32]. Specifically, in [39] (and elsewhere), factors of the identity were used to show that certain Banach spaces are primary. Our main result, Theorem 2.3, is a significant step towards the proof of primarity of the spaces $L^p(L^1)$, $H^1(L^1)$, $L^1(H^1)$. This is explained in detail, using the language of Definition 2.2 in Remark 2.4 and Remark 2.5.

#### Definition 2.2

Let *X* be a Banach space. Denote by $\mathcal {L}(X)$ the space of bounded linear operators on *X* and denote by ${{\,\textrm{Id}\,}}:X\rightarrow X$ the identity map.

- Let $S,T\in \mathcal {L}(X)$. We say that *T* *is a* *C*-*factor of* *S* if there exist operators $A,B:X\rightarrow X$ with $\Vert A\Vert \cdot \Vert B\Vert \le C$ such that $S = ATB$. In this case, we also say that *S* *C*-factors through *T*.
- Let $S,T\in \mathcal {L}(X)$. We say that *T* *is a* *C*-*projectional factor with error* $\eta \ge 0$ *of* *S* if there exist operators $A,B:X\rightarrow X$ with $\Vert A\Vert \Vert B\Vert \le C$, $AB = {{\,\textrm{Id}\,}}$ and such that $\Vert S - ATB\Vert \le \eta$. Alternatively, we say that *S* projectionally *C*-factors through *T* with error $\eta$.
- Let $\mathcal {A}$, $\mathcal {B}$ be subclasses of $\mathcal {L}(X)$, and $C>0$. We say that $\mathcal {A}$ *approximately* *C*-*projectionally reduces to* $\mathcal {B}$ if for every *T* in $\mathcal {A}$ and $\eta >0$ there exists *S* in $\mathcal {B}$ such that *T* is a *C*-projectional factor with error $\eta$ of *S*.
- Let $\mathcal {A}$ be a subclass of $\mathcal {L}(X)$. We say that $\mathcal {A}$ *has the* *C*-*primary factorization property* if for any operator $T\in \mathcal {A}$ we have that either *T* or ${{\,\textrm{Id}\,}}-T$ is a *C*-factor of ${{\,\textrm{Id}\,}}$.
- We say that *X* *has the* *C*-*primary factorization property* if $\mathcal {L}(X)$ has the *C*-primary factorization property.

If we omit the constant $C>0$ in any of the above properties, we understand this to mean that this property is satisfied for some $0< C < \infty$.

The versatility of factors of the identity is reflected in the following definition, applicable to any Banach space *X*. Put $\mathcal {M}_X = \{T\in \mathcal {L}(X): T \text {is not a factor of} {{\,\textrm{Id}\,}}\}$. This set is closed in the operator topology and under multiplication from the right and the left by bounded operators. If it happens to be closed under addition, then it is a maximal two-sided ideal in $\mathcal {L}(X)$. Note that items (a) to (e) in Definition 2.2 can be stated for an arbitrary unital Banach algebra in the place of $\mathcal {L}(X)$.

Below is the main theorem of this paper for Banach spaces of functions of two parameters that involve $L^1$. It follows by combining Theorems 2.14 and 2.10.

#### Theorem 2.3

Let *Z* be one of the spaces $L^1(L^p)$, $L^p(L^1)$, $1\le p<\infty$, $L^1(H^1)$, or $H^1(L^1)$ and denote by $\textrm{HM}(Z)$ the unital subalgebra of $\mathcal {L}(Z)$ of all bounded Haar multipliers on *Z*. Then, $\textrm{HM}(Z)$ approximately 1-projectionally reduces to the class $\{\lambda {{\,\textrm{Id}\,}}:\lambda \in \mathbb {R}\}$ of scalar operators. In particular, $\textrm{HM}(Z)$ has the *C*-primary factorization property, for every $C > 2$.

We make some elementary remarks that we then use to put our main result into context.

#### Remark 2.4

- In Definition 2.2 (b) note that *BA* is a projection onto a subspace of *X* that is isomorphic to *X* and, thus, the term “projectional factor” is used.
- If *T* is a *C*-projectional factor of *S* with error $\eta \ge 0$ then also ${{\,\textrm{Id}\,}}-T$ is a *C*-projectional factor of ${{\,\textrm{Id}\,}}-S$ with error $\eta$.
- By [39, Proposition 2.3], the relation in Definition 2.2 (c) satisfies a certain transitivity property; if $\mathcal {A}_1$ approximately *C*-projectionally reduces to $\mathcal {A}_2$ and $\mathcal {A}_2$ approximately *D*-projectionally reduces to $\mathcal {A}_3$, then $\mathcal {A}_1$ approximately *CD*-projectionally reduces to $\mathcal {A}_3$.
- It is straight forward that for any Banach space *X* the class $\{\lambda {{\,\textrm{Id}\,}}:\lambda \in \mathbb {R}\}$ of scalar operators has the 2-primary factorization property.
- For $X = L^p$, $1\le p\le \infty$, the class of pointwise multipliers on *X*, $\textrm{PM}(X) = \{M_g\in \mathcal {L}(X): g\in L^\infty \}$, approximately 1-projectionally reduces to the class of scalar operators. This follows from the fact that for every measurable subset *A* of [0, 1) of positive measure, $L^p(A,P|_A)$ is isometrically isomorphic to $L^p$ and 1-complemented in $L^p$.

Some explanation is due about the placement of Theorem 2.3 within the context or primarity of Banach spaces.

#### Remark 2.5

Assume that a Banach space *X* satisfies the following two properties.

- *X* satisfies the accordion property, i.e., $X\simeq \ell ^p(X)$, for some $1\le p\le \infty$, or $X\simeq c_0(X)$.
- *X* satisfies the primary factorization property.

Then, *X* is primary.

Condition (a) was introduced by Pełczyński in [55] to prove that the Banach spaces $\ell ^p$, $1\le p<\infty$, and $c_0$ are prime. The observation that (a) and (b) yields primarity was implicitly made by Lindenstrauss and Pełczyński in [42] where they used it to deduce that *C*[0, 1] is primary. For an argument justifying Remark 2.5, see, e.g., [39, Proposition 2.4].

Sometimes, proving (b) goes through several steps that involve the relation introduced in Definition 2.2 (c). Thus, condition (b) is frequently replaced with the following equivalent condition.

(b’) there are classes of operators $\mathcal {L}(X) = \mathcal {A}_1$,...,$\mathcal {A}_n$ in $\mathcal {L}(X)$ such that $\mathcal {A}_i$ approximately projectionally reduces to $\mathcal {A}_{i+1}$, for $1\le i<n$.

Condition (b^′^) was used implicitly, e.g., by Maurey via Enflo [45], who proved that in $X = L^p$, $\mathcal {L}(X)$ approximately 1-projectionally reduces to the class of pointwise multipliers. By Remark 2.4 (e), this class approximately projectionally reduces to the class of scalar operators, which has the primary factorization property. A similar process was implicitly used by Alspach, Enflo, and Odell [1] to give a simpler proof that $X = L^p$, $1<p<\infty$ is primary, by reducing $\mathcal {L}(X)$ to the class of Haar multipliers. They then used ideas of Gamlen and Gaudet from [19] and exploited unconditionality to further reduce that class to the one of scalar operators. Capon used more elaborate versions of this scheme for $\ell ^p(L^1)$, $1<p<\infty$, $L^p(\ell ^r)$ and $L^p(L^r)$, $1<p,r<\infty$ [11–13]. The authors of this paper also followed this scheme in [39] to prove that $L^1(L^p)$, $1< p <\infty$, is primary.

We conclude this subsection with a comment on a potential proof of primarity of the spaces $L^p(L^1)$, $L^1(H^1)$, and $H^1(L^1)$. Let *Z* denote one of them. The last missing step is to prove that $\mathcal {L}(Z)$ approximately projectionally reduces to $\textrm{HM}(Z)$. Indeed, if this is the case, then, by the transitivity property explained in Remark 2.4(c), $\mathcal {L}(Z)$ would approximately projectionally reduce to the class of scalar operators. Because *Z* has the accordion property, by Remark 2.5, *Z* would be primary. For instance, the accordion property of $H^1(L^1)$ is proved in the exact same manner as for the scalar-valued case $H^1$ treated in Maurey’s paper [46, Paragraph 4.25, page 114], using equation (2.4).

### Haar system Hardy spaces

At this point, we introduce a new class of bi-parameter spaces called *Haar System Hardy Spaces*. This is a broad class of independent interest, and it includes $L^1(L^p)$, $L^p(L^1)$, $1\le p < \infty$, $L^1(H^1)$, and $H^1(L^1)$; for the spaces in this class, we prove a generalization of Theorem 2.3 (see Theorem 2.14).

We recall the notion of Haar system spaces from [39]. This variation of classical rearrangement invariant Banach spaces includes all such spaces for which the Haar system is a basis, as well as the Cantor space $C(\Delta )$, defined as the closure of $\mathcal {V}(\delta )$ in $L^\infty$, equipped with the $L^\infty$-norm.

#### Definition 2.6

A *Haar system space* *W* is the completion of

$$\begin{aligned} \mathcal {V}_1(\delta ) = \langle \{\chi _{[0,1}\}\cup \{ h_I : I\in \mathcal {D}\} \rangle = \langle \{\chi _I : I\in \mathcal {D}\} \rangle \end{aligned}$$

under a norm $\Vert \cdot \Vert$ that satisfies the following properties.

- If *f*, *g* are in $\mathcal {V}_1(\delta )$ and |*f*|, |*g*| have the same distribution then $\Vert f\Vert = \Vert g\Vert$.
- $$\Vert \chi _{[0,1)}\Vert = 1$$.

We denote the class of Haar system spaces by $\mathcal {H}(\delta )$.

The following proposition lists some basic properties of Haar system spaces. Properties (i), (ii), and (iii) were shown in [39, Proposition 2.13], (iv) follows from (iii), and (v) from (iv).

#### Proposition 2.7

Let *W* be a Haar system space.

(i) For every $f\in Z = \langle \{\chi _I:I\in \mathcal {D}\}\rangle$ we have $\Vert f\Vert _{L_1} \le \Vert f\Vert \le \Vert f\Vert _{L_\infty }$.
(ii) The Haar system, in the usual linear order, is a monotone Schauder basis of *W*.
(iii) $Z = \langle \{\chi _I:I\in \mathcal {D}\}\rangle$ naturally coincides with a subspace of $W^*$ and its closure $\overline{Z}$ in $W^*$ is also a Haar system space.
(iv) For $f\in W$, we have
  $$\begin{aligned} \Vert f\Vert = \sup _{g\in Z, \Vert g\Vert _*\le 1} \int f(x)g(x)\,dx \end{aligned}$$
  where $\Vert \cdot \Vert _*$ is the dual norm on $W^*$.
(v) If $$f,g\in W$$, $$|f| \le |g|$$ then $$\Vert f\Vert \le \Vert g\Vert $$.

Given a space $X\in \mathcal {H}(\delta )$, we can define a one-parameter Hardy space as follows. Let ${{\,\mathrm{\mathbb {E}}\,}}$ denote the expectation with respect to the Haar measure over all $(\sigma _I)_{I\in \mathcal {D}}$ in $\{-1,1\}^\mathcal {D}$. The Haar system Hardy space $X(\varvec{\sigma })$ is defined as the completion of $\mathcal {V}(\delta )$ with the following norm. If $f = \sum _{I\in \mathcal {D}}a_Ih_I\in \mathcal {V}(\delta )$ then

$$\begin{aligned} \Vert f\Vert _{X(\varvec{\sigma })} = \left\| {{\,\mathrm{\mathbb {E}}\,}}\left| \sum _{I\in \mathcal {D}}\sigma _Ia_Ih_I\right| \right\| _X = \left\| t\mapsto {{\,\mathrm{\mathbb {E}}\,}}\left| \sum _{I\in \mathcal {D}}\sigma _Ia_Ih_I(t)\right| \right\| _X. \end{aligned}$$

We define and investigate the two-parameter version that involves two spaces *X*, *Y* in $\mathcal {H}(\delta )$.

#### Definition 2.8

- For *X*, $Y\in \mathcal {H}(\delta )$ we define the *bi-parameter Haar system space* *X*(*Y*) as the completion of $\mathcal {V}(\delta ^2)$ under the following norm. If $f = \sum _{I,J}a_{I,J}h_I\otimes k_J$ then
  $$\begin{aligned} \Vert f\Vert _{X(Y)} = \left\| s\mapsto \left\| t\mapsto \sum _{I,J\in \mathcal {D}} a_{I,J}h_I(s) k_J(t) \right\| _Y \right\| _X. \end{aligned}$$
  This is well defined, since, if $f\in \mathcal {V}(\delta ^2)$ then $\Vert t\mapsto f(\cdot ,t)\Vert _Y$ is in $\mathcal {V}_1(\delta )$, but, obviously, may not be in $\mathcal {V}(\delta )$.
- Let $\varvec{\sigma }_{1,1} = \{\pm 1\}^\mathcal {D}\times \{\pm 1\}^\mathcal {D}$, $\varvec{\sigma }_{0,0} = \{1\}^\mathcal {D}\times \{1\}^\mathcal {D}$, $\varvec{\sigma }_{0,1} = \{1\}^\mathcal {D}\times \{\pm 1\}^\mathcal {D}$, and $\varvec{\sigma }_{1,0} = \{\pm 1\}^\mathcal {D}\times \{1\}^\mathcal {D}$. Denote $\varvec{\Sigma } = \{\varvec{\sigma }_{1,1},\varvec{\sigma }_{0,0},\varvec{\sigma }_{0,1},\varvec{\sigma }_{1,0}\}$. For a fixed $\varvec{\sigma }\in \varvec{\Sigma }$ let ${{\,\mathrm{\mathbb {E}}\,}}$ denote the expectation with respect to the Haar product measure over all $\sigma = \big ((\sigma ^{(1)}_I)_{I\in \mathcal {D}},(\sigma ^{(2)}_J)_{J\in \mathcal {D}}\big )$ in $\varvec{\sigma }$.
- For *X*, $Y\in \mathcal {H}(\delta )$, $\varvec{\sigma }\in \varvec{\Sigma }$ we define the *bi-parameter Haar system Hardy space* $Z = Z(\varvec{\sigma },X,Y)$ as the completion of $\mathcal {V}(\delta ^2)$ under the following norm. If $f = \sum _{I,J}a_{I,J}h_I\otimes k_J$ then
  $$\begin{aligned} \Vert f\Vert _Z&= \left\| {{\,\mathrm{\mathbb {E}}\,}}\left| \sum _{I,J\in \mathcal {D}} \sigma ^{(1)}_I\sigma ^{(2)}_J a_{I,J}h_I\otimes k_J \right| \right\| _{X(Y)}\\&= \left\| s\mapsto \left\| t\mapsto {{\,\mathrm{\mathbb {E}}\,}}\left| \sum _{I,J\in \mathcal {D}} a_{I,J}\sigma ^{(1)}_I\sigma ^{(2)}_J h_I(s) k_J(t) \right| \right\| _Y \right\| _X. \end{aligned}$$

We denote by $\mathcal{H}\mathcal{H}(\delta ^2)$ the class of all bi-parameter Haar system Hardy spaces.

#### Remark 2.9

By specializing the Haar system Hardy spaces, many classical spaces are contained in $\mathcal{H}\mathcal{H}(\delta ^2)$. Let $Z = Z(\varvec{\sigma },X,Y)\in \mathcal{H}\mathcal{H}(\delta ^2)$.

- If $\varvec{\sigma } = \varvec{\sigma }_{0,0} = \{1\}^\mathcal {D}\times \{1\}^\mathcal {D}$, then for $f\in Z$,
  $$\begin{aligned} \Vert f\Vert _{Z} = \left\| \sum _{I,J\in \mathcal {D}} a_{I,J} h_I\otimes k_J \right\| _{X(Y)}. \end{aligned}$$
  Thus, the two-parameter Haar system Hardy space *Z* becomes the two-parameter Haar system space *X*(*Y*). Further specifying $X=L^p$, $Y=L^q$, $1\le p,q < \infty$, we obtain the classical two parameter Lebesgue-space $L^p(L^q)$.
- If $\varvec{\sigma } = \varvec{\sigma }_{1,1} = \{\pm 1\}^\mathcal {D}\times \{\pm 1\}^\mathcal {D}$, then for $f\in Z$,
  $$\begin{aligned} \Vert f\Vert _{Z} = \left\| {{\,\mathrm{\mathbb {E}}\,}}\left| \sum _{I,J\in \mathcal {D}} \sigma _I^{(1)}\sigma _J^{(2)}a_{I,J} h_I\otimes k_J \right| \right\| _{X(Y)}. \end{aligned}$$
  Specializing $X=L^p$, $Y=L^q$, $1\le p,q < \infty$, we obtain the classical bi-parameter dyadic Hardy space $H^p(H^q)$. In general, by the monotonicity of the norms in *X*, *Y* (see Proposition 2.7 (v)) and Khintchine’s inequality, we obtain that $\Vert \cdot \Vert _Z$ is equivalent to the square norm given for $f = \sum _{I,J\in \mathcal {D}} a_{I,J} h_I\otimes k_J$ by
  $$\begin{aligned} \left\| \left( \sum _{I,J\in \mathcal {D}} a_{I,J}^2 h_I^2\otimes k_J^2 \right) ^{1/2} \right\| _{X(Y)}. \end{aligned}$$
  Further specializing $X=L^p$, $Y=L^q$, $1\le p,q < \infty$, we obtain the classical bi-parameter dyadic Hardy space $H^p(H^q)$.
- If $\varvec{\sigma } = \varvec{\sigma }_{0,1} = \{1\}^\mathcal {D}\times \{\pm 1\}^\mathcal {D}$, then for $f = \sum _{I,J\in \mathcal {D}} a_{I,J} h_I\otimes k_J$, we have
  $$\begin{aligned} \Vert f\Vert _{Z} = \left\| {{\,\mathrm{\mathbb {E}}\,}}\left| \sum _{I,J\in \mathcal {D}} a_{I,J} \sigma _J^{(2)} h_I\otimes k_J \right| \right\| _{X(Y)}. \end{aligned}$$
  Specializing $X=L^p$, $Y=L^q$, $1\le p,q < \infty$, we obtain the classical space $L^p(H^q)$. In general, by Khintchine’s inequality and the monotonicity of the norms in *X*, *Y*, we obtain that the norm $\Vert \cdot \Vert _{Z}$ is equivalent to the partial square function norm given by
  $$\begin{aligned} \left\| \left( \sum _{J\in \mathcal {D}} \left( \sum _{I\in \mathcal {D}} a_{I,J} h_I \right) ^2\otimes k_J^2 \right) ^{1/2} \right\| _{X(Y)}. \end{aligned}$$
- If $\varvec{\sigma } =\varvec{\sigma }_{1,0} = \{\pm 1\}^\mathcal {D}\times \{1\}^\mathcal {D}$, then for $f = \sum _{I,J\in \mathcal {D}} a_{I,J} h_I\otimes k_J$, we have
  $$\begin{aligned} \Vert f\Vert _{Z} = \left\| {{\,\mathrm{\mathbb {E}}\,}}\left| \sum _{I,J\in \mathcal {D}} a_{I,J} \sigma _I^{(1)} h_I\otimes k_J \right| \right\| _{X(Y)}. \end{aligned}$$
  Specializing $X=L^p$, $Y=L^q$, $1\le p,q < \infty$, we obtain the classical space $H^p(L^q)$. In general, using Khintchine’s inequality and the monotonicity of the norms in *X*, *Y*, we obtain that $\Vert \cdot \Vert _{Z}$ is equivalent to the partial square function norm given by
  $$\begin{aligned} \left\| \sum _{I\in \mathcal {D}} \left( \left( \sum _{J\in \mathcal {D}} a_{I,J} k_J \right) ^2\otimes h_I^2 \right) ^{1/2} \right\| _{X(Y)}. \end{aligned}$$

In Theorem 2.10 below, we give necessary conditions for the unboundedness of the Capon projection. In particular, the Capon projection is unbounded on the following spaces: $L^1(L^p)$, $L^p(L^1)$, $L^1(H^1)$, $H^1(L^1)$.

#### Theorem 2.10

Let $Z = Z(\varvec{\sigma },X,Y)\in \mathcal{H}\mathcal{H}(\delta ^2)$ and suppose that

- $(\sigma _I^{(1)})$ is constant and either $\Vert \cdot \Vert _X\sim \Vert \cdot \Vert _{L^1}$ or $\Vert \cdot \Vert _X\sim \Vert \cdot \Vert _{L^\infty }$, or
- $(\sigma _J^{(2)})$ is constant and either $\Vert \cdot \Vert _Y\sim \Vert \cdot \Vert _{L^1}$ or $\Vert \cdot \Vert _Y\sim \Vert \cdot \Vert _{L^\infty }$,

then $\mathcal {C}:Z\rightarrow Z$ is unbounded.

Before proving the above theorem, we give an overview of classical examples and the boundedness of the Capon projection in the Table 1. The rows of the table correspond to the norm of the space *X* and the letters “c” and “i” indicate whether the sequence $(\sigma _I^{(1)})$ is constant or independent. Similarly, the columns correspond to the norm of the space *Y*, and the letters “c” and “i” indicate whether the sequence $(\sigma _J^{(2)})$ is constant or independent. The entries “B”/“U” in the Table 1 mean the Capon projection is bounded/unbounded on the space $Z = Z(\mathbf {\sigma },X,Y)$.

**Table 1:** Capon projection table

	$L^1$, c	$L^1$, i	$L^p$	$L^q$	$L^{\infty }$, i	$L^{\infty }$, c
$L^1$, c	U	U	U	U	U	U
$L^1$, i	U	B	B	B	B	U
$L^p$	U	B	B	B	B	U
$L^q$	U	B	B	B	B	U
$L^{\infty }$, i	U	B	B	B	B	U
$L^{\infty }$, c	U	U	U	U	U	U

In the Table 1, “$L^1$, i” corresponds to $H^1$, and “$L^{\infty }$, i” corresponds to the norm closure of the Haar system in $SL^{\infty }$.

#### Proof of Theorem 2.10

Given $Z = Z(\varvec{\sigma },X,Y)\in \mathcal{H}\mathcal{H}(\delta ^2)$, we distinguish between the following 6 cases:

1. $\Vert \cdot \Vert _X\sim \Vert \cdot \Vert _{L^1}$, $(\sigma _I^{(1)})$ is constant, and
  - $$(\sigma _J^{(2)})$$ is independent;
  - $(\sigma _J^{(2)})$ is constant and $\Vert \cdot \Vert _Y\not \sim \Vert \cdot \Vert _{L^{\infty }}$.
2. $\Vert \cdot \Vert _Y\sim \Vert \cdot \Vert _{L^1}$, $(\sigma _J^{(2)})$ is constant, and
  - $$(\sigma _I^{(1)})$$ is independent;
  - $(\sigma _I^{(1)})$ is constant and $\Vert \cdot \Vert _X\not \sim \Vert \cdot \Vert _{L^{\infty }}$.
3. $\Vert \cdot \Vert _X\sim \Vert \cdot \Vert _{L^\infty }$ and $(\sigma _I^{(1)})$ is constant;
4. $\Vert \cdot \Vert _Y\sim \Vert \cdot \Vert _{L^\infty }$ and $(\sigma _J^{(2)})$ is constant.

Since the proof of Case (2) (a) and Case (2) b are almost identical to the proofs of Case (1) (a) and Case (1) (b) (swapping the functions *f* and *g* defined below), we will omit them. The situation for the proofs of Case (3) and Case (4) is similar.

#### Case (1), (a) and (b)

We put $$I_k = J_k = [0,2^{-k})$$, $$k\in \mathbb {N}_0$$ and define our test functions

2.6 $$\begin{aligned} f = \sum _{k=0}^{n} |I_k|^{-1} h_{I_k} = |I_{n+1}|^{-1} \chi _{I_{n+1}} - \chi _{[0,1)}, \qquad \text {and}\qquad g = \sum _{l=0}^{n} a_l r_l, \end{aligned}$$

where $r_l = \sum _{J\in \mathcal {D}_l} k_J$ and $(a_k)_{k=0}^n$ is a finite sequence of scalars. On the one hand, we note that

2.7 $$\begin{aligned} \Vert f\otimes g\Vert _Z \sim \left\| \left| \sum _{k=0}^{n} |I_k|^{-1} h_{I_k} \right| \right\| _{L^1} \cdot \left\| {{\,\mathrm{\mathbb {E}}\,}}\left| \sum _{l=0}^n a_l \sum _{J\in \mathcal {D}_l} \sigma _J^{(2)} k_J \right| \right\| _Y \le 2\cdot \left\| {{\,\mathrm{\mathbb {E}}\,}}\left| \sum _{l=0}^n a_l \sum _{J\in \mathcal {D}_l} \sigma _J^{(2)} k_J \right| \right\| _Y, \end{aligned}$$

while the other hand, using $\Vert \cdot \Vert _Y\ge \Vert \cdot \Vert _{L^1}$ (see Proposition 2.7 (i)) and Khintchine’s inequality yields

$$\begin{aligned} \Vert \mathcal {C} f\otimes g\Vert _Z&\sim \left\| s\mapsto \left\| t\mapsto {{\,\mathrm{\mathbb {E}}\,}}\left| \sum _{l=0}^{n} a_l \sum _{J\in \mathcal {D}_l} \sigma _J^{(2)} k_J(t) \sum _{k=l}^{n} |I_k|^{-1} h_{I_k}(s) \right| \right\| _Y \right\| _{L^1}\\&\ge \left\| s\mapsto {{\,\mathrm{\mathbb {E}}\,}}\int _{0}^{1} \left| \sum _{l=0}^{n} a_l \sum _{J\in \mathcal {D}_l} \sigma _J^{(2)} k_J(t) \sum _{k=l}^{n} |I_k|^{-1} h_{I_k}(s) \right| \textrm{d} t \right\| _{L^1}\\&= \left\| s\mapsto {{\,\mathrm{\mathbb {E}}\,}}\int _{0}^{1} \left| \sum _{l=0}^{n} a_l r_l(t) \sum _{k=l}^{n} |I_k|^{-1} h_{I_k}(s) \right| \textrm{d} t \right\| _{L^1} \\&\quad \sim \left\| \left( \sum _{l=0}^{n} a_l^2 \left( \sum _{k=l}^{n} |I_k|^{-1} h_{I_k}\right) ^{2} \right) ^{1/2} \right\| _{L^1}\\&= \left\| \left( \sum _{l=0}^n a_l^2 \left( |I_{n+1}|^{-1} \chi _{I_{n+1}} - |I_l|^{-1} \chi _{I_l}\right) ^2 \right) ^{1/2} \right\| _{L^1}. \end{aligned}$$

Using that $\bigl ||I_{n+1}|^{-1} \chi _{I_{n+1}} - |I_l|^{-1} \chi _{I_l}\bigr | \ge |I_l|^{-1} \chi _{I_l^{-}}$ and the disjointness of the intervals $I_l^-$, $l\in \mathbb {N}_0$, we can further estimate

$$\begin{aligned}&\Vert \mathcal {C} f\otimes g\Vert _Z \gtrsim \left\| s\mapsto \left( \sum _{l=0}^{n} a_l^2 |I_l|^{-2} \chi _{I_l^-}(s) \right) ^{1/2} \right\| _{L^1}\\&\quad = \left\| s\mapsto \sum _{l=0}^{n} |a_l| |I_l|^{-1} \chi _{I_l^-}(s) \right\| _{L^1} = \frac{1}{2} \sum _{l=0}^{n} |a_l|. \end{aligned}$$

Suppose that $\mathcal {C}$ is bounded then the latter estimate together with (2.7) yields

2.8 $$\begin{aligned} \left\| {{\,\mathrm{\mathbb {E}}\,}}\left| \sum _{l=0}^n a_l \sum _{J\in \mathcal {D}_l} \sigma _J^{(2)} k_J \right| \right\| _Y \gtrsim \sum _{l=0}^{n} |a_l|, \end{aligned}$$

for all finite sequences of scalars $(a_l)_{l=0}^n$. If $(\sigma _J^{(2)})$ is independent as per Case (1) (a), then (2.8) is cannot hold since

$$\begin{aligned} {{\,\mathrm{\mathbb {E}}\,}}\left| \sum _{l=0}^n a_l \sum _{J\in \mathcal {D}_l} \sigma _J^{(2)} k_J \right| \le \left( \sum _{l=0}^{n} a_l^2\right) ^{1/2}. \end{aligned}$$

On the other hand, if $(\sigma _J^{(2)})$ is constant according to Case (1) (b), then (2.8) yields

$$\begin{aligned} \left\| \sum _{l=0}^n a_l r_l \right\| _Y \gtrsim \sum _{l=0}^{n} |a_l|, \end{aligned}$$

which is only possible if $\Vert \cdot \Vert _Y\sim \Vert \cdot \Vert _{L^{\infty }}$ (see the proof of [43, Proposition 2.c.10]).

#### Case (3)

In this case, our test functions are not simple tensor products, but of the following form:

$$\begin{aligned} z = \sum _{k=1}^{n} (h_{I_{2k}} - h_{I_{2k-1}})\otimes \sum _{J\in \mathcal {D}_{2k}} k_J = \sum _{k=1}^{n} (h_{I_{2k}} - h_{I_{2k-1}})\otimes r_{2k} \end{aligned}$$

where $$I_k = [0,2^{-k})$$, $$k\in \mathbb {N}_0$$. First, observe that the functions $$h_{I_{2k}}- h_{I_{2k-1}}$$, $$k\in \mathbb {N}$$ are disjointly supported, and hence,

2.9 $$\begin{aligned} \begin{aligned} \Vert z\Vert _Z&\sim \mathop {\mathrm {ess\,sup}}\limits _s \left\| t\mapsto {{\,\mathrm{\mathbb {E}}\,}}\left| \sum _{k=1}^{n} \bigl ( h_{I_{2k}}(s) - h_{I_{2k-1}}(s) \bigr ) \sum _{J\in \mathcal {D}_{2k}} \sigma _J^{(2)} k_J(t) \right| \right\| _Y\\&= \max _{1\le k\le n} \bigl | h_{I_{2k}}(s) - h_{I_{2k-1}}(s)\bigr | \le 2. \end{aligned} \end{aligned}$$

On the other hand, since $\Vert \cdot \Vert _Y\ge \Vert \cdot \Vert _{L^1}$ (see Proposition 2.7 (i)), we obtain

$$\begin{aligned} \Vert \mathcal {C} z\Vert _Z&\sim \mathop {\mathrm {ess\,sup}}\limits _s \left\| t\mapsto \left| \sum _{k=1}^{n} h_{I_{2k}}(s) r_{2k}(t) \right| \right\| _Y \ge \left\| t\mapsto \left| \sum _{k=1}^{n} h_{I_{2k}}(0) r_{2k}(t) \right| \right\| _Y\\&\ge \int _{0}^{1} \left| \sum _{k=1}^{n} r_{2k}(t) \right| \textrm{d} t \sim \sqrt{n}, \end{aligned}$$

which in view of (2.9) proves that $\mathcal {C}$ is unbounded. $\square$

A Haar system Hardy space $Z(\varvec{\sigma },X,Y)$ can be identified isometrically with a subspace of $X(Y(L^1(\varvec{\sigma })))$. This representation is useful in studying semi-stable Haar multipliers on *Z* that we define later in Sect. 2.3.2

#### Definition 2.11

Let $$X,Y\in \mathcal {H}(\delta )$$, $$\varvec{\sigma }\in \varvec{\Sigma }$$, and $$Z = Z(\varvec{\sigma },X,Y)$$.

- Define $Z^\Omega = X(Y(L^1(\varvec{\sigma })))$ as the completion of
  $$\begin{aligned} \mathcal {V}_1(\delta )\otimes \mathcal {V}_1(\delta )\otimes L^1(\varvec{\sigma }) = \langle \{f\otimes g\otimes a: f,g\in \mathcal {V}_1(\delta ), a\in L^1(\varvec{\sigma })\} \rangle \end{aligned}$$
  with the norm
  $$\begin{aligned} \left\| \sum _{j=1}^nf_j\otimes g_j\otimes a_j \right\| _{Z^\Omega }&= \left\| {{\,\mathrm{\mathbb {E}}\,}}\left| \sum _{j=1}^nf_j\otimes g_j\otimes a_j(\varvec{\sigma }) \right| \right\| _{X(Y)}\\&= \left\| s\mapsto \left\| t\mapsto {{\,\mathrm{\mathbb {E}}\,}}\left| \sum _{j=1}^nf_j(s)g_j(t)a_j(\varvec{\sigma }) \right| \right\| _Y \right\| _X. \end{aligned}$$
- Define the linear map $\mathcal {O}:Z\rightarrow Z^\Omega$ given by
  $$\begin{aligned} z = \sum _{(I,J)\in \mathcal {D}} a_{I,J} h_I\otimes k_J \mapsto \sum _{(I,J)\in \mathcal {D}} a_{I,J} h_I\otimes k_J\otimes \big (\sigma ^{(1)}_I\otimes \sigma ^{(2)}_J\big ), \end{aligned}$$
  where, for each $I_0,J_0\in \mathcal {D}$, we denote by $\sigma _{I_0}^{(1)}\otimes \sigma _{J_0}^{(2)}:\varvec{\sigma }\rightarrow \{-1,1\}$ the function given by $\big ((\sigma _I^{(1)})_{I\in \mathcal {D}},(\sigma ^{(2)}_J)_{J\in \mathcal {D}}\big )\mapsto \sigma _{I_0}^{(1)}\sigma _{J_0}^{(2)}$.

Note that for $f\in X$, $g\in Y$, and $a\in L^1(\varvec{\sigma })$ we have $\Vert f\otimes g\otimes a\Vert _{Z^\Omega } = \Vert f\Vert _X\Vert g\Vert _Y\Vert a\Vert _{L^1}$. Therefore, for every $w\in \mathcal {V}_1(\delta )\otimes \mathcal {V}_1(\delta )\otimes L^1(\varvec{\sigma })$ the quantity $\Vert w\Vert _{Z^\Omega }$ is well defined. By the definition of *Z*, the operator $\mathcal {O}:Z\rightarrow Z^\Omega$ is an into isometry.

#### Remark 2.12

Let $Z = Z(\varvec{\sigma },X,Y)\in \mathcal{H}\mathcal{H}(\delta ^2)$ and $m\in L^\infty ([0,1)^2\times \varvec{\sigma })$ such that, for all $w\in Z^\Omega$, the pointwise product *mw* is in $Z^\Omega$. We define the pointwise multiplier $M:Z^\Omega \rightarrow Z^\Omega$ by putting

$$\begin{aligned} (M w)(s,t,\sigma ) = m(s,t,\sigma )\cdot w(s,t,\sigma ), \qquad s,t\in [0,1),\;\sigma \in \varvec{\sigma }. \end{aligned}$$

By the monotonicity of the Haar system spaces *X*, *Y* (see Proposition 2.7 (v)), we obtain that *M* is well-defined and $\Vert M\Vert _{\mathcal {L}(Z^\Omega )}\le \Vert m\Vert _{L^\infty }$.

For context, we point out two facts that we will not use.

- $$\Vert M\Vert _{\mathcal {L}(Z^\Omega )} = \Vert m\Vert _{L^\infty }$$.
- Given $m\in L^\infty ([0,1)^2\times \varvec{\sigma })$ and if neither the *X* norm nor the *Y* norm is equivalent to the $\Vert \cdot \Vert _\infty$-norm then, automatically, for all $w\in Z^\Omega$, $mw\in Z^\Omega$.

The main theorem splits, in fact, into two version for a space *Z* in $\mathcal{H}\mathcal{H}(\delta ^2)$ depending on whether a certain idempotent Haar multiplier, the Capon projection, extends to a bounded linear operator on *Z*.

#### Definition 2.13

The *Capon projection* is the bi-parameter Haar multiplier $\mathcal {C}:\mathcal {V}(\delta ^2)\rightarrow \mathcal {V}(\delta ^2)$ given by

$$\begin{aligned} \mathcal {C}(h_I\otimes k_J) = \left\{ \begin{array}{ll} h_I\otimes k_J &{}\quad \text{ if } I\in \mathcal {D}_i,\;J\in \mathcal {D}_j\text { and } i\ge j, \\ 0 &{}\quad \text{ otherwise. } \end{array} \right. \end{aligned}$$

That is, $\mathcal {C}$ is a projection onto the space spanned by the “lower triangular” part of the bi-parameter Haar system.

The main result we prove for Haar system Hardy spaces is the following. It is a direct consequence of Theorems 2.32 and 2.40

#### Theorem 2.14

Let $Z \in \mathcal{H}\mathcal{H}(\delta ^2)$ and let $\textrm{HM}(Z)$ denote the set of bounded Haar multipliers on *Z*.

(i) If $\mathcal {C}$ is unbounded on *Z* then $\textrm{HM}(Z)$ approximately 1-projectionally reduces to the class of scalar operators. In particular, $\textrm{HM}(Z)$ has the *C*-primary factorization property, for every $C>2$.
(ii) If $\mathcal {C}$ is bounded on *Z* then $\textrm{HM}(Z)$ approximately 1-projectionally reduces to the two-dimensional subalgebra $\{\lambda \mathcal {C}+\mu ({{\,\textrm{Id}\,}}-\mathcal {C}):\lambda ,\mu \in \mathbb {R}\}$.

From Theorems 2.10 and 2.14 we can deduce Theorem 2.3 about $L^1(L^p)$, $L^p(L^1)$, $L^1(H^1)$, and $H^1(L^1)$.

We would like to point to Arazy’s work on the primarity of tensor products of certain symmetric sequence spaces, including trace class and Schatten class spaces. Using the boundedness of the Kwapień–Pełczyński main triangular projection (see [33]) on the Schatten *p*-class, $1<p<\infty$, he obtained the primarity of those spaces in [2]. Subsequently he exploited the unboundedness of the main triangular projection on the trace class to obtain that even $S^1$ is primary in [3].

### Stabilization of bi-parameter Haar multipliers

In the remainder of this section, we will describe the process behind obtaining Theorem 2.14. This process includes reducing and solving one-parametric problems, and thus, we include some results in this setting.

#### One-parametric stabilization

We express the stability of a coefficient sequence $(d_I)$ over the dyadic tree $\mathcal {D}$ in terms of the following variational semi-norm, which is similar in spirit to the characterization by Semenov and Uksusov [58] of the bounded Haar multipliers on $L^1$.

##### Notation 2.15

For a Haar multiplier $D:\mathcal {V}(\delta )\rightarrow \mathcal {V}(\delta )$, whose coefficients are $(d_I)$ define

$$\begin{aligned} \Vert D\Vert _\infty = \sup _{I\in \mathcal {D}} |d_I| \qquad \text {and}\qquad \Vert D\Vert _\textrm{T} = \sum _{I\in \mathcal {D}} \left( |d_I - d_{I^+}| + |d_I - d_{I^-}| \right) + |d_{[0,1)}|. \end{aligned}$$

If $\Vert D\Vert _\infty < \infty$ we call *D* $\ell ^{\infty }$*-bounded* and and denote the space of all $\ell ^\infty$ bounded Haar multipliers by $HM^{\infty }(\delta )$. We call $\Vert D\Vert _{\textrm{T}}$ the tree variation norm of *D* and if $\Vert D\Vert _{\textrm{T}} < \infty$ we call *D* tree-stable.

For a non-principal ultrafilter $\mathcal {U}$ on $\mathbb {N}$ let $\lambda _\mathcal {U}$ denote the norm-one linear functional on the space of all $\ell ^\infty$-bounded Haar multipliers *D*

$$\begin{aligned} \lambda _\mathcal {U}(D) = \lim _{i\rightarrow \mathcal {U}}\sum _{I\in \mathcal {D}_i}|I|d_I = \lim _{i\rightarrow \mathcal {U}} \left\langle \sum _{I\in \mathcal {D}_i} h_I, D\left( \sum _{I\in \mathcal {D}_i}h_I \right) \right\rangle . \end{aligned}$$

Note that $$\lambda _\mathcal {U}({{\,\textrm{Id}\,}}) = 1$$.

In Remark 2.17 below, we elaborate on the connection between $\lambda _\mathcal {U}$ and a local version of the inner product expression of $\lambda _\mathcal {U}$ used by Enflo via Maurey in [45].

##### Definition 2.16

A sequence $\tilde{H} = (\tilde{h}_I)_{I\in \mathcal {D}}$ in $\mathcal {V}(\delta )$ is called a *faithful Haar system* if the following conditions are satisfied.

- For each $I\in \mathcal {D}$ there is a finite family $\mathcal {A}_I\subset \mathcal {D}$ of pairwise disjoint members of $\mathcal {D}$ and $(\varepsilon _K: K\in \mathcal {A}_I)\in \{\pm 1\}^{\mathcal {A}_I}$ such that
  $$\begin{aligned} \tilde{h}_{I} = \sum _{K\in \mathcal {A}_I} \varepsilon _K h_K. \end{aligned}$$
- If for all $I\in \mathcal {D}$ we put $\Gamma _I = \cup \{K:K\in \mathcal {A}_I\}$ then $\Gamma _{[0,1)} = [0,1)$ and
  $$\begin{aligned} \Gamma _{I^+} = \{\tilde{h}_I = 1\} \quad \text {and}\quad \Gamma _{I^-} = \{\tilde{h}_I = -1\}. \end{aligned}$$

If, in addition, there exists a strictly increasing sequence $(m_i)_{i=0}^\infty$ in $\mathbb {N}_0$ such that

(c) for all $i\in \mathbb {N}_0$ and $I\in \mathcal {D}_i$, $\mathcal {A}_I\subset \mathcal {D}_{m_i}$,

then we call $(\tilde{h}_I)_{I\in \mathcal {D}}$ a *faithful Haar system relative to the frequencies *$(m_i)_{i=0}^\infty$.

##### Remark 2.17

1. The following observation can be shown by induction. Let $(\tilde{h}_I)_{I\in \mathcal {D}} \subset \mathcal {V}(\delta )$ be a faithful Haar system. Then for any $(a_I:I\in \mathcal {D})\in c_{00}(\mathcal {D})$, the functions $\sum _{I\in \mathcal {D}} a_I h_I$ and $\sum _{I\in \mathcal {D}} a_I \tilde{h}_I$ have the same distribution.
2. A collection $(\tilde{h}_I)_{I\in \mathcal {D}}$ is a faithful Haar system relative to frequencies $(m_i)_{i=0}^\infty$ if and only if ${{\,\textrm{supp}\,}}(\tilde{h}_{[0,1)}) = [0,1)$ and for each $i\in \mathbb {N}_0$ and $I\in \mathcal {D}_{i}$ the following two conditions hold.
  - The vector $\tilde{h}_I$ is a linear combination of $(h_J)_{J\in \mathcal {D}_{m_i}}$ with coefficients in $\{-1,0,1\}$.
  - For $$\varepsilon \in \{\pm 1\}$$, $${{\,\textrm{supp}\,}}(\tilde{h}_{I^\varepsilon }) = \{\tilde{h}_{I} = \varepsilon \}$$.

Faithful Haar systems will be used in the sequel to capture a stabilized behavior of a Haar multiplied *D* on $\mathcal {V}(\delta )$ which, under certain assumptions on a norm on $\mathcal {V}(\delta )$, leads to a factorization of a “simple” operator through *D*. More precisely, we will only use faithful Haar systems relative to some frequencies $(m_i)_{i=0}^\infty$. The additional restriction (c) in their definition allows a more precise result: a diagonal operator *D*, under the right assumptions, can be expressed as an approximate projectional factor of the scalar operator $\lambda _\mathcal {U}(D) I$, i.e., the scalar $\lambda _\mathcal {U}(D)$ is independent of the accuracy of the approximation.

##### Notation 2.18

Let $D:\mathcal {V}(\delta )\rightarrow \mathcal {V}(\delta )$ be an $\ell ^{\infty }$-bounded Haar multiplier, and $\tilde{H} = (\tilde{h}_I)_{I\in \mathcal {D}}$ be a faithful Haar system relative to the frequencies $(m_i)_{i=0}^\infty \subset \mathbb {N}$.

Define the linear maps $B = B_{\tilde{H}}:\mathcal {V}(\delta )\rightarrow \mathcal {V}(\delta )$, and $A = A_{\tilde{H}}:\mathcal {V}(\delta )\rightarrow \mathcal {V}(\delta )$ by

$$\begin{aligned} Af&= \sum _{I\in \mathcal {D}}\frac{ h_I}{|I|} \langle \tilde{h}_I, f\rangle , \quad \text {and}\quad Bf = \sum _{I\in \mathcal {D}} \left\langle \frac{h_I}{|I|},f\right\rangle \tilde{h}_I, \quad \text {for } f \in \mathcal {V}(\delta ), \end{aligned}$$

and put $\tilde{D} = D|_{\tilde{H}} = A\circ D\circ B:\mathcal {V}(\delta )\rightarrow \mathcal {V}(\delta )$. Observe that *AB* is the identity map, and hence *BA* is a projection onto the linear span of $\tilde{h}_I$, $I\in \mathcal D$. Actually, since $(\tilde{h}_I)$ is a faithful Haar system, *BA* is the conditional expectation with respect to the $\sigma$-algebra generated by $\tilde{h}_I$, $I\in \mathcal D$.

As it will be proved later (see Proposition 3.1), for *D* and $\tilde{H}$ as above, $D|_{\tilde{H}}$ is a diagonal operator with entries that come from averaging entries of *D*. The theorem below is a restatement of Theorem 3.7.

##### Theorem 2.19

Let $D:\mathcal {V}(\delta )\rightarrow \mathcal {V}(\delta )$ be an $\ell ^\infty$-bounded Haar multiplier. Then, for every non-principal ultrafilter and $\eta >0$ there exists a faithful Haar system relative to some frequencies $(m_i)_{i=0}^\infty$ such that

$$\begin{aligned} \left\| D|_{\tilde{H}} - \lambda _\mathcal {U}(D){{\,\textrm{Id}\,}}\right\| _{\textrm{T}} < \eta \end{aligned}$$

and $$\lambda _\mathcal {U}(D) = \lambda _\mathcal {U}(D|_{\tilde{H}})$$.

The following Assumption 2.20 and Theorem 2.21 are not used in our paper; however, they serve as a stepping stone to a later introduction of an analogous, less obvious assumption in two parameters

##### Assumption 2.20

Assume that *X* is the completion of $\mathcal {V}(\delta )$ under a norm $\Vert \cdot \Vert$ such that the following are satisfied.

(i) There exists $C>0$ such that for every faithful Haar system $\tilde{H}$ relative to some frequencies $(m_i)_{i=0}^\infty$ we have
  $$\begin{aligned} \Vert A_{\tilde{H}}\Vert _{\mathcal {L}(X)}\Vert B_{\tilde{H}}\Vert _{\mathcal {L}(X)} \le C, \end{aligned}$$
  where for a linear $T:\mathcal {V}(\delta )\rightarrow \mathcal {V}(\delta )$, $\Vert T\Vert _{\mathcal {L}(X)} = \sup \{\Vert Tf\Vert : \Vert f\Vert \le 1\}$.
(ii) There exist a constant $\beta >0$ such that for every Haar multiplier $D:\mathcal {V}(\delta )\rightarrow \mathcal {V}(\delta )$ we have
  $$\begin{aligned} \Vert D\Vert _{\mathcal {L}(X)} \le \beta \Vert D\Vert _{\textrm{T}}. \end{aligned}$$

In [40, Lemma 4.5 and Proposition 7.1] the first named author and Thomas Speckhofer showed that the spaces in $\mathcal {H}\mathcal {H}(\delta )$ satisfy Assumption 2.20. A direct application of Theorem 2.19 yields the following Theorem 2.21 (see also [40, Theorem 3.6]).

##### Theorem 2.21

Let *X* satisfy Assumption 2.20 for some constants $\beta$, and *C*. Then, for every $\eta >0$ and a non-principal ultrafilter $\mathcal {U}$ on $\mathbb {N}$ every bounded Haar multiplier $D:X\rightarrow X$ is a *C*-projectional factor of $\lambda _\mathcal {U}(D){{\,\textrm{Id}\,}}$ with error $\eta$.

If additionally $\lambda _\mathcal {U}(D)\ne 0$, then for every $C'>C$, *D* is a $C'$-factor of $\lambda _\mathcal {U}(D){{\,\textrm{Id}\,}}$.

##### Remark 2.22

As previously mentioned, the a priori knowledge of the constant $$\lambda _\mathcal {U}(D)$$ is achieved using faithful Haar systems relative to some frequencies. This can be seen as a parallel approach to the one by Enflo via Maurey in [45], where they proved that every bounded linear operator on $$L_p$$, $$1\le p<\infty $$, is, for every $$\eta >0$$, a projectional factor with error $$\eta $$ of a pointwise multiplier $$M_g$$ and *g* does not depend on the error $$\eta $$. To do this, they considered local versions of the functional $$\lambda _\mathcal {U}$$, each defined for a measurable subset of the unit interval of positive measure, that collectively gave rise to a measure. The derivative of this measure gave the pointwise multiplier in question. In Haar system Hardy spaces bounded functions don’t necessarily define bounded pointwise multipliers and, thus, the reduction of Haar multipliers to scalar operators is more appropriate in this setting. Note here that pointwise multipliers preserving $$H^ 1 $$ or its dual space are characterized by Stegenga [59], and the resulting conditions are quite restrictive. See also Chapter VI in Garnett [20]. An explicit translation of the Stegenga condition to the martingale setting appears, for instance in the paper by Nakai and Sadasue [54].

#### Two-parametric setting

##### Notation 2.23

For Haar multiplier *D* on $\mathcal {V}(\delta _2)$ with coefficients $(d_{I,J})$ we put $\Vert D\Vert _\infty = \sup _{I,J\in \mathcal {D}} |d_{I,J}|$, and let $\text {HM}^\infty (\delta ^2)$ be the space of $\Vert \cdot \Vert _\infty$-bounded Haar multipliers on $\mathcal {V}(\delta ^2)$.

**Fig. 1:**
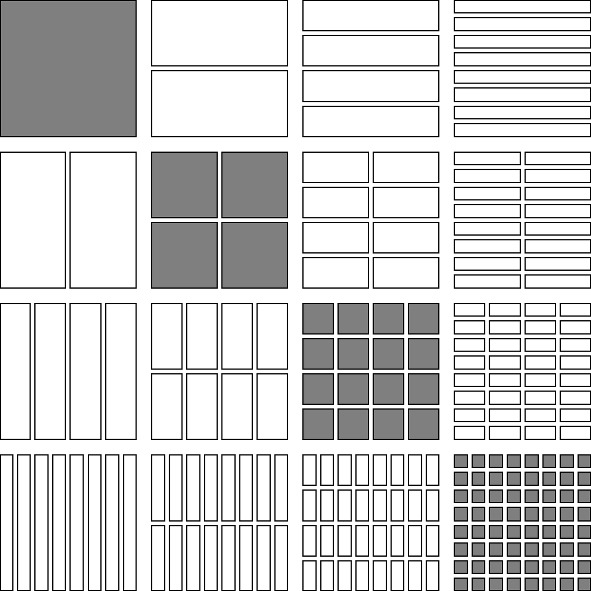
Diagonal, “squares”

**Fig. 2:**
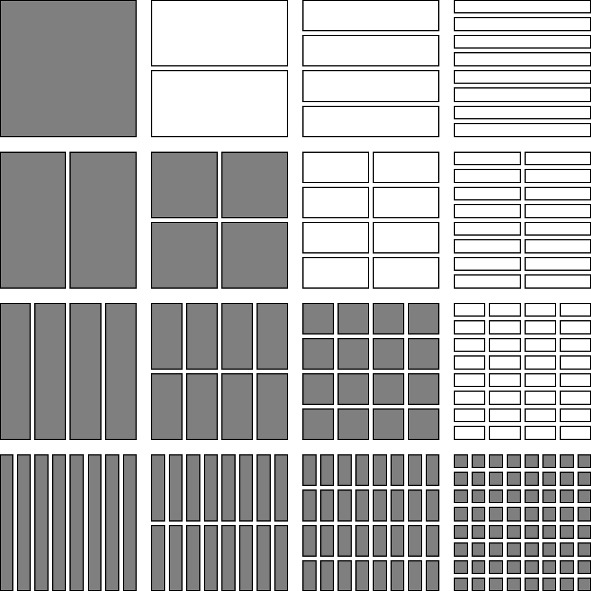
Lower triangular, “tall rectangles and squares”

**Fig. 3:**
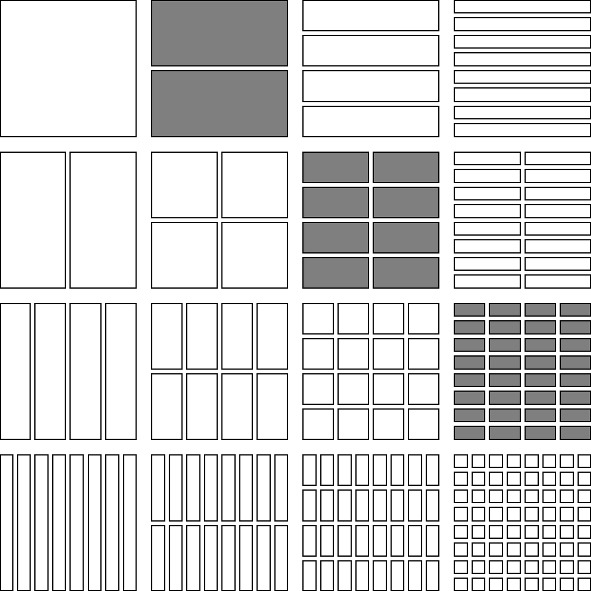
Superdiagonal, “2 : 1 aspect ratio rectangles”

**Fig. 4:**
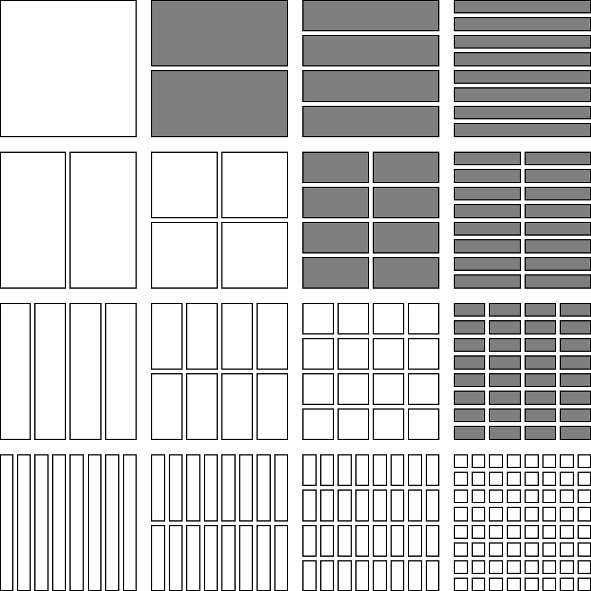
Upper triangular, “wide rectangles”

We list four special subsets of $\mathcal {D}\times \mathcal {D}$ as follows (see Figs. 1, 2, 3, 4).

- The *lower triangular part of* $\mathcal {D}\times \mathcal {D}$ is the subset $\cup _{j=0}^\infty \cup _{i=j}^\infty \mathcal {D}_i\times \mathcal {D}_j$.
- The *upper triangular part of* $\mathcal {D}\times \mathcal {D}$ is the subset $\cup _{i=0}^\infty \cup _{j=i+1}^\infty \mathcal {D}_i\times \mathcal {D}_j$, further subdivided into
  - *left upper triangular part* $\cup _{i=1}^\infty \cup _{j=i+1}^\infty \mathcal {D}_i\times \{J\in \mathcal {D}_j: J\subset [0,1/2)\}$ and the
  - *right upper triangular part* $\cup _{i=1}^\infty \cup _{j=i+1}^\infty \mathcal {D}_i\times \{J\in \mathcal {D}_j: J\subset [1/2,1)\}$.
- The *diagonal part of* $\mathcal {D}\times \mathcal {D}$ is the subset $\cup _{i=0}^\infty \mathcal {D}_i\times \mathcal {D}_i$.
- The *superdiagonal part of* $\mathcal {D}\times \mathcal {D}$ is the subset $\cup _{i=0}^\infty \mathcal {D}_{i}\times \mathcal {D}_{i+1}$.

Based on these subsets of $\mathcal {D}\times \mathcal {D}$ we define the *bi-tree variation semi-norm* on a subspace of $\textrm{HM}(\delta ^2)$ by letting for $D\in \textrm{HM}(\delta ^2)$

$$\begin{aligned} \Vert D\Vert _\mathrm {T^2S}&= \sum _{k=0}^{\infty } (k+1) \max _{\begin{array}{c} I,J\in \mathcal {D}_k\\ \omega ,\xi \in \{\pm 1\} \end{array}} |d_{I,J} - d_{I^{\omega },J^{\xi }}| + \sum _{k=0}^{\infty } (k+1) \max _{\begin{array}{c} I\in \mathcal {D}_k,\\ J\in \mathcal {D}_{k+1}\\ \omega ,\xi \in \{\pm 1\} \end{array}} |d_{I,J} - d_{I^{\omega },J^{\xi }}|\\&\qquad + \sum _{i=0}^{\infty } \sum _{j=0}^i \max _{\begin{array}{c} I\in \mathcal {D}_i\\ J\in \mathcal {D}_j\\ \omega \in \{\pm 1\} \end{array}} |d_{I,J} - d_{I^{\omega },J}| + \sum _{j=0}^{\infty } \sum _{i=0}^j \max _{\begin{array}{c} I\in \mathcal {D}_i\\ J\in \mathcal {D}_{j+1}\\ \xi \in \{\pm 1\} \end{array}} |d_{I,J} - d_{I,J^{\xi }}|. \end{aligned}$$

Adding the roots $$d_{[0,1),[0,1)}$$, $$d_{[0,1),[0,1/2)}$$ and $$d_{[0,1),[1/2,1)}$$ to $$\Vert \cdot \Vert _{T^2S}$$ yields the *bi-tree variation norm* $$\Vert D\Vert _{\textrm{T}^2}$$ given by

$$\begin{aligned} \Vert D\Vert _{\textrm{T}^2} = \Vert D\Vert _{\textrm{T}^2} + |d_{[0,1),[0,1)}| + |d_{[0,1),[0,1/2)}| + |d_{[0,1),[1/2,1)}|. \end{aligned}$$

If $\Vert D\Vert _{\textrm{T}^2S}<\infty$ we call *D* *bi-tree semi-stabilized* and we denote by $\textrm{HM}^{\textrm{T}^2}(\delta ^2)$ the vector space of all bi-tree semi-stabilized Haar multipliers on $\mathcal {V}(\delta ^2)$.

For a non-principal ultrafilter $\mathcal {U}$ let $\lambda _\mathcal {U}$, $\mu _\mathcal {U}$ denote the norm-one linear functionals on $\textrm{HM}^\infty (\delta ^2)$.

$$\begin{aligned} \lambda _\mathcal {U}(D)&= \lim _{j\rightarrow \mathcal {U}} \lim _{i\rightarrow \mathcal {U}} \langle r_i\otimes r_j, D r_i\otimes r_j \rangle = \lim _{j\rightarrow \mathcal {U}} \lim _{i\rightarrow \mathcal {U}} \sum _{I\in \mathcal {D}_i}\sum _{J\in \mathcal {D}_j}|I||J|d_{I,J}\\ \mu _\mathcal {U}(D)&= \lim _{i\rightarrow \mathcal {U}} \lim _{j\rightarrow \mathcal {U}} \langle r_i\otimes r_j, D r_i\otimes r_j \rangle = \lim _{i\rightarrow \mathcal {U}} \lim _{j\rightarrow \mathcal {U}} \sum _{I\in \mathcal {D}_i}\sum _{J\in \mathcal {D}_j}|I||J|d_{I,J}. \end{aligned}$$

Note that $$\lambda _\mathcal {U}(I) = \mu _\mathcal {U}(I) = 1$$.

##### Remark 2.24

A telescoping argument (see, e.g., the proof of Proposition 3.22) easily yields that, for $D\in \textrm{HM}^\infty (\delta ^2)$ with entries $(d_{I,J})_{I,J\in \mathcal {D}\times \mathcal {D}}$ and a non-principal ultrafilter $\mathcal {U}$ on $\mathbb {N}$,

$$\begin{aligned} \left| \lambda _\mathcal {U}(D) - d_{[0,1)[0,1)}\right| + \left| \mu _\mathcal {U}(D) - \frac{1}{2}\left( d_{[0,1),[0,1/2)} + d_{[0,1),[1/2,1)}\right) \right| \le \Vert D\Vert _{\textrm{T}^2}. \end{aligned}$$

##### Remark 2.25

The kernel of $\Vert \cdot \Vert _{\textrm{T}^2S}$ is the three-dimensional subspace of $\in \textrm{HM}^{\textrm{T}^2}(\delta ^2)$ consisting of all *D* with constant entries $\lambda$ on the lower triangular part, $\mu _1$ on the left upper triangular part, and $\mu _2$ on the right upper triangular part of $\mathcal {D}\times \mathcal {D}$. Although this object is implicitly approximated in our proofs, we are ultimately interested in its two-dimensional subspace $\{\lambda \mathcal {C}+\mu ({{\,\textrm{Id}\,}}-\mathcal {C}):\lambda ,\mu \in \mathbb {R}\}$, i.e., the subspace of all *D* in $\textrm{ker}(\Vert \cdot \Vert _{\textrm{T}^2S})$ for which $\mu _1 = \mu _2 = \mu$ (see Theorem 2.29 below).

##### Definition 2.26

Let $\tilde{H}=(\tilde{h}_I)_{I\in \mathcal {D}}$ and $\tilde{K}=( \tilde{k}_J)_{J\in \mathcal {D}}$ be faithful Haar systems relative to the frequencies $(m_i)$ and $(n_j)$, respectively. We call $\tilde{H}\otimes \tilde{K}=(\tilde{h}_I\otimes \tilde{k}_J: I,J\in \mathcal {D})$ a 2-parameter faithful Haar systems relative to the frequencies $(m_i)_{i=0}^\infty$ and $(n_j)_{j=0}^\infty$.

##### Notation 2.27

If $S,T:\mathcal {V}(\delta )\rightarrow \mathcal {V}(\delta )$ are linear operators we define $S\otimes T:\mathcal {V}(\delta ^2)\rightarrow \mathcal {V}(\delta ^2)$ given by

$$\begin{aligned} S\otimes T\left( \sum _{I,J\in \mathcal {D}} a_{I,J} h_I \otimes k_J\right) = \sum _{I,J\in \mathcal {D}} a_{I,J} S(h_I)\otimes T(k_J). \end{aligned}$$

##### Notation 2.28

Let $\tilde{H}\otimes \tilde{K}= (\tilde{h}_I\otimes \tilde{k}_J: I,J\in \mathcal {D})$ be a faithful Haar system relative to the frequencies $(m_i)$ and $(n_j)$, and let $D:\mathcal {V}(\delta ^2)\rightarrow \mathcal {V}(\delta ^2)$ be a Haar multiplier. Define

$$\begin{aligned} A&= A_{\tilde{H}\otimes \tilde{K}} = A_{\tilde{H}}\otimes A_{ \tilde{K}} :\mathcal {V}(\delta ^2)\rightarrow \mathcal {V}(\delta ^2), \quad Af = \sum _{I,J\in \mathcal {D}} \frac{h_I \otimes k_J}{|I|\cdot |J|}\langle \tilde{h}_I\otimes \tilde{k}_J , f\rangle \\ B&= B_{\tilde{H}\otimes \tilde{K}} = B_{\tilde{H}}\otimes B_{ \tilde{K}}:\mathcal {V}(\delta ^2)\rightarrow \mathcal {V}(\delta ^2), \quad Bf = \sum _{I,J\in \mathcal {D}} \left\langle \frac{h_I\otimes k_J}{|I|\cdot |J|},f\right\rangle \tilde{h}_I\otimes \tilde{k}_J,\\ \tilde{D}&= D|_{\tilde{H}\otimes \tilde{K}} = A \circ D \circ B. \end{aligned}$$

Observe that *AB* is the identity map.

As we will later see in Proposition 3.14, $D|_{\tilde{H}\otimes \tilde{K}}$ is a Haar multiplier with entries that come from averaging those of *D*.

##### Theorem 2.29

Let $D:\mathcal {V}(\delta ^2)\rightarrow \mathcal {V}(\delta ^2)$ be an $\ell ^\infty$-bounded Haar multiplier. Then, for every non-principal ultrafilter $\mathcal {U}$ on $\mathbb {N}$ and $\eta >0$ there exists a bi-parameter faithful Haar system $\tilde{H}\otimes \tilde{K}$ relative to some frequencies $(m_i)_{i=0}^\infty$ and $(n_j)_{j=0}^\infty$ such that

$$\begin{aligned} \left\| D|_{\tilde{H}\otimes \tilde{K}} - \left( \lambda _\mathcal {U}(D)\mathcal {C}+ \mu _\mathcal {U}(D)(I-\mathcal {C})\right) \right\| _{\textrm{T}^2} < \eta \end{aligned}$$

and $$\lambda _\mathcal {U}(D) = \lambda _\mathcal {U}(D|_{\tilde{H}\otimes \tilde{K}})$$, $$\mu _\mathcal {U}(D) = \mu _\mathcal {U}(D|_{\tilde{H}\otimes \tilde{K}})$$.

Theorem 2.29 will be proved in Sect. 3 where we also restate it in Theorem 3.20. But we will first state conditions under which Theorem 2.29 is directly applicable to the completion *Z* of $\mathcal {V}(\delta ^2)$ under an appropriate norm. As we will see eventually, every bi-parameter Haar system Hardy space satisfies these conditions.

##### Definition 2.30

Let *Z* be the completion of $\mathcal {V}(\delta ^2)$ under a norm $\Vert \cdot \Vert$ such that the following are satisfied.

- There exists $C>0$ such that for every bi-parameter Haar system $\tilde{H}\otimes \tilde{K}$ relative to some frequencies $(m_i)_{i=0}^\infty$ and $(n_j)_{j=0}^\infty$ we have
  $$\begin{aligned} \Vert A_{\tilde{H}\otimes \tilde{K}}\Vert _{\mathcal {L}(X)}\Vert B_{\tilde{H}\otimes \tilde{K}}\Vert _{\mathcal {L}(X)} \le C, \end{aligned}$$
  where for a linear $T:\mathcal {V}(\delta ^2)\rightarrow \mathcal {V}(\delta ^2)$, $\Vert T\Vert _{\mathcal {L}(X)} = \sup \{\Vert Tf\Vert : \Vert f\Vert \le 1\}$.
- There exists a constant $\beta >0$ such that for every bounded Haar multiplier $D:Z\rightarrow Z$ and a non-principal ultrafilter $\mathcal {U}$ on $\mathbb {N}$
  $$\begin{aligned} \left\| D - \left( \lambda _\mathcal {U}(D)\mathcal {C} + \mu _\mathcal {U}(D)(I-\mathcal {C})\right) \right\| _{\mathcal {L}(Z)} \le \beta \Vert D\Vert _{\mathrm {T^2}}. \end{aligned}$$

Then, we say that *Z* satisfies the *Capon property* or that *Z* is a *Capon space*. If, additionally, we wish to be particular about the constant *C* for which *Z* satisfies a, then we call *Z* a *C*-Capon space.

##### Remark 2.31

Let us explain the content of condition b. Importantly, in it, we assume that $D:Z\rightarrow Z$ is already bounded.

1. If we assume that, on *Z*, $\mathcal {C}$ is bounded, then it is not very hard to see that b is equivalent to the following simpler condition.
  (c’) There exists $\beta '>0$ such that for every bounded Haar multiplier $D:Z\rightarrow Z$,
    $$\begin{aligned} \Vert D\Vert _{\mathcal {L}(Z)} \le \beta '\Vert D\Vert {_{\mathrm {T^2}}}. \end{aligned}$$
2. If we assume that $\mathcal {C}$ is unbounded on *Z*, then b is equivalent to the following.
  (c”) There exists $\beta ''>0$ such that for every bounded Haar multiplier $D:Z\rightarrow Z$ and non-principal ultrafilter $\mathcal {U}$ on $\mathbb {N}$
    $$\begin{aligned} \Vert D\Vert _{\mathcal {L}(Z)} \le \beta ''\Vert D\Vert _{\mathrm {T^2}}\quad \text {and}\quad \lambda _\mathcal {U}(D) = \mu _\mathcal {U}(D). \end{aligned}$$

The following theorem is an immediate application of Theorem 2.29, Definition 2.30, and, when $\mathcal {C}$ is unbounded, Remark 2.31.

##### Theorem 2.32

Let *Z* be a *C*-Capon space.

(i) For every $\eta >0$ and non-principal ultrafilter $\mathcal {U}$ on $\mathbb {N}$ every bounded Haar multiplier $D:X\rightarrow X$ is a *C*-projectional factor of $\lambda _\mathcal {U}(D)\mathcal {C} + \mu _\mathcal {U}(D)({{\,\textrm{Id}\,}}-\mathcal {C})$ with error $\eta$.
(ii) If, additionally, $\mathcal {C}$ is unbounded on *Z* then $\lambda _\mathcal {U}(D) = \mu _\mathcal {U}(D)$ and, thus, for every $\eta >0$, *D* is a *C*-projectional factor with error $\eta$ of $\lambda _\mathcal {U}(D){{\,\textrm{Id}\,}}$.

If, in particular, $D:Z\rightarrow Z$ is a bounded Haar multiplier such that, for some non-principal ultrafilter $\mathcal {U}$ on $\mathbb {N}$, $(\lambda _\mathcal {U}(D),\mu _\mathcal {U}(D))\notin \{(1,0),(0,1)\}$ then the identity on *Z* factors through *D* or ${{\,\textrm{Id}\,}}-D$.

### Proving that Haar system Hardy spaces are Capon spaces

In Sect. 4, we prove that a Haar system Hardy space *Z* satisfies property Definition 2.30 a for $C=1$ (Theorem 4.9). Although these are delicate proofs, they follow from a direct analysis of the Haar system Hardy space norm. In contrast, the proof of property b involves pointwise multipliers on the space $Z^\Omega$ and their proximity to Haar multipliers. This theory is developed in Sect. 5, which also serves as a bridge between the pointwise multiplier approach (introduced by Enflo via Maurey [45], and heavily used by Capon [10, 11, 14] or Enflo and Starbird [18], and the Haar multiplier approach based on Gamlen Gaudet [19] and the work of Alspach, Enflo, and Odell [1] which was extensively used by Bourgain, Rosenthal and Schechtman [6], Bourgain [5], Johnson, Maurey, Schechtman and Tzafriri [28], Lindenstrauss and Tzafriri [43] and the authors of the present paper [34, 35, 37–39, 48, 51].

#### Notation 2.33

To each $D\in \textrm{HM}^{\mathrm {T^2}}(\delta ^2)$ we assign sequences of functions $m_{1,k}^D,m_{2,k}^D:[0,1)^2\rightarrow \mathbb {R}$, $k\in \mathbb {N}$, as follows:

$$\begin{aligned} m_{1,k}^D = \sum _{(I,J)\in \mathcal {D}_k\times \mathcal {D}_k} d_{I,J} \chi _{I\times J} \quad \text {and}\quad m_{2,k}^D = \sum _{(I,J)\in \mathcal {D}_k\times \mathcal {D}_{k+1}} d_{I,J} \chi _{I\times J}. \end{aligned}$$

#### Remark 2.34

Clearly, for each $k\in \mathbb {N}$, $m_{1,k}^D,m_{2,k}^D$ satisfy the assumption of Remark 2.12. Therefore, they give rise to pointwise multipliers $M_{1,k}^D,M_{2,k}^D:Z^\Omega \rightarrow Z^\Omega$ defined as

$$\begin{aligned} (M_{i,k}^D w)(s,t,\sigma ) = m_{i,k}^D(s,t) w(s,t,\sigma ), \qquad s,t\in [0,1),\;\sigma \in \varvec{\sigma }, \end{aligned}$$

for $i=1,2$ and $k\in \mathbb {N}$, and hence, $\Vert M_{i,k}^D\Vert \le \Vert m_{i,k}^D\Vert _{L^{\infty }} \le \Vert D\Vert _{\infty }\le \Vert D\Vert _{\mathrm {T^2}}$, $i=1,2$.

#### Remark 2.35

For each $D\in \textrm{HM}^{\mathrm {T^2}}(\delta ^2)$ and $i=1,2$, by the variational nature of $\Vert \cdot \Vert _{\mathrm {T^2}}$, a telescoping argument (see, e.g., the proof of Proposition 3.22) yields

$$\begin{aligned} \lim _{n\rightarrow \infty }\sup _{n\le k,\ell } |m_{i,k}^D(s,t) - m_{i,\ell }^D(s,t)| = 0, \end{aligned}$$

uniformly in $s,t\in [0,1)$. Define $m_1^D,m_2^D\in L^\infty [0,1)^2$ with

2.10 $$\begin{aligned} m_1^D(s,t) = \lim _{k\rightarrow \infty } d_{I_k(s),J_k(t)} \qquad \text {and}\qquad m_2^D(s,t) = \lim _{k\rightarrow \infty } d_{I_k(s),J_{k+1}(t)}, \end{aligned}$$

where, for $k\in \mathbb {N}$, $I_k(s)$ and $J_k(t)$ are the unique dyadic intervals in $\mathcal {D}_k$ such that $s\in I_k(s)$ and $t\in J_k(t)$. Then $\lim _k\Vert m_{1,k}^D-m_{1}^D\Vert _{L^\infty } = 0$ and $\lim _k\Vert m_{2,k}^D-m_{2}^D\Vert _{L^\infty } = 0$ and, in particular, the sequences $(M^D_{1,k})_{k=1}^\infty$ $(M^D_{2,k})_{k=1}^\infty$ converge in the operator norm of $\mathcal {L}(Z^\Omega )$.

We conclude that $D\in \textrm{HM}^{\mathrm {T^2}}(\delta ^2)$ gives rise, for $i=1,2$, to the pointwise multipliers $M_i^D:Z^{\Omega }\rightarrow Z^\Omega$ defined as

2.11 $$\begin{aligned} (M_i^D w)(s,t,\sigma ) = m_i^D(s,t) w(s,t,\sigma ), \qquad s,t\in [0,1),\;\sigma \in \varvec{\sigma }. \end{aligned}$$

such that $$\Vert M_i^D\Vert \le \Vert m_i^D\Vert _{L^{\infty }} \le \Vert D\Vert _{\infty }\le \Vert D\Vert _{\mathrm {T^2}}$$.

#### Remark 2.36

For $D\in \textrm{HM}^{\mathrm {T^2}}(\delta ^2)$, the definition of $\Vert \cdot \Vert _{\mathrm {T^2}\textrm{S}}$ easily yields

2.12 $$\begin{aligned} |d_{[0,1),[0,1)} - m_1^D(s,t)| \le \Vert D\Vert _{\mathrm {T^2}\textrm{S}} \quad \text { and}\quad |d_{[0,1),J_1(t)} - m_2^D(s,t)| \le \Vert D\Vert _{\mathrm {T^2}\textrm{S}}. \end{aligned}$$

#### Remark 2.37

We don’t use the following properties but mention them for context.

(i) For any Haar system Hardy space *Z* and $i=1,2$, the assignment $D\mapsto M_i^D$ is a norm-one linear operator from $\textrm{HM}^{\mathrm {T^2}}(\delta ^2)$ to $\mathcal {L}(Z^\Omega )$.
(ii) For $D\in \textrm{HM}^{\mathrm {T^2}}(\delta ^2)$, the functions $m_1^D, m_2^D$ are continuous on the set $([0,1)\setminus \mathbb {D})^2$, where $\mathbb {D}$ denotes the dyadic rationals in [0, 1].

Recall, for a Haar system Hardy space *Z* there is a linear isometry $\mathcal {O}:Z\rightarrow Z^\Omega$ given in Definition 2.11 and, for $D\in \textrm{HM}^{\mathrm {T^2}}(\delta ^2)$, we can define the linear operators $\mathcal {O}D,M^D_1\mathcal {O},M^D_2\mathcal {O}:\mathcal {V}(\delta ^2)\rightarrow Z^\Omega$. Denote $\mathcal {V}_Z = (\mathcal {V}(\delta ^2),\Vert \cdot \Vert _Z)$.

The following is included in the first main result of Sect. 5, Theorem 5.1 and Corollary 5.2.

#### Theorem 2.38

Let $Z = Z(\varvec{\sigma },X,Y)$ be a Haar system Hardy space. For every $\ell ^\infty$-bounded Haar multiplier $D:\mathcal {V}(\delta ^2) \rightarrow \mathcal {V}(\delta ^2)$ the following estimates hold.

(i) $$\Vert (\mathcal {O}D-M_1^D\mathcal {O})\mathcal {C}\Vert _{\mathcal {L}(\mathcal {V}_Z,Z^\Omega )} \le 4\Vert D\Vert _{\mathrm {T^2}\textrm{S}}$$ and $$\Vert (\mathcal {O}D-M_2^D\mathcal {O})(I-\mathcal {C})\Vert _{\mathcal {L}(\mathcal {V}_Z,Z^\Omega )} \le 4 \Vert D\Vert _{\mathrm {T^2}\textrm{S}}$$.
(ii) $$\Vert (M_1^D - M_2^D)\mathcal {O}\mathcal {C}\Vert _{\mathcal {L}(\mathcal {V}_Z,Z^\Omega )} \le \Vert D\Vert _{\mathcal {L}(Z)} + \Vert D\Vert _\infty + 8 \Vert D\Vert _{\mathrm {T^2}\textrm{S}}$$.

Furthermore, $\mathcal {C}$ is bounded on *Z* if and only if there exist a non-principal ultrafilter $\mathcal {U}$ on $\mathbb {N}$ and a bounded Haar multiplier $D:Z\rightarrow Z$ such that $\lambda _\mathcal {U}(D)\ne \mu _\mathcal {U}(D)$.

For the space *Z* on which the Capon projection is unbounded, we require an additional statement that may be of independent interest. It is proof is given in Theorem 5.3

#### Theorem 2.39

Let $Z = Z(\varvec{\sigma },X,Y)$ be a Haar system Hardy space on which the Capon projection $\mathcal {C}$ is unbounded. If $m\in L^\infty [0,1)^2$ is such that, for the induced pointwise multiplier *M*, $M\mathcal{O}\mathcal{C}:(\mathcal {V}(\delta ^2),\Vert \cdot \Vert _Z)\rightarrow Z^\Omega$ is bounded, then $M = 0$.

With the above two theorems at hand, we easily verify property b of Definition 2.30 for Haar system Hardy spaces.

#### Theorem 2.40

Every Haar system Hardy space $Z = Z(\varvec{\sigma },X,Y)$ is a 1-Capon space.

#### Proof

For *Z* with bounded Capon operator property b of Definition 2.30 follows almost immediately. Indeed, for a bounded Haar multiplier $D:Z\rightarrow Z$, we verify property (c’) of Remark 2.31.

$$\begin{aligned} \Vert D\Vert _{\mathcal {L}(Z)}&\le \Vert D\mathcal {C}\Vert _{\mathcal {L}(Z)} + \Vert D(I-\mathcal {C})\Vert _{\mathcal {L}(Z)} \le 8\Vert D\Vert _{\mathrm {T^2}} + (\Vert m_1^D\Vert _{L^\infty }+\Vert m_2^D\Vert _{L^\infty })\cdot \Vert \mathcal {C}\Vert _{\mathcal {L}(Z)}\\&\le (8+\Vert \mathcal {C}\Vert _{\mathcal {L}(Z)})\cdot \Vert D\Vert _{\mathrm {T^2}}. \end{aligned}$$

Next assume that the Capon operator is unbounded on *Z*. We proceed to verify property (c”) of Remark 2.31. For a bounded Haar multiplier $D:Z\rightarrow Z$, by Theorem 2.38, we have $\lambda _\mathcal {U}(D) = \mu _\mathcal {U}(D)$. To prove the remainder of (c”), we assume that $\Vert D\Vert _{\mathrm {T^2}} < \infty$. By Theorems 2.38 (ii) and 2.39, $M_1^D = M_2^D$ and, thus, by Theorem 2.38 (i), $\Vert \mathcal {O}D - M_1^D\mathcal {O}\Vert _{\mathcal {V}_Z,Z^\Omega } \le 8\Vert D\Vert _{\mathrm {T^2}\textrm{S}}$. By Remark 2.24 and Remark 2.36 we have $\Vert m_1^D - \lambda _\mathcal {U}(D)\Vert _{L^\infty } \le 2\Vert D\Vert _{\mathrm {T^2}\textrm{S}}$. Therefore, $\Vert D\Vert _{\mathcal {L}(Z)} \le |\lambda _\mathcal {U}(D)| + 10 \Vert D\Vert _{\mathrm {T^2}\textrm{S}}\le 11 \Vert D\Vert _{\textrm{T}^2}$.$\square$

The following theorem exhibits an important connection between the pointwise multiplier function $m_1^D$ and $\lambda _{\mathcal {U}}(D)$ respectively $m_2^D$ and $\mu _{\mathcal {U}}(D)$. See also Corollary 5.2.

#### Theorem 2.41

Let $D\in \textrm{HM}^{\mathrm {T^2}}(\delta ^2)$. Then the functions $m_1^D, m_2^D:[0,1)^2\rightarrow \mathbb {R}$ given by (2.10) satisfy, for any non-principal ultrafilter $\mathcal {U}$ on $\mathbb {N}$,

$$\begin{aligned} \lambda _{\mathcal {U}}(D) = \int _{0}^{1} \int _{0}^{1} m_1^D(s,t) \textrm{d} t \textrm{d} s \qquad \text {and}\qquad \mu _{\mathcal {U}}(D) = \int _{0}^{1} \int _{0}^{1} m_2^D(s,t) \textrm{d} t \textrm{d} s. \end{aligned}$$

#### Proof

We will only verify the identities for $\lambda _{\mathcal {U}}(D)$, the other identity follow by similar arguments. First, recall that $m_1^D(s,t) = \lim _k d_{I_k(s),J_k(t)}$ and $|m_1^D(s,t)|\le \Vert D\Vert _{\infty } < \infty$ for all $s,t\in [0,1)$. Next, let $j\le i$, $J\in \mathcal {D}_j$ and $I,L\in \mathcal {D}_i$ be fixed, let $K_k,L_k\in \mathcal {D}_k$ be such that $K_j\supset K_{j+1}\supset \dots K_i = I$ and $J = L_j\supset L_{j+1}\supset \dots L_i = L$. Thus,

$$\begin{aligned} |d_{I,J} - d_{K_j,J}|&\le \sum _{k=j}^{i-1} |d_{K_{k+1},J} - d_{K_k,L}| \le \sum _{k=j}^{\infty } \max _{\begin{array}{c} K\in \mathcal {D}_k\\ M\in \mathcal {D}_j\\ \omega \in \{\pm 1\} \end{array}} |d_{K^{\omega },M} - d_{K,M}|,\\ |d_{K_j,J} - d_{I,L}|&\le \sum _{k=j}^{i-1} |d_{K_k,L_k} - d_{K_{k+1},L_{k+1}}| \le \sum _{k=j}^{\infty } \max _{\begin{array}{c} K,M\in \mathcal {D}_k\\ \omega ,\xi \in \{\pm 1\} \end{array}} |d_{K,M} - d_{K^{\omega },M^{\xi }}|. \end{aligned}$$

Adding the above inequalities yields

$$\begin{aligned} |d_{I,J} - d_{I,L}| \le \sum _{k=j}^{\infty } \left( \max _{\begin{array}{c} K\in \mathcal {D}_k\\ M\in \mathcal {D}_j\\ \omega \in \{\pm 1\} \end{array}} |d_{K^{\omega },M} - d_{K,M}| + \max _{\begin{array}{c} K,M\in \mathcal {D}_k\\ \omega ,\xi \in \{\pm 1\} \end{array}} |d_{K,M} - d_{K^{\omega },M^{\xi }}| \right) . \end{aligned}$$

We use the latter estimate and obtain

$$\begin{aligned}&\left| \sum _{\begin{array}{c} I\in \mathcal {D}_i\\ J\in \mathcal {D}_j \end{array}} |I| |J| d_{I,J} - \sum _{I,L\in \mathcal {D}_i} |I| |L| d_{I,L} \right| = \left| \sum _{I\in \mathcal {D}_i} |I| \left( \sum _{J\in \mathcal {D}_j} |J| d_{I,J} - \sum _{L\in \mathcal {D}_i} |L| d_{I,L} \right) \right| \\&\qquad = \left| \sum _{I\in \mathcal {D}_i} |I| \left( \sum _{J\in \mathcal {D}_j} \sum _{\begin{array}{c} L\in \mathcal {D}_i\\ L\subset J \end{array}} |L| (d_{I,J} - d_{I,L}) \right) \right| \le \sum _{I\in \mathcal {D}_i} |I| \sum _{J\in \mathcal {D}_j} \sum _{\begin{array}{c} L\in \mathcal {D}_i\\ L\subset J \end{array}} |L| |d_{I,J} - d_{I,L}|\\&\qquad \le \sum _{I\in \mathcal {D}_i} |I| \sum _{J\in \mathcal {D}_j} \sum _{\begin{array}{c} L\in \mathcal {D}_i\\ L\subset J \end{array}} |L| \sum _{k=j}^{\infty } \left( \max _{\begin{array}{c} K\in \mathcal {D}_k\\ M\in \mathcal {D}_j\\ \omega \in \{\pm 1\} \end{array}} |d_{K^{\omega },M} - d_{K,M}| + \max _{\begin{array}{c} K,M\in \mathcal {D}_k\\ \omega ,\xi \in \{\pm 1\} \end{array}} |d_{K,M} - d_{K^{\omega },M^{\xi }}| \right) \\&\qquad = \sum _{k=j}^{\infty } \left( \max _{\begin{array}{c} K\in \mathcal {D}_k\\ M\in \mathcal {D}_j\\ \omega \in \{\pm 1\} \end{array}} |d_{K^{\omega },M} - d_{K,M}| + \max _{\begin{array}{c} K,M\in \mathcal {D}_k\\ \omega ,\xi \in \{\pm 1\} \end{array}} |d_{K,M} - d_{K^{\omega },M^{\xi }}| \right) . \end{aligned}$$

Since $\Vert D\Vert _{\mathrm {T^2}\textrm{S}} < \infty$, the latter expression tends to 0 if $j\rightarrow \infty$, thus,

$$\begin{aligned} 0&= \lim _{j\rightarrow \mathcal {U}} \lim _{i\rightarrow \mathcal {U}} \left| \sum _{\begin{array}{c} I\in \mathcal {D}_i\\ J\in \mathcal {D}_j \end{array}} |I| |J| d_{I,J} - \sum _{I,L\in \mathcal {D}_i} |I| |L| d_{I,L} \right| = \left| \lambda _{\mathcal {U}}(D) - \lim _{i\rightarrow \mathcal {U}} \sum _{I,L\in \mathcal {D}_i} |I| |L| d_{I,L} \right| \\&= \left| \lambda _{\mathcal {U}}(D) - \lim _{k\rightarrow \infty } \int _{0}^{1} \int _{0}^{1} d_{I_k(s),J_k(t)} \textrm{d} t \textrm{d} s \right| = \left| \lambda _{\mathcal {U}}(D) - \int _{0}^{1} \int _{0}^{1} m_1^D(s,t) \textrm{d} t \textrm{d} s \right| \end{aligned}$$

as claimed.$$\square $$

### Conclusions and open problems

Theorem 2.14, our main theorem on the class of Haar multipliers $\textrm{HM}(Z)$ acting on a Haar system Hardy space $Z\in \mathcal{H}\mathcal{H}(\delta ^2)$, gives the following factorization results:

(i) If Capon’s projection $\mathcal {C}$ is unbounded on *Z* then $\textrm{HM}(Z)$ approximately 1-projectionally reduces to the class of scalar operators, and hence, $\textrm{HM}(Z)$ has the *C*-primary factorization property, for every $C>2$.
(ii) If $\mathcal {C}$ is bounded on *Z* then $\textrm{HM}(Z)$ approximately 1-projectionally reduces to the two-dimensional subalgebra $\{\lambda \mathcal {C}+\mu ({{\,\textrm{Id}\,}}-\mathcal {C}):\lambda ,\mu \in \mathbb {R}\}$.

We now list questions and problems arising in connection with our current work.

#### Question 2.42

Let $Z = Z(\varvec{\sigma },X,Y)$ be a Haar system Hardy space.

1. Under which conditions on *Z* is it true that *Z* has the *C*-primary factorization property, for some $C < \infty$? More specifically, when does the class of operators $\mathcal {L} (Z)$ approximately projectionally reduce to the class of scalar multiples of the identity?
2. In view of our main result, Theorem 2.14, we ask under which conditions on *Z* is it true that every bounded linear operator on *Z* approximately projectionally reduces to a Haar multiplier in *Z*?

If a space has the primary factorization property, and if Pełczyński’s decomposition method is applicable, then the space *Z* is primary. This leads to our next set of questions.

#### Question 2.43

Let $Z = Z(\varvec{\sigma },X,Y)$ be a Haar system Hardy space.

1. Under which conditions is *Z* and/or its dual space primary?
2. Specifically, find a way to make Pełczyński’s decomposition work in the context of Haar system Hardy spaces. Find a suitable substitute for the accordion property.
3. As a special case of the above problem: Find all primary spaces in the family
  $$\begin{aligned} \mathcal {F} = \{ L^p(L^q),\ H^p (H^q),\ L^p(H^q),\ H^p(L^q) : 1 \le p,q < \infty \}. \end{aligned}$$

Capon’s projection plays a pivotal role in our factorization theorems. We suggest to investigate it more closely. With the following problems we intend to find intrinsic, and easily verifiable conditions for its boundedness on bi-parameter Haar system Hardy spaces.

#### Question 2.44

Let $Z = Z(\varvec{\sigma },X,Y)$ be a Haar system Hardy space.

1. Is it true that $\mathcal {C}$ is bounded on *Z* if and only if the bi-parameter Haar system $(h_I\otimes h_J)_{I,J\in \mathcal {D}}$ is unconditional in *Z*?
2. Is it true that $\mathcal {C}$ is bounded on *Z* if and only if *Z* has an unconditional basis?
3. Is it true that *Z* has an unconditional basis if and only if the bi-parameter Haar system is unconditional in *Z*?

Fix $$Z = L^p(L^q) $$ for $$1< p,q < \infty $$. In 1982 Capon [13] proved that the two dimensional algebra of bounded operators $$\{\lambda \mathcal {C}+ \mu ({{\,\textrm{Id}\,}}-\mathcal {C}): \lambda ,\mu \in \mathbb {R}\}$$ acting on *Z* approximately projectionally reduces to $$\{\mu {{\,\textrm{Id}\,}}_Z: \mu \in \mathbb {R}\}$$. In 1994, the third named author [49] observed that Capon’s method applies to the space $$Z = H^1(H^1)$$. This gives rise to the next question:

#### Question 2.45

Assume that the Capon projection $\mathcal {C}$ is bounded on a Haar system Hardy space $Z = Z(\varvec{\sigma },X,Y)$.

1. Does the two dimensional sub-algebra $\{\lambda \mathcal {C}+ \mu ({{\,\textrm{Id}\,}}-\mathcal {C}): \lambda ,\mu \in \mathbb {R}\}$ (of operators on *Z*) approximately projectionally reduce to $\{\mu {{\,\textrm{Id}\,}}_Z: \mu \in \mathbb {R}\}$?
2. What about the special cases when $Z = L^p(H^1)$ or $Z = H^1(L^p )$, $1< p < \infty$?

We next point to several papers in which related questions are addressed.

In 1974, Casazza and Lin [16] proved: If a Banach space *E* has a sub-symmetric basis $(e_j)_{j=1}^{\infty }$, then *E* satisfies the primary factorization property. This led them to ask if every Banach space *E* with a symmetric basis is primary. To this date, the problem is open. Let $(e_j^{*})_{j=1}^{\infty }$ denote the biorthogonal functionals of the sub-symmetric basis $(e_j)_{j=1}^{\infty }$. Assuming that $(e_j^{*})_{j=1}^{\infty }$ is “non-$\ell ^1$-splicing”, the first named author showed that $E^{*}$ satisfies the primary factorization property (see [36]). In view of this result, we ask:

#### Question 2.46

Is $E^{*}$ a primary space, whenever the biorthogonal functionals $(e_j^{*})_{j=1}^{\infty }$ are “non-$\ell ^1$-splicing”?

In 1977 Casazza [15] obtained the, by now classical, result that the James space *J* is primary. We point out that Casazza’s proof [15] circumvented the accordion property.

The James space *J* is the earliest example of a space admitting a conditional spreading basis. It is not true that every space with such a basis is primary (see, e.g., [4, page 1235]). The subclass of equal signs additive (ESA) bases was introduced by Brunel and Sucheston in 1976 [9], and its isomorphically invariant version of convex block homogeneous bases was introduced and studied in [4]. There it was shown that every space with a conditional spreading basis is isomorphic to the direct sum of a space with an ESA basis and a space with an unconditional finite dimensional Schauder decomposition (UFDD). Spaces *Z* with an ESA basis are natural candidates for being primary and the authors of [4] attempted to prove this. Arguments from [4] yield that such *Z* has the primary factorization property. As usual, attaining primariness stumbles on the lack of an accordion-type property for certain complemented subspaces with a UFDD with a type of distributional subsymmetry.

As part of the pursuit of new methods to show primariness, we reiterate here a problem from [4, page 1235]:

#### Question 2.47

Is every Banach space with an ESA basis primary?

## Coefficient Stabilization

In this section, we prove Theorem 2.29, i.e., that every $\ell ^\infty$-bounded Haar multiplier $D:\mathcal {V}(\delta ^2) \rightarrow \mathcal {V}(\delta ^2)$ can be reduced to one with bounded bi-tree variation norm that is, in fact, close to a linear combination of $\mathcal {C}$ and ${{\,\textrm{Id}\,}}-\mathcal {C}$.

### One-parameter faithful Haar systems

We first develop the necessary algebraic and probabilistic language in the one-parameter setting. Thereafter we derive its two-parametric counterpart.

#### Proposition 3.1

Let $D:\mathcal {V}(\delta ) \rightarrow \mathcal {V}(\delta )$ be an $\ell ^{\infty }$-bounded diagonal operator, $\tilde{H} = (\tilde{h}_I)_{I\in \mathcal {D}}$ be a faithful Haar system relative to the frequencies $(m_i)_{i=0}^\infty$, and let $\tilde{D} = D|_{\tilde{H}}:\mathcal {V}(\delta ) \rightarrow \mathcal {V}(\delta )$ be defined as in Notation 2.18. Then $\tilde{D}$ is a diagonal operator whose coefficients are

3.1 $$\begin{aligned} \tilde{d}_I&= \sum _{\begin{array}{c} K\in \mathcal {D}_{m_i}\\ K\subset {{\,\textrm{supp}\,}}(\tilde{h}_I) \end{array}} \frac{|K|}{|I|} d_K, \qquad i\ge 0, I\in \mathcal {D}_i. \end{aligned}$$

#### Proof

Suppose that $i\ge 0$ for $I\in \mathcal {D}_i$, the collection $\mathcal {A}_I$ is given by $\mathcal {A}_I = \{ K\in \mathcal {D}_{m_i}: K\subset {{\,\textrm{supp}\,}}(\tilde{h}_I)\}$, and let $(\theta _K)\in \{\pm 1\}^{\mathcal {D}}$. We then define

$$\begin{aligned} \tilde{h}_I = \sum _{K\in \mathcal {A}_I} \theta _{K} h_K. \end{aligned}$$

It follows for $I\ne J$ that

$$\begin{aligned} \langle \tilde{h}_I, D(\tilde{h}_J)\rangle = \left\langle \sum _{K\in \mathcal {A}_I} \theta _{K} h_K, \sum _{K'\in \mathcal {A}_J} \theta _{K'} d_{K'} h_{K'} \right\rangle = 0, \end{aligned}$$

and

$$\begin{aligned} \langle \tilde{h}_I, D(\tilde{h}_I)\rangle = \left\langle \sum _{K\in \mathcal {A}_I} \theta _{K} h_K , \sum _{K'\in \mathcal {A}_I} \theta _{K'} d_{K'} h_{K'} \right\rangle = \sum _{K\in \mathcal {A}_I } d_K |K|. \end{aligned}$$

Thus

$$\begin{aligned} \tilde{D}(h_I)&= (A D)(\tilde{h}_I) = \sum _{J\in \mathcal {D}} \frac{h_J}{|J|} \langle \tilde{h}_J, D\tilde{h}_I \rangle = \frac{h_I}{|I|} \langle \tilde{h}_I, D\tilde{h}_I \rangle \\&= \frac{h_I}{|I|} \left\langle \sum _{K\in \mathcal {A}_I} \theta _{K} h_K, \sum _{K'\in \mathcal {A}_I} \theta _{K'} d_{K'} h_{K'} \right\rangle = h_I \sum _{\begin{array}{c} K\in \mathcal {D}_{m_i},\\ K\subset {{\,\textrm{supp}\,}}(\tilde{h}_I) \end{array}} \frac{|K|}{|I|} d_K. \end{aligned}$$

$$\square $$

#### Notation 3.2

Let $\widetilde{H}^{(1)} = (\tilde{h}_I^{(1)})_{I\in \mathcal {D}}$ be a faithful Haar system relative to frequencies $(n_i)_{i=0}^\infty$ and let $\widetilde{H}^{(2)} = (\tilde{h}^{(2)}_I)_{I\in \mathcal {D}}$ be a faithful Haar system relative to frequencies $(m_i)_{i=0}^\infty$. For each $I\in \mathcal {D}$, define $\tilde{h}^{(3)}_I = \sum _{J\in \mathcal {D}} |J|^{-1}\langle h_J, \tilde{h}^{(2)}_I \rangle \tilde{h}^{(1)}_J$ and denote $\widetilde{H}^{(2)} *\widetilde{H}^{(1)} = (h^{(3)}_I)_{I\in \mathcal {D}}$.

#### Proposition 3.3

Let $\widetilde{H}^{(1)} = (\tilde{h}_I^{(1)})_{I\in \mathcal {D}}$ be a faithful Haar system relative to frequencies $(n_i)_{i=0}^\infty$ and let $\widetilde{H}^{(2)} = (\tilde{h}^{(2)}_I)_{I\in \mathcal {D}}$ be a faithful Haar system relative to frequencies $(m_i)_{i=0}^\infty$. Then, $\widetilde{H}^{(2)}*\widetilde{H}^{(1)}$ is a faithful Haar system relative to the frequencies $(n_{m_i})_{i=0}^\infty$.

#### Proof

We will use Item (2). For $i\in \mathbb {N}_0$ and $I\in \mathcal {D}_i$ write

$$\begin{aligned} \tilde{h}^{(3)}_I = \sum _{\begin{array}{c} J\in \mathcal {D}_{m_i}\\ J\subset {{\,\textrm{supp}\,}}(\tilde{h}^{(2)}_I) \end{array}} \sum _{\begin{array}{c} L\in \mathcal {D}_{n_{m_i}}\\ L\subset {{\,\textrm{supp}\,}}(\tilde{h}^{(1)}_J) \end{array}} \theta ^{(2)}_{J} \theta ^{(1)}_{L} h_L \end{aligned}$$

This yields that $h^{(3)}_I$ is a linear combination of $(h_J)_{J\in \mathcal {D}_{n_{m_i}}}$ with coefficients in $\{-1,0,1\}$. By taking $i=0$ we also easily obtain ${{\,\textrm{supp}\,}}(\tilde{h}^{(3)}_{[0,1)}) = [0,1)$.

Next, consider the maps $B_1, B_2:\mathcal {V}(\delta ) \rightarrow \mathcal {V}(\delta )$ given by $B_1h_I = \tilde{h}_I^{(1)}$ and $B_2h_I = \tilde{h}_I^{(2)}$ which implies that for $B = B_1B_2$, $Bh_I = \tilde{h}^{(3)}_I$. We extend the vector space $\mathcal {V}(\delta )$ by one dimension to $\tilde{\mathcal {V}} = \langle \chi _{[0,1)}\rangle + \mathcal {V}(\delta ) = \langle \{\chi _I:I\in \mathcal {D}\}\rangle$ and extend *B* on $\tilde{\mathcal {V}}$ by putting $B(\chi _{[0,1)}) = \chi _{[0,1)}$. By Item (1) the map *B* preserves distribution and therefore, if $\mathcal {A}(\mathcal {D})$ is the algebra generated by $\mathcal {D}$, there exists a measure preserving $\phi :\mathcal {A}(\mathcal {D})\rightarrow \mathcal {A}(\mathcal {D})$ such that for every $A\in \mathcal {A}(\mathcal {D})$, $B(\chi _A) = \chi _{\phi (A)}$. In particular, for every $f\in \tilde{\mathcal {V}}$ and $a\in \mathbb {R}$ we have $\{Bf = a\} = \phi (\{f = a\})$ and furthermore ${{\,\textrm{supp}\,}}(Bf) = \phi ({{\,\textrm{supp}\,}}(f))$. Therefore, for every $I\in \mathcal {D}$ and $\varepsilon \in \{\pm 1\}$ we have

$$\begin{aligned} {{\,\textrm{supp}\,}}(\tilde{h}^{(3)}_{I^\varepsilon }) = {{\,\textrm{supp}\,}}(Bh_{I^\varepsilon }) = \phi ({{\,\textrm{supp}\,}}(h_{I^\varepsilon })) = \phi (\{h_I = \varepsilon \}) = \{Bh_I = \varepsilon \} = \{\tilde{h}_I^{(3)} = \varepsilon \}. \end{aligned}$$

$$\square $$

The above proof also implies that the set of all faithful Haar systems $\tilde{H}$ relative to some frequencies with the operation “$*$” is a nonabelian monoid. The next corollary’s content is that $(\tilde{H},D)\mapsto D|_{\tilde{H}}$ defines a left monoidal action. Therefore, if *D* and $\widetilde{H}^{(1)},\ldots ,\widetilde{H}^{(n)}$ then

$$\begin{aligned} D{\mathop {\longrightarrow }\limits ^{\widetilde{H}^{(1)}}}\tilde{D}_1{\mathop {\longrightarrow }\limits ^{\widetilde{H}^{(2)}}}\tilde{D}_2{\mathop {\longrightarrow }\limits ^{\widetilde{H}^{(3)}}}\cdots {\mathop {\longrightarrow }\limits ^{\widetilde{H}^{(n)}}}\tilde{D}_n \end{aligned}$$

coincides with

$$\begin{aligned} D {\mathop {}\limits ^{\underrightarrow{\widetilde{H}^{(n)}*\cdots *\widetilde{H}^{(1)}}}}\tilde{D}_n. \end{aligned}$$

In other words, iterating the operation $D{\mathop {\longrightarrow }\limits ^{\tilde{H}}}\tilde{D}$ always yields a diagonal operator associated to the initial *D* and a faithful Haar system relative to some frequencies.

#### Corollary 3.4

Let $D:\mathcal {V}(\delta ) \rightarrow \mathcal {V}(\delta )$ be an $\ell ^{\infty }$-bounded diagonal operator and $\widetilde{H}^{(1)}$, $\widetilde{H}^{(2)}$ be faithful Haar systems relative to frequencies $(n_i)_{i=0}^\infty$ and $(m_i)_{i=0}^\infty$ respectively. Then, we have

$$\begin{aligned} (D|_{\widetilde{H}^{(1)}})|_{\widetilde{H}^{(2)}} = D|_{\widetilde{H}^{(2)} *\widetilde{H}^{(1)}}. \end{aligned}$$

#### Proof

If we associate $B_1,A_1$ to $\widetilde{H}^{(1)}$, $B_2,A_2$ to $\widetilde{H}^{(2)}$, and *B*, *A* to $\widetilde{H}^{(2)} *\widetilde{H}^{(1)}$, it easily follows that $B_1B_2 = B$ and $A_2A_1 = Q$. Therefore,

$$\begin{aligned} D|_{\widetilde{H}^{(2)} *\widetilde{H}^{(1)}} = ADB = A_2(A_1DB_1)B_2 = (D|_{\widetilde{H}^{(1)}})|_{\widetilde{H}^{(2)}}. \end{aligned}$$

$$\square $$

### Tree-stabilized operators in one-parameter

Tree-stabilized diagonal operators have entries, in a strong sense, very close to each other. As pointed out in Remark 3.6 below, such an operator is in the $\ell ^{\infty }$ sense close to a scalar operator, however, as we will see later, in the appropriate setting, this proximity is achievable in operator norm as well. The main result of this section is that for every $\ell ^{\infty }$-bounded diagonal operator $D:\mathcal {V}(\delta )\rightarrow \mathcal {V}(\delta )$ there exists a faithful Haar system $\tilde{H}$ relative to some frequencies $(m_i)_{i=0}^\infty$ such that $D|_{\tilde{H}}$ is tree-stabilized.

#### Definition 3.5

Let $D:\mathcal {V}(\delta )\rightarrow \mathcal {V}(\delta )$ be a diagonal operators whose coefficients are $(d_I: I\in \mathcal {D})$, let $\eta =(\eta _i)_{i=0}^\infty \subset (0,1)$, and let $k_0\in \mathbb {N}_0$, we call *D* $\eta$-*tree-stabilized from* $k_0$ *on* if

$$\begin{aligned} |d_{L}- d_{L^{\omega }}|\le \eta _k, \qquad \text {for all }k\ge k_0, L\in \mathcal {D}_k, and \omega =\pm 1. \end{aligned}$$

If $k_0=0$ we simply say that *D* is $\eta$-*tree-stabilized.*

The notion of eventual tree-stability becomes, as we will see later, relevant in the two-parameter setting.

#### Remark 3.6

Let $D:\mathcal {V}(\delta )\rightarrow \mathcal {V}(\delta )$ be a Haar multiplier and $\eta =(\eta _i)_{i=0}^\infty \subset (0,1)$. If *D* is $\eta$-tree-stabilized and $\sum _{i=0}^\infty 2^{i}\eta _i <\infty$ then

- $${\lambda = \lim _i\sum _{i\in \mathcal {D}_i}|I|d_I}$$ exists and
- $${ \big \Vert D - \lambda {{\,\textrm{Id}\,}}\big \Vert _{\textrm{T}} \le \sum _{i=0}^\infty (2^{i+1}+1)\eta _i\le 3\sum _{i=0}^\infty 2^i\eta _i.}$$

#### Theorem 3.7

Let $D:\mathcal {V}(\delta )\rightarrow \mathcal {V}(\delta )$ be an $\ell ^{\infty }$-bounded operator. Then, for every sequence of positive numbers $\eta =(\eta _{j})_{j=0}^\infty$ and infinite $M\subset \mathbb {N}$ there exists a faithful Haar system $\tilde{H}=(\tilde{h}_I)_{I\in \mathcal {D}}$ relative to frequencies $(m_i)_{i=0}^\infty$ in *M*, such that the diagonal operator

$$\begin{aligned} \tilde{D}= D|_{ \tilde{H}} \quad \text {is }\eta \text {-tree-stabilized.} \end{aligned}$$

In particular, for every non-principal ultrafilter $\mathcal {U}$ on $\mathbb {N}$ we may choose $\tilde{D}$ such that $\lambda _\mathcal {U}(\tilde{D}) = \lambda _\mathcal {U}(D)$ and, thus,

$$\begin{aligned} \Vert \tilde{D} - \lambda _\mathcal {U}(D){{\,\textrm{Id}\,}}\Vert _{\textrm{T}} \le 3 \sum _{i=0}^\infty 2^{i}\eta _i. \end{aligned}$$

The proof of the above goes through iteratively applying the probabilistic Lemma 3.9. In fact, we will prove the following stronger statement, that we also apply in the two-parameter scenario.

#### Proposition 3.8

Consider the following infinite round two-player game between Player (I) and Player (II). Denote $N^{(-1)} = \mathbb {N}_0$. For $k=0,1,2,\ldots$ in round *k*:

1. First, Player (I) chooses $\eta _k>0$, an infinite $M_k\subset N^{(k-1)}$, and a finite collection $\mathcal {F}_k$ of $\ell ^{\infty }$-bounded diagonal operators on $\mathcal {V}(\delta )$.
2. Then, Player (II) chooses an infinite $N^{(k)}\subset M_k$ and, for $n_k = \min (N^{(k)})$, vectors $(\tilde{h}_I)_{I\in \mathcal {D}_k}$ in $\langle \{h_J:J\in \mathcal {D}_{n_k}\}\rangle$.

Then, Player (II) has a winning strategy to force the following outcome: $\tilde{H} = (\tilde{h}_I)_{I\in \mathcal {D}}$ is a faithful Haar system relative to the frequencies $(n_k)_{k=0}^\infty$ and for each $k_0\in \mathbb {N}$ and $D\in \mathcal {F}_{k_0}$, $\tilde{D} = D|_{\tilde{H}}$ is $(\eta _k)_{k=0}^\infty$-tree -stabilized from $k_0$ on.

#### Proof of Theorem 3.7 using Proposition 3.8

Let *D* and $\eta = (\eta _j)_{j=0}^\infty$ be given and let $\lambda = \lambda _\mathcal {U}(D)$, for some non-principal ultrafilter $\mathcal {U}$ on $\mathbb {N}$. Let *M* be an infinite set such that $\lim _{i\in M}\sum _{i\in \mathcal {D}_i}|I|d_I = \lambda$. In the game above, we assume the role of Player (I) and let Player (II) follow their winning strategy. In round *k*, we chose as error the given $\eta _k$ and $\mathcal {F}_{k} = \{D\}$. The specific choice of $M_k$ only matters in the first round, in which we choose $M_0 = M$.

The resulting faithful Haar system $\tilde{H} = (\tilde{h}_I)_{I\in \mathcal {D}}$ is relative to some frequencies $(m_i)_{i=0}^\infty$ with $\{m_i:i\in \mathbb {N}_0\}\subset M$ and $D|_{\tilde{H}}$ is $\eta$-tree-stabilized. By Proposition 3.1$\lim _i\sum _{I\in \mathcal {D}_i}|I|\tilde{d}_I = \lim _i\sum _{I\in \mathcal {D}_{m_i}}|I| d_I = \lambda$ and by Definition 3.5, $\Vert \tilde{D} - \lambda I\Vert _\textrm{T} \le 3 \sum _{i=0}^\infty 2^i\eta _i.$ $\square$

The choice of the appropriate faithful Haar system $\tilde{H}$ is based on the following probabilistic lemma, which was first introduced in [39] (see also [40, Lemma 8.3]).

#### Lemma 3.9

Let $m< n$, $\Gamma \in \sigma (\mathcal {D}_m)$, $(d_I:I\in \mathcal {D}_n, I\subset \Gamma )\subset \mathbb {R}$ and put for $\theta =(\theta _{J}:J\in \mathcal {D}_m, J\subset \Gamma )\subset \{\pm 1\}$

$$\begin{aligned} \Gamma ^\omega (\theta ) = \left\{ \sum _{\begin{array}{c} J\in \mathcal {D}_m\\ J\subset \Gamma \end{array}} \theta _{J} h_J=\omega \right\} , \qquad \text {for}\quad \omega =\pm 1. \end{aligned}$$

Let $\Theta = (\Theta _{J}:J\in \mathcal {D}_m, J\subset \Gamma )$ be a Rademacher distributed family on a probability space $(\Omega ,\Sigma ,{{\,\mathrm{\mathbb {P}}\,}})$, meaning that the family $(\Theta _{J}:J\in \mathcal {D}_m, J\subset \Gamma )$ is independent and

$$\begin{aligned} {{\,\mathrm{\mathbb {P}}\,}}(\Theta _{J}=1) = {{\,\mathrm{\mathbb {P}}\,}}(\Theta _{J}=-1) = \frac{1}{2}, \qquad J\in \mathcal {D}_m,\ J\subset \Gamma . \end{aligned}$$

For $\omega =\pm 1$ define the random variable

$$\begin{aligned} X_\omega = \sum _{\begin{array}{c} I\in \mathcal {D}_n,\\ I\subset \Gamma ^\omega (\Theta ) \end{array}} d_I \frac{|I|}{| \Gamma ^\omega (\Theta )|} = \sum _{\begin{array}{c} I\in \mathcal {D}_n,\\ I\subset \Gamma ^\omega (\Theta ) \end{array}} \frac{|I|}{|\Gamma |/2} d_I. \end{aligned}$$

Then it follows for the expected value of $X_\omega$

3.2 $$\begin{aligned} {{\,\mathrm{\mathbb {E}}\,}}(X_\omega ) = \sum _{\begin{array}{c} I\in \mathcal {D}_n,\\ I\subset \Gamma \end{array}} \frac{|I|}{|\Gamma |} d_I, \end{aligned}$$

and for the variance of $X_\omega$

3.3 $$\begin{aligned} {{\,\mathrm{\mathbb {V}}\,}}(X_\omega )&= {{\,\mathrm{\mathbb {E}}\,}}\left( \left( X_\omega -{{\,\mathrm{\mathbb {E}}\,}}(X_\omega )\right) ^2\right) \le \frac{2^{-m}}{|\Gamma |} \max _{I\in \mathcal {D}_n, I\subset \Gamma }|d_I|^2. \end{aligned}$$

#### Proof

Without loss of generality we assume $\omega =1$. We compute

$$\begin{aligned} {{\,\mathrm{\mathbb {E}}\,}}\left( \sum _{\begin{array}{c} I\in \mathcal {D}_n,\\ I\subset \Gamma ^+(\Theta ) \end{array}} d_I \frac{|I|}{| \Gamma ^+(\Theta )|}\right)&= \!\sum _{\begin{array}{c} J\in \mathcal {D}_m, \\ J\subset \Gamma \end{array}} {{\,\mathrm{\mathbb {E}}\,}}\left( \sum _{\begin{array}{c} I\in \mathcal {D}_n,\\ I\subset J\cap \Gamma ^+(\Theta ) \end{array}} d_I \frac{2|I|}{| \Gamma |}\right) \\&= \!\sum _{\begin{array}{c} J\in \mathcal {D}_m, \\ J\subset \Gamma \end{array}} {{\,\mathrm{\mathbb {E}}\,}}\left( \sum _{\begin{array}{c} I\in \mathcal {D}_n,\\ I\subset J^{\Theta _{J}} \end{array}} d_I \frac{2|I|}{| \Gamma |}\right) =\!\sum _{\begin{array}{c} I\in \mathcal {D}_n,\\ I\subset \Gamma \end{array}} d_I \frac{|I|}{|\Gamma |} , \end{aligned}$$

where the last equality follows from the fact that for $J\in \mathcal {D}_m$, $J\subset \Gamma$, and $I\in \mathcal {D}_n$, with $I\subset J$ we have

$$\begin{aligned} {{\,\mathrm{\mathbb {P}}\,}}(I\subset J\cap \Gamma ^+(\Theta )) = {{\,\mathrm{\mathbb {P}}\,}}(I\subset J^{\Theta _{J}}) = \frac{1}{2}. \end{aligned}$$

Since the family of random variables

$$\begin{aligned} \left( \sum _{\begin{array}{c} I\in \mathcal {D}_n,\\ I\subset J^{\Theta _{J}} \end{array}} d_I \frac{2|I|}{|\Gamma |}: J\in \mathcal {D}_m,\ J \subset \Gamma \right) \end{aligned}$$

is independent, we deduce that

$$\begin{aligned}&{{\,\mathrm{\mathbb {E}}\,}}\left( \sum _{\begin{array}{c} I\in \mathcal {D}_n,\\ I\subset \Gamma ^+(\Theta ) \end{array}} d_I \frac{|I|}{| \Gamma ^+(\Theta )|}- \sum _{\begin{array}{c} I\in \mathcal {D}_n,\\ I\subset \Gamma \end{array}} d_I \frac{|I|}{|\Gamma |}\right) ^2 \\&\quad = \sum _{\begin{array}{c} J\in \mathcal {D}_m,\\ J\subset \Gamma \end{array}} {{\,\mathrm{\mathbb {E}}\,}}\left( \sum _{\begin{array}{c} I\in \mathcal {D}_n,\\ I\subset J^{ \Theta _{J}} \end{array}} d_I \frac{2|I|}{|\Gamma |}- \sum _{\begin{array}{c} I\in \mathcal {D}_n,\\ I\subset J \end{array}} d_I \frac{|I|}{|\Gamma |} \right) ^2\\&\quad = \sum _{\begin{array}{c} J\in \mathcal {D}_m, \\ J\subset \Gamma \end{array}} {{\,\mathrm{\mathbb {E}}\,}}\left( \sum _{\begin{array}{c} I\in \mathcal {D}_n,\\ I\subset J^{\Theta _{J}} \end{array}} d_I \frac{|I|}{|\Gamma |}- \sum _{\begin{array}{c} I\in \mathcal {D}_n,\\ I\subset J^{-\Theta _{J}} \end{array}} d_I \frac{|I|}{|\Gamma |} \right) ^2\\&\quad \le \frac{\max _{I\in \mathcal {D}_n, I\subset \Gamma }|d_I|^2}{|\Gamma |^2}\sum _{\begin{array}{c} J\in \mathcal {D}_m\\ J\subset \Gamma \end{array}} |J|^2 = \frac{2^{-m}}{|\Gamma |}\max _{I\in \mathcal {D}_n, I\subset \Gamma }|d_I|^2. \end{aligned}$$

$$\square $$

#### Remark 3.10

From Lemma 3.9 it follows that, under the assumption stated in the Lemma, for any $\delta >0$,

$$\begin{aligned} \mathbb {P}\left( \max _{\omega =\pm 1}\left| X_\omega - \sum _{\begin{array}{c} J\in \mathcal {D}_m\\ J\subset \Gamma \end{array}} \frac{|J|}{|\Gamma |} d_J\right| \le \delta + \left| \sum _{\begin{array}{c} J\in \mathcal {D}_m\\ J\subset \Gamma \end{array}} \frac{|J|}{|\Gamma |} d_J - \sum _{\begin{array}{c} L\in \mathcal {D}_n\\ L\subset \Gamma \end{array}} \frac{|L|}{|\Gamma |} d_L \right| \right) \ge 1 - \frac{2^{-m}}{\delta ^2|\Gamma |} \max _{\begin{array}{c} I\in \mathcal {D}_n\\ I\subset \Gamma \end{array}} d_I^2. \end{aligned}$$

Indeed, because $X_1 + X_{-1} = 2{{\,\mathrm{\mathbb {E}}\,}}(X_1) = 2{{\,\mathrm{\mathbb {E}}\,}}(X_{-1})$ we have $|X_1 - {{\,\mathrm{\mathbb {E}}\,}}(X_1)| = |X_{-1} - {{\,\mathrm{\mathbb {E}}\,}}(X_{-1})|$ and thus

$$\begin{aligned} \max _{\omega =\pm 1}\left| X_\omega - \sum _{\begin{array}{c} J\in \mathcal {D}_m\\ J\subset \Gamma \end{array}} \frac{|J|}{|\Gamma |} d_J\right|&\le \max _{\omega =\pm 1} |X_\omega - \mathbb E(X_\omega )| + \left| \sum _{\begin{array}{c} J\in \mathcal {D}_m\\ J\subset \Gamma \end{array}} \frac{|J|}{|\Gamma |} d_J - \sum _{\begin{array}{c} L\in \mathcal {D}_n\\ L\subset \Gamma \end{array}} \frac{|L|}{|\Gamma |} d_L \right| \\&= |X_1 - {{\,\mathrm{\mathbb {E}}\,}}(X_1)| + \left| \sum _{\begin{array}{c} J\in \mathcal {D}_m\\ J\subset \Gamma \end{array}} \frac{|J|}{|\Gamma |} d_J - \sum _{\begin{array}{c} L\in \mathcal {D}_n\\ L\subset \Gamma \end{array}} \frac{|L|}{|\Gamma |} d_L \right| . \end{aligned}$$

The conclusion follows from Chebyshev’s inequality.

Therefore, if we have fixed *m*, *n*, and $\Gamma$ and a varying, but finite, collection of sets of coefficients $(d_I^\alpha :I\in \mathcal {D}_n, I\subset \Gamma )$, $\alpha \in \mathcal {A}$, such that

$$\begin{aligned} \frac{2^{-m/2}}{|\Gamma |^{1/2}} \max _{\begin{array}{c} I\subset \mathcal {D}_n, I\subset \Gamma \\ \alpha \in \mathcal {A} \end{array}} |d^\alpha _I| \le \frac{\delta }{|\mathcal {A}|^{1/2}}, \end{aligned}$$

we can choose a common $(\theta _{J}: J\in \mathcal {D}_m, J\subset \Gamma )\subset \{\pm 1\}$ such that for $\omega \in \{\pm 1\}$ and

$$\begin{aligned} \Gamma ^{\omega } = \left\{ \sum _{J\in \mathcal {D}_m, J\subset \Gamma } \theta _{J} h_J = \omega \right\} \end{aligned}$$

it follows that for all $\alpha \in \mathcal {A}$,

3.4 $$\begin{aligned} \left| \sum _{\begin{array}{c} I\in \mathcal {D}_n,\\ I\subset \Gamma ^{\omega } \end{array}} d^\alpha _I \frac{|I|}{|\Gamma ^{\omega }|} - \sum _{\begin{array}{c} J\in \mathcal {D}_m,\\ J\subset \Gamma \end{array}} d^\alpha _J \frac{|J|}{|\Gamma |} \right| \le \delta + \left| \sum _{\begin{array}{c} J\in \mathcal {D}_m\\ J\subset \Gamma \end{array}} \frac{|J|}{|\Gamma |} d^\alpha _J - \sum _{\begin{array}{c} L\in \mathcal {D}_n\\ L\subset \Gamma \end{array}} \frac{|L|}{|\Gamma |} d^\alpha _L \right| . \end{aligned}$$

#### Notation 3.11

For $i\in \mathbb {N}$ and $I\in \mathcal {D}_i$ we denote $\pi (I)$ the unique $K\in \mathcal {D}_{i-1}$ such that $I = K^+$ or $I = K^-$.

#### Lemma 3.12

Let $D:\mathcal {V}(\delta )\rightarrow \mathcal {V}(\delta )$ be an $\ell ^{\infty }$-bounded operator with coefficients $(d_I)_{I\in \mathcal {D}}$, $d = \sup _{I\in \mathcal {D}} |d_{I}|$, $k_0\in \mathbb {N}_0$, and $\eta = (\eta _i)_{i=k_0}^\infty \subset (0,1)$. If $k_0 >0$, additionally assume that we have an initially defined faithful Haar system $(\tilde{h}_I)_{I\in \mathcal {D}^{k_0-1}}$ relative to frequencies $(m_i)_{i=0}^{k_0 - 1}$. Let $\mathbb {N} \supset N^{(k_0)}\supset N^{(k_0+1)}\supset \cdots$ be such that, if $m_i = \min (N^{(k_0)})$, for $i\ge k_0$, then $(m_i)_{i=0}^\infty$ is strictly increasing and let $\theta ^{(i)} = (\theta ^{(i)}_{L}: L\in \mathcal {D}_{m_i})\subset \{\pm 1\}$, $i\ge k_0$, be such that for $i\ge k_0$, $(\tilde{h}_I)_{I\in \mathcal {D}_i}$ are defined as below and the following are satisfied.

(i) If $$k_0 = 0$$ then
  $$\begin{aligned} \tilde{h}_{[0,1)} = \sum _{L\in \mathcal {D}_{m_0}} \theta ^{(0)}_{L} h_L \end{aligned}$$
  and for all $m,n\in N^{(0)}$
  $$\begin{aligned} \left| \sum _{J\in \mathcal {D}_m} |J| d_J - \sum _{L\in \mathcal {D}_n} |L| d_L\right| < \eta _0. \end{aligned}$$
(ii) For $i\ge \max \{1,k_0\}$, $I\in \mathcal {D}_i$, and $\omega \in \{\pm 1\}$ such that $I = \pi (I)^{\omega }$ we have
  $$\begin{aligned} \tilde{h}_I = \sum _{\begin{array}{c} L\in \mathcal {D}_{m_i}\\ L\subset \{\tilde{h}_{\pi (I)}=\omega \} \end{array}} \theta ^{(i)}_{L} h_L. \end{aligned}$$
(iii) For $i\ge k_0$, $I\in \mathcal {D}_i$ and $\omega \in \{\pm 1\}$
  $$\begin{aligned} \left| \sum _{\begin{array}{c} J\in \mathcal {D}_{m_i}\\ J \subset {{\,\textrm{supp}\,}}(\tilde{h}_I) \end{array}} \frac{|J|}{|I|} d_J - \sum _{\begin{array}{c} L\in \mathcal {D}_{m_{i+1}},\\ L\subset \{\tilde{h}_I=\omega \} \end{array} }\frac{|L|}{|I|/2} d_L \right| < \eta _i. \end{aligned}$$

Then for $\tilde{H} = (\tilde{h}_I)_{I\in \mathcal {D}}$ the diagonal operator $\tilde{D} = D|_{\tilde{H}}$ is $\eta$-tree-stabilized from $k_0$ on. Furthermore, if $k_0 = 0$ and $\lambda$ is a limit point of $(\sum _{J\in \mathcal {D}_n} |J| d_J)_{n\in N^{(0)}}$, then $|\tilde{d}_{[0,1)} - \lambda | \le \eta _0$.

#### Proof

It follows from (i) and (ii) that $(\tilde{h}_I:I\in \mathcal {D})$ is a faithful Haar system relative to the frequencies $(m_i)_{i=0}^\infty$. For $i\ge k_0$, $I\in \mathcal {D}_i$, and $\omega \in \{\pm 1\}$ we have, by Proposition 3.1, that

$$\begin{aligned} |\tilde{d}_I - \tilde{d}_{I^\omega }| = \left| \sum _{\begin{array}{c} J\in \mathcal {D}_{m_i}\\ J \subset {{\,\textrm{supp}\,}}(\tilde{h}_I) \end{array}} \frac{|J|}{|I|} d_J - \sum _{\begin{array}{c} L\in \mathcal {D}_{m_{i+1}},\\ L\subset \{\tilde{h}_I=\omega \} \end{array}} \frac{|L|}{|I|/2} d_L \right| < \eta _i. \end{aligned}$$

The “furthermore” statement is an immediate consequence of the second part of (i).

$$\square $$

For the purpose of the following lemma, an initially defined faithful Haar system relative to frequencies $(m_i)_{i=0}^{k_0}$ is a finite collection $(\tilde{h}_I)_{I\in \mathcal {D}^{k_0}}$ that satisfies Definition 2.16 (a) for $I\in \mathcal {D}^{k_0}$, (b) for $I\in \mathcal {D}^{k_0-1}$, and (c) for $i\le k_0$.

#### Lemma 3.13

Let $D_\alpha :\mathcal {V}(\delta )\rightarrow \mathcal {V}(\delta )$, $\alpha \in \mathcal {A}$, be a finite collection of $\ell ^{\infty }$-bounded operators, each with coefficients $(d^\alpha _I)_{I\in \mathcal {D}}$. Let $k_0\in \mathbb {N}_0$, $\delta > 0$, and *N* and be an infinite subset of $\mathbb {N}$. If $k_0 > 0$, let also $(\tilde{h}_I)_{I\in \mathcal {D}^{k_0-1}}$ be an initially defined faithful Haar system relative to frequencies $(m_i)_{i=0}^{k_0-1}$. Then, there exist an infinite subset $N^{(k_0)}$ of *N* and for $m_{k_0} = \min (N^{(k_0)})$ there exist $(\theta ^{(k_0)}_{L}: L\in \mathcal {D}_{m_{k_0}})\subset \{\pm 1\}$ such that $(\tilde{h}_I)_{I\in \mathcal {D}_{k_0}}$ are defined as follows and the following are satisfied.

(i) If $$k_0 = 0$$ then
  $$\begin{aligned} \tilde{h}_{[0,1)} = \sum _{L\in \mathcal {D}_{m_0}} \theta ^{(0)}_{L} h_L \end{aligned}$$
  and for all $m,n\in N^{(0)}$ and $\alpha \in \mathcal {A}$
  $$\begin{aligned} \left| \sum _{J\in \mathcal {D}_m} |J| d^\alpha _J - \sum _{L\in \mathcal {D}_n} |L| d^\alpha _L\right| < \delta . \end{aligned}$$
(ii) If $k_0\ge 1$ then $m_{k_0}>m_{k_0-1}$. Also, for $I\in \mathcal {D}_{k_0}$ and $\omega \in \{\pm 1\}$ such that $I = \pi (I)^{\omega }$,
  $$\begin{aligned} \tilde{h}_I = \sum _{\begin{array}{c} L\in \mathcal {D}_{m_{k_0}}\\ L\subset \{\tilde{h}_{\pi (I)}=\omega \} \end{array}} \theta ^{(k_0)}_{L} h_L. \end{aligned}$$
(iii) For $I\in \mathcal {D}_{k_0}$, $\omega \in \{\pm 1\}$, $n\in N^{(k_0)}{\setminus }\{m_{k_0}\}$, and $\alpha \in \mathcal {A}$
  $$\begin{aligned} \left| \sum _{\begin{array}{c} J\in \mathcal {D}_{m_{k_0}}\\ J \subset {{\,\textrm{supp}\,}}(\tilde{h}_I) \end{array}} \frac{|J|}{|I|} d^\alpha _J - \sum _{\begin{array}{c} L\in \mathcal {D}_{n}\\ L\subset \{\tilde{h}_I=\omega \} \end{array}} \frac{|L|}{|I|/2} d^\alpha _L \right| < \delta . \end{aligned}$$

In particular, $(\tilde{h}_I)_{I\in \mathcal {D}^{k_0}}$ is an initially defined faithful Haar system relative to the frequencies $(m_i)_{i=0}^{k_0}$.

#### Proof

The only notable difference in the case $k_0 = 0$ is that the second part of (i) must be additionally achieved. This can be easily achieved using the $\ell ^{\infty }$-boundedness of the given diagonal operators. Therefore, we assume that $k_0 \ge 1$. For each $I\in \mathcal {D}_{k_0}$ denote $\tilde{I} = \{\tilde{h}_{\pi (I)} = \xi \}$, where $\xi \in \{\pm 1\}$ is such that $I = \pi (I)^\xi$. Choose an infinite $M\subset N$ with $m_{k_0} = \min (M) >m_{k_0-1}$ and

$$\begin{aligned} 2^{(k_0-m_{k_0})/2}\max _{\alpha \in \mathcal {A}}\Vert D_\alpha \Vert _\infty < \frac{\delta /2}{|\mathcal {A}|^{1/2}} \end{aligned}$$

such that for all $I\in \mathcal {D}_{k_0}$ and $m,n\in M$ we have

3.5 $$\begin{aligned} \left| \sum _{\begin{array}{c} K\in \mathcal {D}_{m}\\ K\subset \tilde{I} \end{array}} \frac{|K|}{|I|} d^\alpha _K - \sum _{\begin{array}{c} L\in \mathcal {D}_{n}\\ L\subset \tilde{I} \end{array}} \frac{|L|}{|I|} d^\alpha _L \right| < \delta /2. \end{aligned}$$

We next wish to apply Remark 3.10 and specify parameters *m*, *n* and $\Gamma$. We take $m = m_{k_0}$ and, for $I\in \mathcal {D}_{k_0}$, take $\Gamma = \tilde{I}$. We consider these parameters to be fixed and proceed with the argument for arbitrary $n\in M\setminus \{m_{k_0}\}$, which will lead to an infinite choice of appropriate *n* to define our set $N^{(k_0)}$.

By (3.5) and (3.4) we can find $\kappa _{I,n} = (\theta _{n,L}: L\in \mathcal {D}_m,L\subset \tilde{I})$ such that for $\omega \in \{\pm 1\}$ and

$$\begin{aligned} \tilde{I}_n^{\omega } = \left\{ \sum _{J\in \mathcal {D}_m, J\subset \tilde{I}} \theta _{n,J}h_J = \omega \right\} \end{aligned}$$

it follows that for all $\alpha \in \mathcal {A}$,

3.6 $$\begin{aligned} \left| \sum _{\begin{array}{c} L\in \mathcal {D}_n,\\ L\subset \tilde{I}_n^{\omega } \end{array}} \frac{|L|}{|I|/2} d^\alpha _L - \sum _{\begin{array}{c} J\in \mathcal {D}_{m_{k_0}},\\ J\subset \tilde{I} \end{array}} \frac{|J|}{|I|} d^\alpha _J \right| < \delta . \end{aligned}$$

By the pigeonhole principle, we may choose an infinite subset $\Lambda$ of *M* such that for all $I\in \mathcal {D}_{k_0}$ and $n,n'\in \Lambda$ we have $\kappa _{I,n} = \kappa _{I,n'}$. Define $N^{(k_0)} = \{m_{k_0}\}\cup \Lambda$ and concatenate the selected vectors of signs $\kappa _{I,n}$, $I\in \mathcal {D}_{k_0}$, and $n\in \Lambda$ by (slightly abusing the notation and) putting $\theta ^{(k_0)} = (\kappa _{I,n}: I\in \mathcal {D}_{k_0})$. For each $I\in \mathcal {D}_{k_0}$ define

$$\begin{aligned} \tilde{h}_I = \sum _{\begin{array}{c} L\in \mathcal {D}_{m_{k_0}}\\ L\subset \tilde{I} \end{array}} \theta ^{(k_0)}_{L} h_L \end{aligned}$$

and thus, for $\omega \in \{\pm 1\}$, $\{\tilde{h}_I = \omega \} = \tilde{I}_\omega$. In other words, (3.6) yields (iii).$\square$

#### Proof of Proposition 3.8

In this game, Player (II) recursively applies Lemma 3.13, in each step *k*, to the finite collection $\cup _{i=0}^k\mathcal {F}_i$ and using the set $M_k$ and $\eta _k$ given by Player (I) to achieve the assumptions of Lemma 3.12 for each $\mathcal {F}_{k_0}$ from $k_0$ on. $\square$

### The 2-parameter case

#### Proposition 3.14

Let $\tilde{H}\otimes \tilde{K}= (\tilde{h}_I\otimes \tilde{k}_J: I,J\in \mathcal {D})$ be a faithful Haar system relative to the frequencies $(m_i)$ and $(n_j)$, and let $D:\mathcal {V}(\delta ^2)\rightarrow \mathcal {V}(\delta ^2)$ be a diagonal operator. Then $\tilde{D} = D|_{\tilde{H}\otimes \tilde{K}}$ is a diagonal operator with coefficients $(\tilde{d}_{I,J}: I,J\in \mathcal {D})$ given by

$$\begin{aligned} \tilde{d}_{ I,J} = \sum _{\begin{array}{c} L\in \mathcal {D}_{m_i},\\ L\subset {{\,\textrm{supp}\,}}(\tilde{h}_I) \end{array}} \frac{|L|}{|I|} \sum _{\begin{array}{c} M\in \mathcal {D}_{n_j},\\ M\subset {{\,\textrm{supp}\,}}( \tilde{k}_J) \end{array}} \frac{|M|}{|J|} d_{L,M} \end{aligned}$$

for $$i,j\in \mathbb {N}_0, I\in \mathcal {D}_i$$ and $$J\in \mathcal {D}_j$$.

#### Proof

For $I\in \mathcal {D}_i$ write $\tilde{h}_I$ and $\tilde{k}_I$ as

$$\begin{aligned} \tilde{h}_I = \sum _{L\in \mathcal {A}_I} \theta _L h_L \qquad \text {and}\qquad \tilde{k}_I = \sum _{M\in \mathcal {B}_I } \varepsilon _M k_M, \end{aligned}$$

with $\mathcal {A}_I\subset \mathcal {D}_{m_i}$, $\mathcal {B}_I\subset \mathcal {D}_{n_i}$, $(\theta _L: L\in \mathcal {A}_I), (\varepsilon _M: M\in \mathcal {B}_I)\subset \{\pm 1\}$. Let $i,i',j,j'\in \mathbb {N}_0$, and $I\in \mathcal {D}_i$, $I'\in \mathcal {D}_{i'}$, $J\in \mathcal {D}_j$ and $J'\in \mathcal {D}_{j'}$. Then

$$\begin{aligned} \langle \tilde{h}_{I'}\otimes \tilde{k}_{J'}, D(\tilde{h}_{I}\otimes \tilde{k}_{J}) \rangle&= \left\langle \sum _{L'\in \mathcal {A}_{I'}} \theta _{L'} h_{L'}\otimes \tilde{k}_{J'}, D\left( \sum _{L\in \mathcal {A}_I} \theta _L h_{L}\otimes \tilde{k}_J\right) \right\rangle \\&= \sum _{L'\in \mathcal {A}_{I'}, L\in \mathcal {A}_I} \theta _{L'}\theta _L \left\langle h_{L'}\otimes \tilde{k}_{J'}, h_L\otimes D_{L,\cdot }( \tilde{k}_J)\right\rangle . \end{aligned}$$

where $D_{L,\cdot }:\mathcal {V}(\delta )\rightarrow \mathcal {V}(\delta )$ is the 1-parameter diagonal operator defined by

$$\begin{aligned} D_{L,\cdot }(h_M) = d_{L,M} h_M, \qquad L,M\in \mathcal {D}. \end{aligned}$$

Thus by Proposition 3.1, we obtain for $J\ne J'$ that

$$\begin{aligned} \langle \tilde{h}_{I'}\otimes \tilde{k}_{J'}, D(\tilde{h}_{I}\otimes \tilde{k}_{J})\rangle = \sum _{\begin{array}{c} L'\in \mathcal {A}(I')\\ L\in \mathcal {A}(I) \end{array}} \theta _{I'}\theta _I \langle h_L,h_{L'}\rangle \langle \tilde{k}_{J'}, D_{L,\cdot }( \tilde{k}_J)\rangle = 0. \end{aligned}$$

Similarly using Proposition 3.1 in the case that $J=J'$, and $I\ne I'$, we obtain that

$$\begin{aligned} \langle \tilde{h}_{I'}\otimes k_{L'}, D(\tilde{h}_{K}\otimes k_{L}) \rangle&= \sum _{\begin{array}{c} L'\in \mathcal {A}_{I'}\\ L\in \mathcal {A}_I \end{array}} \theta _{L'}\theta _L \langle h_L,h_{L'}\rangle \langle \tilde{k}_{J}, D_{L,\cdot }( \tilde{k}_J)\rangle \\&= \sum _{\begin{array}{c} L'\in \mathcal {A}_{I'}\\ L\in \mathcal {A}_I \end{array}} \theta _{L'}\theta _{L} \langle h_{L'},h_{L}\rangle \sum _{M\in \mathcal {B}_J} |M| d_{L,M} = 0. \end{aligned}$$

In the case that $I=I'$ and $J=J'$, we obtain that

$$\begin{aligned} \langle \tilde{h}_{I'}\otimes k_{J'}, D(\tilde{h}_{I}\otimes k_{J}) \rangle&= \sum _{L\in \mathcal {A}_I} |L| \sum _{M\in \mathcal {B}_J} |M| d_{L,M}. \end{aligned}$$

It follows, therefore, that

$$\begin{aligned} A\circ D\circ B(h_I \otimes k_J)&= \sum _{I',J'\in \mathcal {D}}\frac{h_{I'} \otimes k_{J'} }{|I'|\cdot |J'|} \langle \tilde{h}_{I'}\otimes \tilde{k}_{J'} , D(\tilde{h}_{I}\otimes \tilde{k}_{J})\rangle \\&= \frac{h_{I} \otimes k_{J} }{|I|\cdot |J|} \sum _{L\in \mathcal {A}_I} |L| \sum _{M\in \mathcal {B}_J} |M| d_{L,M}\\&= h_I \otimes k_J\sum _{\begin{array}{c} L\in \mathcal {D}_{m_i}\\ L\subset {{\,\textrm{supp}\,}}(\tilde{h}_I) \end{array}} \frac{|L|}{|I|} \sum _{\begin{array}{c} M\in \mathcal {D}_{n_j}\\ M\subset {{\,\textrm{supp}\,}}(\tilde{k}_J) \end{array}} \frac{|M|}{|J|} d_{L,M}. \end{aligned}$$

$$\square $$

As in the one-parameter case, in the two-parameter case, starting with an $\ell ^{\infty }$ diagonal operator and iterating the process of passing to faithful Haar systems is in a sense stable.

#### Notation 3.15

Let $\widetilde{H}^{(1)}$, $\widetilde{K}^{(1)}$ be a faithful Haar system relative to frequencies $(m_i)_{i=0}^\infty$ and $(n_i)_{i=0}^\infty$ respectively and let $\widetilde{H}^{(2)}$, $\widetilde{K}^{(2)}$ be a faithful Haar system relative to frequencies $(s_i)_{i=0}^\infty$ and $(t_i)_{i=0}^\infty$ respectively. We define

$$\begin{aligned} (\widetilde{H}^{(2)}\otimes \widetilde{K}^{(2)}) *(\widetilde{H}^{(1)} \otimes \widetilde{K}^{(1)}) = (\widetilde{H}^{(2)}*\widetilde{H}^{(1)}) \otimes (\widetilde{K}^{(2)}*\widetilde{K}^{(1)}). \end{aligned}$$

The proof of the following is an easy adaptation of the proof Corollary 3.4 using Notation 2.28 and Notation 3.15.

#### Proposition 3.16

Let $\widetilde{H}^{(1)}$, $\widetilde{K}^{(1)}$ be a faithful Haar system relative to frequencies $(m_i)_{i=0}^\infty$ and $(n_i)_{i=0}^\infty$ respectively and let $\widetilde{H}^{(2)}$, $\widetilde{K}^{(2)}$ be a faithful Haar system relative to frequencies $(s_i)_{i=0}^\infty$ and $(t_i)_{i=0}^\infty$ respectively. Then, for every $\ell ^{\infty }$-bounded diagonal operator $D:\mathcal {V}(\delta ^2)\rightarrow \mathcal {V}(\delta ^2)$,

$$\begin{aligned} (D|_{\widetilde{H}^{(1)}\otimes \widetilde{K}^{(1)}})|_{\widetilde{H}^{(2)} \otimes \widetilde{K}^{(2)}} = D|_{(\widetilde{H}^{(2)}\otimes \widetilde{K}^{(2)})*(\widetilde{H}^{(1)} \otimes \widetilde{K}^{(1)})}. \end{aligned}$$

### Bi-tree-semi-stabilized operators in two parameters

This is a notion on diagonal operators on $\mathcal {V}(\delta ^2)$ that means that they can be “split” into two pieces, an upper triangular and a lower triangular one, each of which has essentially constant coefficients.

#### Definition 3.17

(*Bi-tree-semi-stabilized operators in two parameters*) Let $D:\mathcal {V}(\delta ^2)\rightarrow \mathcal {V}(\delta ^2)$ be a diagonal operator whose coefficients we denote by $(d_{I,J}: I,J\in \mathcal {D})$ and let $\eta = (\eta _{i,j})_{i,j=0}^\infty \in (0,1)^{\mathbb {N}^2}$ and $\delta >0$. We say that *D* satisfies:

- *The lower triangular condition for* $\eta$ if for all $i\ge j\ge 0$, $I\in \mathcal {D}_i$, $J\in \mathcal {D}_j$, and $\omega \in \{\pm 1\}$
  $$\begin{aligned} |d_{I^\omega ,J} - d_{I,J}| < \eta _{i,j}. \end{aligned}$$
- *The upper triangular condition for* $\eta$ if for all $j > i \ge 0$, $I\in \mathcal {D}_i$, $J\in \mathcal {D}_j$, and $\xi \in \{\pm 1\}$
  $$\begin{aligned} |d_{I,J} - d_{I,J^\xi }| < \eta _{i,j}. \end{aligned}$$
- *The diagonal condition for* $\eta$ if for all $i\ge 0$, $I,J\in \mathcal {D}_i$, and $\omega ,\xi \in \{\pm 1\}$ then
  $$\begin{aligned} |d_{I,J} - d_{I^\omega ,J^\xi }| \le \eta _{i,i}. \end{aligned}$$
- *The superdiagonal condition for* $\eta$ if for all $i\ge 0$, $I\in \mathcal {D}_{i}$ and $J\in \mathcal {D}_{i+1}$, and $\omega ,\xi \in \{\pm 1\}$ then
  $$\begin{aligned} |d_{I,J} -d_{I^\omega ,J^\xi }| \le \eta _{i,i+1}. \end{aligned}$$
- *The balancing condition for* $\delta$ if we have $|d_{[0,1),[0,1/2)} - d_{[0,1),[1/2,1)}| < \delta$.

If (a)–(e) are all satisfied then we call *D* $(\eta ,\delta )$-*bi-tree-semi-stabilized*. (For a visualization we refer to the figures below.)


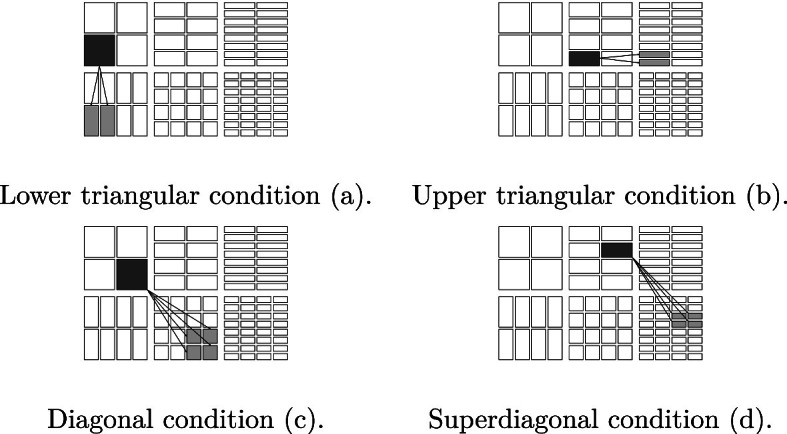


#### Remark 3.18

Conditions (a) and (b) of Definition 3.17 may be reformulated as follows.

(a’) (The lower triangular condition) For $j\in \mathbb {N}_0$ and $J\in \mathcal {D}_j$, the diagonal operator $D_{(\cdot ,J)}:\mathcal {V}(\delta )\rightarrow \mathcal {V}(\delta )$, whose coefficients are $(d_{I,J}: I\in \mathcal {D})$ is $(\eta _{i,j})_{i=0}^\infty$-stabilized from *j* on.
(b’) (The upper triangular condition) For $i\in \mathbb {N}_0$ and $I\in \mathcal {D}_i$, the diagonal operator $D_{(I,\cdot )}:\mathcal {V}(\delta )\rightarrow \mathcal {V}(\delta )$ whose coefficients are $(d_{I,J}: I\in \mathcal {D})$ is $(\eta _{i,j})_{j=0}^\infty$-stabilized from $i+1$ on.

The above will be used later to apply one-parametric stabilization arguments to a bi-parameter Haar multiplier.

The following is the bi-parameter version of Remark 3.6. Observe that, in this case, a bi-tree-semi-stabilized Haar multiplier is not close to a scalar operator.

#### Remark 3.19

Let $D:\mathcal {V}(\delta ^2)\rightarrow \mathcal {V}(\delta ^2)$ be a Haar multiplier, $\eta = (\eta _{i,j})_{i,j=0}^\infty \in (0,1)^{\mathbb {N}^2}$, and $\delta >0$. Assume that *D* is $(\eta ,\delta )$-bi-tree-semi-stabilized and $\sum _{i,j=0}^\infty 2^{i+j}\eta _{i,j}<\infty$. Then,

(i) ${\lambda (D) = \lim _{j\rightarrow \infty } \lim _{i\rightarrow \infty } \sum _{I\in \mathcal {D}_i}\sum _{J\in \mathcal {D}_j}|I||J|d_{I,J}}$ and $\mu (D) = \lim _{i\rightarrow \infty } \lim _{j\rightarrow \infty } \sum _{I\in \mathcal {D}_i}\sum _{J\in \mathcal {D}_j}|I||J|d_{I,J}$ exist and
(ii) $${\left\| D - \left( \lambda (D)\mathcal {C} + \mu (D)({{\,\textrm{Id}\,}}-\mathcal {C})\right) \right\| _{\mathrm {T^2}}\le \sum _{i=0}^\infty \sum _{j=0}^\infty (i+j+4)\eta _{i,j} + \delta }$$.

In particular, for any non-principal ultrafilter $\mathcal {U}$ on $\mathbb {N}$, $\lambda _\mathcal {U}(D) = \lambda (D)$ and $\mu _\mathcal {U}(D) = \mu (D)$.

This estimate yields that a bi-tree-semi-stabilized diagonal operator is in $\Vert \cdot \Vert _{\mathrm {T^2}}$-norm close to a diagonal operator $\lambda \mathcal {C} + \mu ({{\,\textrm{Id}\,}}- \mathcal {C})$. If *Z* is a Capon space, this proximity can also be achieved in operator norm (see Theorem 2.32). The proof of the above goes along the same lines as the proof of Proposition 3.22 further below.

The main theorem of this section states that for every $\ell ^\infty$-bounded Haar multiplier *D* there exist faithful Haar systems such that the corresponding $\tilde{D}$ is bi-tree-semi-stabilized. Furthermore, the values $\lambda$ and $\mu$ can, up to an arbitrarily small error, be determined by the valuation of the linear functionals $\lambda _\mathcal {U}(\cdot ),\mu _\mathcal {U}(\cdot )$ defined on the vector space of all $\ell ^\infty$-bounded bi-parameter Haar multipliers.

#### Theorem 3.20

Let $D:\mathcal {V}(\delta ^2)\rightarrow \mathcal {V}(\delta ^2)$ be a diagonal operator whose coefficients we denote by $(d_{I,J}: I,J\in \mathcal {D})$ which is $\ell ^{\infty }$-bounded, $\eta = (\eta _{i,j} )_{i,j=0}^\infty \in (0,1)^{\mathbb {N}^2}$, and $\delta >0$. Then for any infinite *M*, $N\subset \mathbb {N}$ there exist faithful Haar systems $\tilde{H}=(\tilde{h}_I:I\in \mathcal {D})$ and $\tilde{K}=( \tilde{k}_J:J\in \mathcal {D})$ relative to frequencies $(m_i)_{i=0}^\infty$ in *M* and $(n_j)_{j=0}^\infty$ in *N*, such that

$$\begin{aligned} \tilde{D} = D|_{\tilde{H}\otimes \tilde{K}} \quad \text {is}\quad (\eta ,\delta )-\quad \text {bi-tree-semi-stabilized}. \end{aligned}$$

In particular, for any $\mathcal {U}\in \beta \mathbb {N}{\setminus } \mathbb {N}$, we may choose $\tilde{D}$ such that $\lambda _\mathcal {U}(\tilde{D}) = \lambda _\mathcal {U}(D)$ and $\mu _\mathcal {U}(\tilde{D}) = \mu _\mathcal {U}(D)$ and, thus,

$$\begin{aligned} \left\| \tilde{D} - \left( \lambda _\mathcal {U}(D)\mathcal {C}+\mu _\mathcal {U}({{\,\textrm{Id}\,}}-\mathcal {C})\right) \right\| {_{\mathrm {T^2}}} \le \sum _{i=0}^\infty \sum _{j=0}^\infty (i+j+4)\eta _{i,j} + \delta . \end{aligned}$$

Moreover, $$|\tilde{d}_{[0,1),[0,1)} - \lambda (D)| < \delta $$ and $$|\tilde{d}_{[0,1),[0,1/2)} - \mu (D)| < \delta $$.

### Permanence of stability under blocking

To make the proof of Theorem 3.20 more tractable, we will show, separately, how each of properties (a)–(e) of Definition 3.17 can be achieved in isolation by appropriately blocking the Haar system in each parameter. The permanence of (a)–(e), under appropriate blocking, guarantees that the resulting diagonal operator satisfies all of them.

We start with a summability assumption on sequences $(\eta _{i,j})_{i,j=1}^\infty$ with respect to which we consider semi-stability the purpose of which is to improve notation.

#### Assumption 3.21

Assume $\eta = (\eta _{i,j} )_{i,j=0}^\infty \in (0,1)^{\mathbb {N}^2}$ has the property

3.7 $$\begin{aligned} \sum _{j=j_0+1}^\infty \eta _{i_0,j}< \frac{\eta _{i_0,j_0}}{3} \quad \text {and}\quad \sum _{i=i_0+1}^\infty \eta _{i,j_0} < \frac{\eta _{i_0,j_0}}{3}, \qquad \text {for }i_0,j_0\in \mathbb {N}. \end{aligned}$$

#### Proposition 3.22

Let $D:\mathcal {V}(\delta ^2)\rightarrow \mathcal {V}(\delta ^2)$ be an $\ell ^\infty$-bounded Haar multiplier whose coefficients are $(\eta _{i,j})_{i,j=0}^\infty$-bi-tree-semi-stabilized and assume (3.7). Let $(K_0,L_0)$, $(K_1,L_1)\in \mathcal {D}\times \mathcal {D}$ such that $K_0\supset K_1$ and $L_0\supset L_1$.

(i) If $|K_0|\le |L_0|$ and $|K_1|\le |L_1|$, i.e., both $(K_0,L_0)$ and $(K_1,L_1)$ are in the lower triangle part of $\mathcal {D}\times \mathcal {D}$, then
  $$\begin{aligned} |d_{(K_0,L_0)} - d_{(K_1,L_1)}| \le \eta _{l_0-1,l_0-1}, \end{aligned}$$
  where $$2^{-l_0} = |L_0|.$$
(ii) If $|K_0| > |L_0|$ and $|K_1| > |L_1|$, i.e., both $(K_0,L_0)$ and $(K_1,L_1)$ are in the upper triangle part of $\mathcal {D}\times \mathcal {D}$, then
  $$\begin{aligned} |d_{(K_0,L_0)} - d_{(K_1,L_1)}| \le \eta _{k_0-1,k_0}, \end{aligned}$$
  where $$2^{-k_0} = |K_0|.$$

#### Proof

We only prove (i). The proof of (ii) is similar. The most efficient way to obtain these estimates is by telescoping along a specific path contained in the lower triangular submatrix. Put $2^{-k_1} = |K_1|$, $2^{-l_1} = |L_1|$. The path is constructed by first traversing upwards from both points $(K_i,L_i)$, $i=0,1$ until they hit the diagonal and then join those two points on the diagonal by moving along the diagonal. The vertical paths $P_i$, $i=0,1$ are given by

$$\begin{aligned} P_i = \left\{ (K,L_i) : K_i' \supsetneq K \supset K_i \right\} , \end{aligned}$$

where $K_i'$ defined as the unique dyadic interval containing $K_i$ such that $|K_i'| = |L_i| = 2^{-l_i}$ for $i=0,1$. Now we join the above vertical paths along the diagonal:

$$\begin{aligned} P_2 = \left\{ (K,L) : K_0'\supsetneq K \supset K_1',\ L_0\supsetneq L \supset L_1,\ |K| = |L| \right\} . \end{aligned}$$

We will now estimate $|d_{K_0,L_0} - d_{K_1,L_1}|$ by telescoping first along $P_0$, then $P_{2}$ and finally $P_1$:

3.8 $$\begin{aligned} |d_{K_0,L_0} - d_{K_1,L_1}| \le \sum _{i=0}^1 \sum _{(K,L_i)\in P_i} |d_{(K,L_i)} - d_{(\pi (K),L_i)}| + \sum _{(K,L)\in P_2} |d_{(K,L)} - d_{(\pi (K),\pi (L))}|. \end{aligned}$$

By the lower triangular and diagonal condition (see Definition 3.17 (a) and (c)), we obtain

3.9 $$\begin{aligned} \begin{aligned} |d_{(K,L_i)} - d_{(\pi (K),L_i)}|&\le \eta _{k-1,l_i},{} & {} (K,L_i)\in P_i,\ |K|=2^{-k},\ i=0,1,\\ |d_{(K,L)} - d_{(\pi (K),\pi (L))}|&\le \eta _{k-1,k-1},{} & {} |K| = |L| = 2^{-k} \end{aligned} \end{aligned}$$

Inserting (3.9) into (3.8) and using (3.7) yields

3.10 $$\begin{aligned} \begin{aligned} |d_{K_0,L_0} - d_{K_1,L_1}|&\le \sum _{i=0}^1 \sum _{k=l_i+1}^{k_i} \eta _{k-1,l_i} + \sum _{k=l_0+1}^{l_1} \eta _{k-1,k-1} \le \sum _{i=0}^1 \frac{1}{3}\eta _{l_i-1,l_i} + \frac{1}{9}\eta _{l_0-1,l_0-1}\\&\le \eta _{l_0-1,l_0-1}. \end{aligned} \end{aligned}$$

#### Proposition 3.23

Assume that $D:\mathcal {V}(\delta ^2)\rightarrow \mathcal {V}(\delta ^2)$ is a diagonal operator which is $\ell ^{\infty }$-bounded and $\eta =(\eta _{i,j} )_{i,j=0}^\infty \in (0,1)^{\mathbb {N}^2}$ that satisfies (3.7). Let $\tilde{H}=(\tilde{h}_K:K\in \mathcal {D})$ and $\tilde{K}=( \tilde{k}_L:L\in \mathcal {D})$ be faithful Haar systems relative to the frequencies $(m_i)_{i=0}^\infty$ and $(n_j)_{j=0}^\infty$ respectively, such that, for $1\le n_i< m_i < n_{i+1}$, $i\ge 0$. Denote $\tilde{D}= D|_{\tilde{H}\otimes \tilde{K}}$.

(i) If *D* satisfies the lower triangular condition for $\eta$ then $\tilde{D}$ satisfies the lower triangular condition for $\eta$.
(ii) If *D* satisfies the upper triangular condition for $\eta$ then $\tilde{D}$ satisfies the upper triangular condition for $\eta$.
(iii) If *D* satisfies the lower triangular condition and the diagonal condition for $\eta$ then $\tilde{D}$ satisfies the diagonal condition for $\eta$.
(iv) If *D* satisfies the upper triangular condition and the superdiagonal condition for $\eta$ then $\tilde{D}$ satisfies the superdiagonal condition for $\eta$.
(v) If *D* satisfies the upper triangular condition and the superdiagonal condition for $\eta$ and the balancing condition for some $\delta >0$ then $\tilde{D}$ satisfies the balancing condition for $\tilde{\delta }= \delta +\sum _{i,j}\eta _{i,j}$.

#### Proof of Proposition 3.8

Since the proof of (i) and (ii) as well as the proof of (iii) and (iv) are completely analogous, we will only present the proof for (i) and (iii). In all cases, the proof comes down to expressing a difference of successive entries of $\tilde{D}$ as an average of distances along paths and then evoking Proposition 3.22. We do not prove Item (v), because we do not use it, but it follows with similar arguments.

Let $(d_{K,L}:K,L\in \mathcal {D})$ be the coefficients of *D* and $(\tilde{d}_{K,L}:K,L\in \mathcal {D})$ be the coefficients of $\tilde{D}=D|_{\tilde{H}\otimes \tilde{K}}$.

#### Proof of (i)

For $k\ge l$, $I\in \mathcal {D}_k$ and $J\in \mathcal {D}_l$, and $\theta \in \{\pm 1\}$ we have

3.11 $$\begin{aligned} \begin{aligned} |\tilde{d}_{I,J}-\tilde{d}_{I^\theta ,J}|&= \left| \sum _{\begin{array}{c} K\in \mathcal {D}_{m_k}\\ K\subset {{\,\textrm{supp}\,}}(\tilde{h}_I) \end{array}} \sum _{\begin{array}{c} L\in \mathcal {D}_{n_{l}}\\ L\subset {{\,\textrm{supp}\,}}( \tilde{k}_J) \end{array}} \frac{|K| |L|}{|I| |J|} d_{K,L} - \sum _{\begin{array}{c} K\in \mathcal {D}_{m_{k+1}}\\ K\subset {{\,\textrm{supp}\,}}(\tilde{h}_{I^{\theta }}) \end{array}} \sum _{\begin{array}{c} L\in \mathcal {D}_{n_{l}}\\ L\subset {{\,\textrm{supp}\,}}( \tilde{k}_J) \end{array}} \frac{|K| |L|}{|I^{\theta }| |J|} d_{K,L} \right| \\&\le \sum _{\begin{array}{c} L\in \mathcal {D}_{n_{l}}\\ L\subset {{\,\textrm{supp}\,}}( \tilde{k}_J) \end{array}} \frac{|L|}{|J|} \left| \sum _{\begin{array}{c} K\in \mathcal {D}_{m_k}\\ K\subset {{\,\textrm{supp}\,}}(\tilde{h}_I) \end{array}} \frac{|K|}{|I|} d_{K,L} - \sum _{\begin{array}{c} K\in \mathcal {D}_{m_{k+1}}\\ K\subset {{\,\textrm{supp}\,}}(\tilde{h}_{I^{\theta }}) \end{array}} \frac{|K|}{|I^{\theta }|} d_{K,L} \right| . \end{aligned} \end{aligned}$$

Next, we record the identity

3.12 $$\begin{aligned}{} & {} \sum _{\begin{array}{c} K\in \mathcal {D}_{m_k}\\ K\subset {{\,\textrm{supp}\,}}(\tilde{h}_I) \end{array}} \frac{|K|}{|I|} d_{K,L} - \sum _{\begin{array}{c} K\in \mathcal {D}_{m_{k+1}}\\ K\subset {{\,\textrm{supp}\,}}(\tilde{h}_{I^{\theta }}) \end{array}} \frac{|K|}{|I^{\theta }|} d_{K,L}\nonumber \\{} & {} = \sum _{\begin{array}{c} K_0\in \mathcal {D}_{m_k}\\ K_0\subset {{\,\textrm{supp}\,}}(\tilde{h}_I) \end{array}} \frac{|K_0|}{|I|} \left( d_{K_0,L} - 2\cdot \sum _{\begin{array}{c} K\in \mathcal {D}_{m_{k+1}}\\ K\subset K_0^{\theta } \end{array}} \frac{|K|}{|K_{0}|} d_{K,L}\right) . \end{aligned}$$

Combining (3.11) and (3.12) yields

3.13 $$\begin{aligned} \begin{aligned} |\tilde{d}_{I,J}-\tilde{d}_{I^\theta ,J}|&\le \sum _{\begin{array}{c} L\in \mathcal {D}_{n_{l}}\\ L\subset {{\,\textrm{supp}\,}}( \tilde{k}_J) \end{array}} \frac{|L|}{|J|} \sum _{\begin{array}{c} K_0\in \mathcal {D}_{m_k}\\ K_0\subset {{\,\textrm{supp}\,}}(\tilde{h}_I) \end{array}} \frac{|K_0|}{|I|}\cdot \left| d_{K_0,L} - 2\cdot \sum _{\begin{array}{c} K\in \mathcal {D}_{m_{k+1}}\\ K\subset K_0^{\theta } \end{array}} \frac{|K|}{|K_{0}|} d_{K,L} \right| \\&\le \sum _{\begin{array}{c} L\in \mathcal {D}_{n_{l}}\\ L\subset {{\,\textrm{supp}\,}}( \tilde{k}_J) \end{array}} \frac{|L|}{|J|} \sum _{\begin{array}{c} K_0\in \mathcal {D}_{m_k}\\ K_0\subset {{\,\textrm{supp}\,}}(\tilde{h}_I) \end{array}} \frac{|K_0|}{|I|} \sum _{\begin{array}{c} K\in \mathcal {D}_{m_{k+1}}\\ K\subset K_0^{\theta } \end{array}} \frac{|K|}{|K_{0}|} |d_{K_0,L} - d_{K,L}|, \end{aligned} \end{aligned}$$

where, by Proposition 3.22 (i), each $|d_{K_0,L} - d_{K,L}| \le \eta _{n_l-1,n_l-1} \le \eta _{l,l} \le \eta _{k,l}$. Hence, since

$$\begin{aligned} \sum _{\begin{array}{c} L\in \mathcal {D}_{n_{l}}\\ L\subset {{\,\textrm{supp}\,}}( \tilde{k}_J) \end{array}} \frac{|L|}{|J|} \sum _{\begin{array}{c} K_0\in \mathcal {D}_{m_k}\\ K_0\subset {{\,\textrm{supp}\,}}(\tilde{h}_I) \end{array}} \frac{|K_0|}{|I|} \sum _{\begin{array}{c} K\in \mathcal {D}_{m_{k+1}}\\ K\subset K_0^{\theta } \end{array}} \frac{|K|}{|K_{0}|} = 1, \end{aligned}$$

our estimates (3.13) yields $|\tilde{d}_{I,J}-\tilde{d}_{I^\theta ,J}| \le \eta _{k,l}$, as claimed.

#### Proof of (iii)

For $k\ge 0$, $I,J\in \mathcal {D}_k$ and $\theta ,\varepsilon \in \{\pm 1\}$ we have

3.14 $$\begin{aligned} \begin{aligned}&|\tilde{d}_{I,J}-\tilde{d}_{I^\theta ,J^\varepsilon }| \\&\quad = \left| \sum _{\begin{array}{c} K_0\in \mathcal {D}_{m_k}\\ K_0\subset {{\,\textrm{supp}\,}}(\tilde{h}_I) \end{array}} \sum _{\begin{array}{c} L_0\in \mathcal {D}_{n_{k}}\\ L_0\subset {{\,\textrm{supp}\,}}( \tilde{k}_J) \end{array}} \frac{|K_0| |L_0|}{|I| |J|} d_{K_0,L_0} - 4\cdot \sum _{\begin{array}{c} K\in \mathcal {D}_{m_{k+1}}\\ K\subset {{\,\textrm{supp}\,}}(\tilde{h}_{I^{\theta }}) \end{array}} \sum _{\begin{array}{c} L\in \mathcal {D}_{n_{k+1}}\\ L\subset {{\,\textrm{supp}\,}}( \tilde{k}_{J^\varepsilon }) \end{array}} \frac{|K| |L|}{|I| |J|} d_{K,L} \right| \\&\quad \le \left| \sum _{\begin{array}{c} K_0\in \mathcal {D}_{m_k}\\ K_0\subset {{\,\textrm{supp}\,}}(\tilde{h}_I) \end{array}} \frac{|K_0|}{|I|} \sum _{\begin{array}{c} L_0\in \mathcal {D}_{n_{k}}\\ L_0\subset {{\,\textrm{supp}\,}}( \tilde{k}_J) \end{array}} \frac{|L_0|}{|J|} \left( d_{K_0,L_0} - 4\cdot \sum _{\begin{array}{c} K\in \mathcal {D}_{m_{k+1}}\\ K\subset K_0^{\theta } \end{array}} \frac{|K|}{|K_0|} \sum _{\begin{array}{c} L\in \mathcal {D}_{n_{k+1}}\\ L\subset L_0^{\varepsilon } \end{array}} \frac{|L|}{|L_0|} d_{K,L} \right) \right| . \end{aligned} \end{aligned}$$

Moreover, since

$$\begin{aligned} 4\cdot \sum _{\begin{array}{c} K\in \mathcal {D}_{m_{k+1}}\\ K\subset K_0^{\theta } \end{array}} \frac{|K|}{|K_0|} \sum _{\begin{array}{c} L\in \mathcal {D}_{n_{k+1}}\\ L\subset L_0^{\varepsilon } \end{array}} \frac{|L|}{|L_0|} = 1, \end{aligned}$$

we further estimate (3.14) by

$$\begin{aligned}&|\tilde{d}_{I,J}-\tilde{d}_{I^\theta ,J^\varepsilon }| \\&\quad \le 4\cdot \left| \sum _{\begin{array}{c} K_0\in \mathcal {D}_{m_k}\\ K_0\subset {{\,\textrm{supp}\,}}(\tilde{h}_I) \end{array}} \frac{|K_0|}{|I|} \sum _{\begin{array}{c} L_0\in \mathcal {D}_{n_{k}}\\ L_0\subset {{\,\textrm{supp}\,}}( \tilde{k}_J) \end{array}} \frac{|L_0|}{|J|} \sum _{\begin{array}{c} K\in \mathcal {D}_{m_{k+1}}\\ K\subset K_0^{\theta } \end{array}} \frac{|K|}{|K_0|} \sum _{\begin{array}{c} L\in \mathcal {D}_{n_{k+1}}\\ L\subset L_0^{\varepsilon } \end{array}} \frac{|L|}{|L_0|} \bigl (d_{K_0,L_0} - d_{K,L}\bigr ) \right| . \end{aligned}$$

Recall the by Proposition 3.22 (i) $|d_{K_0,L_0} - d_{K,L}|\le \eta _{n_{k}-1,n_{k}-1} \le \eta _{k,k}$, and thus by convexity $|\tilde{d}_{I,J}-\tilde{d}_{I^\theta ,J^\varepsilon }| \le \tilde{\eta }_{k,k}$. $\square$

### Stabilizing two-parameter diagonal operators: the upper and lower diagonal conditions

For a diagonal operator $D:\mathcal {V}(\delta ^2)\rightarrow \mathcal {V}(\delta ^2)$ and a Haar-type vector $\tilde{h}$, $\tilde{k}$ we wish to define diagonal operators $D_{(\tilde{h},\cdot )}$ and $D_{(\cdot ,\tilde{k})}:\mathcal {V}(\delta )\rightarrow \mathcal {V}(\delta )$. For motivational purposes, we do so indirectly by first giving a more general way of reducing *D* to a one-parameter operator.

#### Notation 3.24

- For a linear operator $D:\mathcal {V}(\delta ^2)\rightarrow \mathcal {V}(\delta ^2)$ and $f^*,f\in \mathcal {V}(\delta )$ define $D_{(f^*,f)}:\mathcal {V}(\delta )\rightarrow \mathcal {V}(\delta )$ given by
  $$\begin{aligned} D_{(f^*,f)}(g) = \sum _{J\in \mathcal {D}}\frac{k_J}{|J|}\langle f^*\otimes k_J,D(f\otimes g)\rangle \end{aligned}$$
  If in particular, if $D:\mathcal {V}(\delta ^2)\rightarrow \mathcal {V}(\delta ^2)$ is diagonal with entries $(d_{I,J})_{I,J\in \mathcal {D}}$ then $D_{(f^*,f)}$ is diagonal as well with entries $\tilde{d}_J = \sum _{I\in \mathcal {D}}|I|^{-1}\langle f^*,h_I\rangle \langle h_I,f\rangle d_{I,J}$, $J\in \mathcal {D}$.
- For a diagonal linear operator $D:\mathcal {V}(\delta ^2)\rightarrow \mathcal {V}(\delta ^2)$ and $I\in \mathcal {D}$ take $f^* = h_I/|I|$ and $f = h_I$ and denote $D_{(I,\cdot )} = D_{(f^*,f)}$. This coincides with the definition of $D_{(I,\cdot )}$ in Remark 3.18 (a’).
- For a diagonal linear operator $D:\mathcal {V}(\delta ^2)\rightarrow \mathcal {V}(\delta ^2)$ and a Haar-type vector of the form $\tilde{h} = \sum _{I\in \mathcal {A}}\varepsilon _I h_I$, where, for some $m\in \mathbb {N}$, $\mathcal {A}\subset \mathcal {D}_{m}$ and $(\varepsilon _I)_{I\in \mathcal {A}}\in \{\pm 1\}^\mathcal {A}$, denote $D_{(\tilde{h},\cdot )} = D_{(f^*,f)}$, for $f^* = \tilde{h}/|\cup \mathcal {A}|$ and $f = \tilde{h}$. Then,
  $$\begin{aligned} D_{(\tilde{h},\cdot )} = |\mathcal {A}|^{-1}\sum _{I\in \mathcal {A}}D_{(I,\cdot )}. \end{aligned}$$
  The preceding definitions explain why the signs $(\varepsilon _I)_{I\in \mathcal {A}}$ don’t appear in the formula for $D_{(\tilde{h},\cdot )}$.
- For a diagonal linear operator $D:\mathcal {V}(\delta ^2)\rightarrow \mathcal {V}(\delta ^2)$ and a Haar-type vector of the form $\tilde{k} = \sum _{J\in \mathcal {D}}\theta _J\tilde{k}_J$, where, for some $n\in \mathbb {N}$, $\mathcal {B}\subset \mathcal {D}_n$ and $(\theta _J)_{J\in \mathcal {B}}\in \{\pm 1\}^\mathcal {B}$, denote
  $$\begin{aligned} D_{(\cdot ,\tilde{k})} = |\mathcal {B}|^{-1}\sum _{J\in \mathcal {B}}D_{(\cdot ,J)}, \end{aligned}$$
  where the $D_{(\cdot ,J)}$ were defined in Remark 3.18 (b’). Note that $D_{(\cdot ,\tilde{k})}$ could have also been defined indirectly, by taking $D^{(g^*,g)}(f) = \sum _{I\in \mathcal {D}}\frac{h_I}{|I|}\langle h_I\otimes g^*, D(f\otimes g)\rangle$ for appropriate $g^*$, *g* in $\mathcal {D}(\delta )$.

#### Lemma 3.25

Let $D:\mathcal {V}(\delta ^2)\rightarrow \mathcal {V}(\delta ^2)$ be an $\ell ^{\infty }$-bounded diagonal operator, $\tilde{H}=(\tilde{h}_I)_{I\in \mathcal {D}}$ and $\tilde{K}=( \tilde{k}_J)_{J\in \mathcal {D}}$ be faithful Haar systems relative to the frequencies $(m_i)_{i=0}^\infty$ and $(n_j)_{j=0}^\infty$ respectively and put $\tilde{D} = D|_{\tilde{H}\otimes \tilde{K}}$. Then, for every $I,J\in \mathcal {D}$,

3.15 $$\begin{aligned} D_{(\tilde{h}_I,\cdot )}|_{\tilde{K}} = \tilde{D}_{(I,\cdot )}\text { and }D_{(\cdot ,\tilde{k}_J)}|_{\tilde{H}} = \tilde{D}_{(\cdot ,J)}. \end{aligned}$$

Let also $$\eta = (\eta _{i,j})_{i,j=0}^\infty \subset (0,1)$$.

- If for all $j\in \mathbb {N}_0$ and $J\in \mathcal {D}_j$ the operator $D_{(\cdot ,\tilde{k}_J)}|_{\tilde{H}}$ is $(\eta _{i,j})_{i=0}^\infty$-stabilized from *j* on then $D|_{\tilde{H}\otimes \tilde{K}}$ satisfies the lower triangular condition.
- If for all $i\in \mathbb {N}_0$ and $I\in \mathcal {D}_i$ the operator $D_{(\tilde{h}_I,\cdot )}|_{\tilde{K}}$ is $(\eta _{i,j})_{j=0}^\infty$-stabilized from $i+1$ on then $D|_{\tilde{H}\otimes \tilde{K}}$ satisfies the upper triangular condition.

#### Proof

Note that items (a) and (b) follow directly from (3.15) and Remark 3.18. We show the first part of (3.15). Let $(d_{I,J})_{I,J\in \mathcal {D}}$ denote the entries of *D*. For all $i,j\in \mathbb {N}_0$, $I\in \mathcal {D}_i$, and $J\in \mathcal {D}_j$ write $\mathcal {A}_I = \{L\in \mathcal {D}_{m_i}:L\subset \textrm{supp}(\tilde{h}_I)\}$ and $\mathcal {B}_J = \{M\in \mathcal {D}_{n_j}:M\subset \textrm{supp}(\tilde{k}_J)\}$. Then, the operator $\tilde{D}$ has entries $(\tilde{d}_{I,J})_{I,J\in \mathcal {D}}$ with $\tilde{d}_{I,J} = \sum _{L\in \mathcal {A}_I} \frac{|L|}{|I|} \sum _{M\in \mathcal {B}_J} \frac{|M|}{|J|} d_{L,M}$, whenever $I\in \mathcal {D}_i$ and $J\in \mathcal {D}_j$.

Fix now $i\in \mathbb {N}$ and $I\in \mathcal {D}_i$. Then, $\tilde{D}_{(I,\cdot )}$ is the diagonal operator with entries $(\tilde{d}_{I,J})_{J\in \mathcal {D}}$. By definition, $\tilde{D}_{(\tilde{h}_I,\cdot )}$ is the diagonal operator that has entries $\bigl (\sum _{L\in \mathcal {A}_I} \frac{|L|}{|I|} d_{L,J}\bigr )_{J\in \mathcal {D}}$. We then deduce that $D_{(\tilde{h}_I,\cdot )}|_{\tilde{K}}$ is the diagonal operator such that, for each $J\in \mathcal {D}_j$, has entry $c_J = \sum _{M\in \mathcal {B}_J} \frac{|M|}{|J|}\sum _{L\in \mathcal {A}_I} \frac{|L|}{|I|} d_{L,M}$, which coincides with $\tilde{d}_{I,J}$, the corresponding entry of $\tilde{D}_{(I,\cdot )}$.$\square$

#### Proposition 3.26

Let $D:\mathcal {V}(\delta ^2)\rightarrow \mathcal {V}(\delta ^2)$ be an $\ell ^{\infty }$-bounded diagonal operator. Then, for every infinite subsets *M*, *N* of $\mathbb {N}$ and $\eta =(\eta _{i,j} )_{i,j=0}^\infty \in (0,1)^{\mathbb {N}^2}$ there exist faithful Haar systems $\tilde{H}=(\tilde{h}_I:I\in \mathcal {D})$ and $\tilde{K}=( \tilde{k}_J:J\in \mathcal {D})$ relative to frequencies $(m_i)_{i=0}^\infty \subset M$ and $(n_j)_{j=0}^\infty \subset N$ respectively, such that, for $i\ge 0$, $1\le n_i< m_i < n_{i+1}$, and $D|_{\tilde{H}\otimes \tilde{K}}$ satisfies the lower and upper triangular conditions for $\eta$.

#### Proof

We proceed with an application of the game in Proposition 3.8. There will be two sets of the game being played simultaneously, one set $\textrm{G}_1$ for defining $\tilde{H} = (\tilde{h}_I:I\in \mathcal {D})$ and another set $\textrm{G}_2$ for defining $\tilde{K}=( \tilde{k}_J:J\in \mathcal {D})$. In both, we assume the role of Player I and let Player II follow their winning strategy. As Player I, in each round *k* and for $\textrm{G}_1$ we will choose an appropriate error, a finite collection of $\ell ^{\infty }$-bounded diagonal operators $\mathcal {F}_k^{1}$, and an infinite sets $M^1_k\subset N^1_{(k-1)}$. Then, Player II will choose $N^1_{(k)}\subset M^1_k$, $n_k=\min ( N^1_{(k)})$, and $(\tilde{k}_J)_{J\in \mathcal {D}_{{k}}}$ in $\langle \{k_L:L\in \mathcal {D}_{n_k}\}\rangle$. Then we play the *k*’th round of $\textrm{G}_2$ by choosing an error, $\mathcal {F}_k^{2}$, $M_k^2\subset N^2_{(k-1)}$ and let Player II chose $N^2_{(k)}\subset M^2_k$, $m_k = \min (N^2_{(k)})$, and $(\tilde{h}_I)_{I\in \mathcal {D}_{k}}$ in $\langle \{h_K:K\in \mathcal {D}_{m_k}\}\rangle$.

In the zeroth round of $\textrm{G}_1$ we choose $M^1_0 = N$, but our choice of operators or error does not really matter. So we let Player II choose $n_0$ and $\tilde{k}_{[0,1)}$. In the zeroth round of $G_2$, we choose error $\eta _0' = \eta _{0,0}$, $\mathcal {F}^{2}_0 = \{D_{(\cdot ,\tilde{k}_{[0,1)})}\}$, while our choice of $M^2_{0}$ is a subset of *M* with $\min (M^2_0) > n_0$. Player II then chooses $N^2_{(0)}\subset M^2_0$, lets $m_0 = \min (N^2_{(0)})> n_0$, and chooses $\tilde{h}_{[0,1)}$.

Assume that we have played rounds $0,1,\ldots ,k-1$. In round *k* of $\textrm{G}_1$, choose error $\eta _k' = \min _{i,j \le k}\eta _{i,j}$, $\mathcal {F}_k^1 = \{D_{(\tilde{h}_I,\cdot )}:I\in \mathcal {D}_{k-1}\}$, and $M^1_k\subset N^1_{(k-1)}$ such that $m_{k-1}<\min (M^1_k)$. Then, Player II chooses $N^1_{(k)}\subset M^1_k$, lets $n_k = \min (N^1_{(k)}) > m_{k-1}$, and chooses $(\tilde{k}_J)_{J\in \mathcal {D}_k}$ in $\langle \{k_L:L\in \mathcal {D}_{n_k}\}\rangle$. In round *k* of $\textrm{G}_2$, choose error $\eta _k' = \min _{i,j\le k}\eta _{i,j}$, $\mathcal {F}_k^2 = \{D_{(\cdot ,\tilde{k}_J)}:J\in \mathcal {D}_{k}\}$, and $M^2_k\subset N^2_{(k-1)}$ such that $n_{k}<\min (M^2_k)$. Then, Player II chooses $N^2_{(k)}\subset M^2_k$, lets $m_k = \min (N^1_{(k)}) > n_{k}$, and chooses $(\tilde{h}_I)_{I\in \mathcal {D}_k}$ in $\langle \{h_K:K\in \mathcal {D}_{m_k}\}\rangle$.

Let $\tilde{H} = (\tilde{h}_I)_{I\in \mathcal {D}}$ and $\tilde{K} = (\tilde{k}_J)_{J\in \mathcal {D}}$ and $\eta ' = (\eta '_k)_{k=0}^\infty$. Then, for every $k\in \mathbb {N}$ and for every $I\in \mathcal {D}_{k-1}$, $D_{(\tilde{h}_I,\cdot )}|_{\tilde{H}}$ is $\eta '$-stabilized from *k* on, thus, by Lemma 3.25 (b), $D_{\tilde{H}\otimes \tilde{K}}$ satisfies the upper triangular condition. Also, for every $k\in \mathbb {N}_0$, $J\in \mathcal {D}_k$, $D_{(\cdot ,\tilde{k}_J)}|_{\tilde{H}}$ is $\eta '$-stabilized from *k* on. By Lemma 3.25 (a) $D_{\tilde{H}\otimes \tilde{K}}$ satisfies the lower triangular condition. $\square$

### Stabilizing two-parameter diagonal operators: the diagonal and superdiagonal conditions

In order to obtain faithful Haar systems $\tilde{H}\otimes \tilde{K}=(\tilde{h}_I\otimes \tilde{k}_J: I,J\in \mathcal {D})$ for which $D|_{\tilde{H}\otimes \tilde{K}}$ satisfies the “diagonal conditions” (c) and (d) of Definition 3.17, we will need the 2-parameter version of the probabilistic Lemma 3.9.

#### Lemma 3.27

Let $D:\mathcal {V}(\delta ^2)\rightarrow \mathcal {V}(\delta ^2)$ be an $\ell ^{\infty }$-bounded operator. Let $i<k$, $j<l$ be in $\mathbb {N}_0$ and let $\Gamma \in \sigma (\mathcal {D}_i)$ and $\Delta \in \sigma (\mathcal {D}_j)$. For $\varepsilon =(\varepsilon _{I}: I\in \mathcal {D}_i, I\subset \Gamma )$ and $\theta =(\theta _J: J\in \mathcal {D}_j, J\subset \Delta )$, $\omega =\pm 1$ and $\xi =\pm 1$ put

$$\begin{aligned} \Gamma ^\omega (\varepsilon ) = \left\{ \sum _{\begin{array}{c} I\in \mathcal {D}_i\\ I\subset \Gamma \end{array}} \varepsilon _{I}h_I=\omega \right\} \qquad \text {and}\qquad \Delta ^\xi (\theta ) =\left\{ \sum _{\begin{array}{c} J\in \mathcal {D}_J\\ J\subset \Delta \end{array}}\theta _J k_J=\xi \right\} . \end{aligned}$$

Let $\mathcal {E}=(\mathcal {E}_{I}: I\in \mathcal {D}_i, I\subset \Gamma )$, $\Theta =(\Theta _J: J\in \mathcal {D}_j, J\subset \Delta )$ be two independent Rademacher families on some probability space and define the random variable

$$\begin{aligned} X_{\omega ,\xi } = \sum _{\begin{array}{c} K\in \mathcal {D}_k\\ K\subset \Gamma ^\omega (\mathcal {E}) \end{array}} \frac{|K|}{|\Gamma ^\omega (\mathcal {E})|} \sum _{\begin{array}{c} L\in \mathcal {D}_l\\ L\subset \Delta ^\xi (\Theta ) \end{array}} \frac{|L|}{|\Delta ^\xi (\Theta )|} d_{K,L}. \end{aligned}$$

Then it follows, for the expectation and variance of $X_{\omega ,\xi }$,

$$\begin{aligned} {{\,\mathrm{\mathbb {E}}\,}}(X_{\omega ,\xi }) = \sum _{\begin{array}{c} K\in \mathcal {D}_k\\ K\subset \Gamma \end{array}} \frac{|K|}{|\Gamma |} \sum _{\begin{array}{c} L\in \mathcal {D}_l\\ L\subset \Delta \end{array}} \frac{|L|}{|\Delta |} d_{J,L} \qquad \text {and}\qquad {{\,\mathrm{\mathbb {V}}\,}}(X_{\omega ,\xi }) \le 4 \Vert D\Vert ^2_\infty \left( \frac{2^{-i}}{|\Gamma |} + \frac{2^{-j}}{|\Delta |} \right) . \end{aligned}$$

#### Proof

Without loss of generality, we assume $\omega =\xi =1$ and write $X = X_{\omega ,\xi }$. It follows that $|\Gamma ^+(\varepsilon )|=\frac{|\Gamma |}{2}$ and $|\Delta ^+(\theta )|=\frac{|\Delta |}{2}$ for all $\varepsilon =(\varepsilon _I: I\in \mathcal {D}_i, I\subset \Gamma )$ and $\theta =(\theta _J: J\in \mathcal {D}_j, J\subset \Delta )$. For $K\in \mathcal {D}_k$ and $L\in \mathcal {D}_l$ we introduce two auxiliary random variables $Y_{K}$ and $Z_{L}$. Let $I\in \mathcal {D}_i$, $J\in \mathcal {D}_j$ with $K\subset I$ and $L\subset J$. Define

$$\begin{aligned} Y_K&= \langle |K|^{-1}\chi _K,\chi _{I^{\mathcal {E}_I}}\rangle = {\left\{ \begin{array}{ll} 1&{}\quad \text {if } K\subset \Gamma ^+(\mathcal {E}),\\ 0&{}\quad \text {if } K\cap \Gamma ^+(\mathcal {E}) = \emptyset , \end{array}\right. }\\ Z_L&= \langle |L|^{-1}\chi _L,\chi _{J^{\Theta _J}}\rangle = {\left\{ \begin{array}{ll} 1&{}\quad \text {if } L\subset \Delta ^+(\Theta ),\\ 0&{}\quad \text {if } L\cap \Delta ^+(\Theta ) = \emptyset , \end{array}\right. } \end{aligned}$$

which are independent and ${{\,\mathrm{\mathbb {E}}\,}}(Y_K) = {{\,\mathrm{\mathbb {E}}\,}}(Z_L) = 1/2$.

Thus, we compute

$$\begin{aligned} {{\,\mathrm{\mathbb {E}}\,}}(X)&= \sum _{\begin{array}{c} I\in \mathcal {D}_{i}\\ I\subset \Gamma \end{array}} \frac{2|I|}{|\Gamma |} \sum _{\begin{array}{c} J\in \mathcal {D}_{j}\\ J\subset \Delta \end{array}} \frac{2|J|}{|\Delta |} {{\,\mathrm{\mathbb {E}}\,}}\left( \sum _{\begin{array}{c} K\in \mathcal {D}_{k}\\ K\subset I\cap \Gamma ^+(\mathcal {E}) \end{array}} \frac{|K|}{|I|} \sum _{\begin{array}{c} L\in \mathcal {D}_{l}\\ L\subset J\cap \Delta ^+(\Theta ) \end{array}} \frac{|L|}{|J|} d_{J,L} \right) \\&= 4\sum _{\begin{array}{c} I\in \mathcal {D}_{i}\\ I\subset \Gamma \end{array}} \frac{|I|}{|\Gamma |} \sum _{\begin{array}{c} J\in \mathcal {D}_{j}\\ J\subset \Delta \end{array}} \frac{|J|}{|\Delta |} \sum _{K\in \mathcal {D}_k, K\subset I} \frac{|K|}{|I|}\sum _{L\in \mathcal {D}_l, L\subset J} \frac{|L|}{|J|} d_{K,L}{{\,\mathrm{\mathbb {E}}\,}}(Y_KZ_L)\\&= \sum _{\begin{array}{c} K\in \mathcal {D}_k\\ K\subset \Gamma \end{array}} \frac{|K|}{|\Gamma |} \sum _{\begin{array}{c} L\in \mathcal {D}_l\\ L\subset \Delta \end{array}} \frac{|L|}{|\Delta |} d_{K,L}. \end{aligned}$$

Define for $I\in \mathcal {D}_i$, with $I\subset \Gamma$, and $J\in \mathcal {D}_j$, with $J\subset \Delta$, the random variable

$$\begin{aligned} X(I,J)&= 4 \sum _{\begin{array}{c} K\in \mathcal {D}_k, \\ K\subset I\cap \Gamma ^+(\mathcal {E}) \end{array}}\frac{|K|}{|\Gamma |} \sum _{\begin{array}{c} L\in \mathcal {D}_l\\ L\subset J\cap \Delta ^+(\Theta ) \end{array}} \frac{|L|}{|\Delta |} d_{K,L}\\&= 4\sum _{K\in \mathcal {D}_k, K\subset I} \frac{|K|}{|\Gamma |} \sum _{L\in \mathcal {D}_l, L\subset J} \frac{|L|}{|\Delta |} d_{K,L}Y_KZ_L \end{aligned}$$

which only depends on the random variables $\mathcal {E}_I$ and $\Theta _J$, by the definition of $Y_K$, $Z_L$. Therefore, if $I\ne I'\in \mathcal {D}_i$, $I,I'\subset \Gamma$ and $J \ne J'\in \mathcal {D}_j$, $J,J'\subset \Delta$ then

3.16 $$\begin{aligned} \textrm{cov}(X(I,J),X(I',J')) = {{\,\mathrm{\mathbb {E}}\,}}((X(I,J)-{{\,\mathrm{\mathbb {E}}\,}}(X(I,J)))(X(I',J')-{{\,\mathrm{\mathbb {E}}\,}}(X(I',J')))) = 0. \end{aligned}$$

Furthermore, since

$$\begin{aligned} |\{K\in \mathcal {D}_k: K\subset I\cap \Gamma ^+(\mathcal {E})\}| = 2^l 2^{-i}/2 \quad \text {and}\quad |\{L\in \mathcal {D}_l: L\subset J\cap \Delta ^+(\Theta )\}| = 2^l2^{-j}/2, \end{aligned}$$

we observe that

$$\begin{aligned} |X(I,J)|\le 2^{-i-j}\frac{1}{|\Gamma |\cdot |\Delta | }\cdot \Vert D\Vert _\infty . \end{aligned}$$

and therefore, for arbitrary $I,I'\in \mathcal {D}_i$, $I,I'\subset \Gamma$ and $J,J'\in \mathcal {D}_j$, $J,J'\subset \Delta$ we have

3.17 $$\begin{aligned} \bigl |\textrm{cov}(X(I,J),X(I',J'))\bigr | \le 4\frac{2^{-2i-2j}}{|\Gamma |^2\cdot |\Delta |^2}\Vert D\Vert _\infty ^2. \end{aligned}$$

Denote

$$\begin{aligned} \mathscr {A} = \left\{ (I,I',J,J')\in \mathcal {D}_i\times \mathcal {D}_i\times \mathcal {D}_j\times \mathcal {D}_j: I,I'\subset \Gamma , J,J'\subset \Delta \text { and }I = I'\text { or }J = J'\right\} . \end{aligned}$$

and note that $\#\mathscr {A} = |\Gamma |2^i\cdot |\Delta |2^j(|\Gamma |2^i + |\Delta |2^j - 1)$. To conclude, we estimate

$$\begin{aligned} {{\,\mathrm{\mathbb {V}}\,}}(X)&= {{\,\mathrm{\mathbb {V}}\,}}\left( \sum _{\begin{array}{c} I\in \mathcal {D}_i\\ I\subset \Gamma \end{array}}\sum _{\begin{array}{c} J\in \mathcal {D}_j \\ J\subset \Delta \end{array}} X(I,J) \ \right) = \sum _{\begin{array}{c} I,I'\in \mathcal {D}_i\\ I,I'\subset \Gamma \end{array}} \sum _{\begin{array}{c} J,J'\in \mathcal {D}_j \\ J,J'\subset \Delta \end{array}}\textrm{cov}\Big (X(I,J),X(I',J')\Big )\\&\le \#\mathscr {A} \cdot 4\frac{2^{-2i-2j}}{|\Gamma |^2\cdot |\Delta |^2}\Vert D\Vert _\infty ^2 = 4\Vert D\Vert _\infty ^2\frac{2^{-i-j}}{|\Gamma |\cdot |\Delta |} \Big (|\Gamma |2^i + |\Delta |2^j - 1\Big )\\&< 4 \Vert D\Vert ^2_\infty \left( \frac{2^{-i}}{|\Gamma |} + \frac{2^{-j}}{|\Delta |} \right) . \end{aligned}$$

$$\square $$

#### Remark 3.28

Under the assumptions of Lemma 3.27 put

$$\begin{aligned} d_{\Gamma ,\Delta }^{i,j}&= \sum _{\begin{array}{c} I\in \mathcal {D}_i\\ I\subset \Gamma \end{array}}\frac{|I|}{|\Gamma |} \sum _{\begin{array}{c} J\in \mathcal {D}_k\\ J\subset \Delta \end{array}} \frac{|J|}{|\Delta |} d_{I,J}\\ d_{\Gamma ,\Delta }^{k,l}&= \sum _{\begin{array}{c} K\in \mathcal {D}_k\\ K\subset \Gamma \end{array}} \frac{|K|}{|\Gamma |} \sum _{\begin{array}{c} L\in \mathcal {D}_l\\ L\subset \Delta \end{array}} \frac{|L|}{|\Delta |} d_{K,L} = {{\,\mathrm{\mathbb {E}}\,}}(X_{\omega ,\xi }). \end{aligned}$$

Then an argument similar to Remark 3.10 yields that, for any $\delta >0$,

$$\begin{aligned} {{\,\mathrm{\mathbb {E}}\,}}\left( \max _{\omega ,\xi =\pm 1}\left| X_{\omega ,\xi } - d_{\Gamma ,\Delta }^{i,j} \right| \le \delta + \left| d_{\Gamma ,\Delta }^{i,j} - d_{\Gamma ,\Delta }^{k,l} \right| \right) \ge 1 - \frac{16}{\delta ^2}\Vert D\Vert ^2_\infty \left( \frac{2^{-i}}{|\Gamma |} + \frac{2^{-j}}{|\Delta |}\right) . \end{aligned}$$

Therefore, if $(\Gamma _\alpha )_{\alpha \in \mathcal {A}}$ are disjoint subsets in $\sigma (\mathcal {D}_{i})$ and $(\Delta _\beta )_{\beta \in \mathcal {B}}$ are disjoint subsets in $\sigma (\mathcal {D}_j)$ and

$$\begin{aligned} 2^{-i}\left( \sum _{\alpha \in \mathcal {A}}\frac{|\mathcal {B}|}{|\Gamma _\alpha |}\right) + 2^{-j}\left( \sum _{\beta \in \mathcal {B}}\frac{|\mathcal {A}|}{|\Delta _\beta |}\right) < \frac{\delta ^2}{16\Vert D\Vert _\infty ^{2}}, \end{aligned}$$

there exist choices of signs $(\varepsilon _I:I\in \mathcal {D}_i, I\subset \Gamma _\alpha )$, $\alpha \in \mathcal {A}$, and $(\theta _J:J\in \mathcal {D}_j,J\subset \Delta _\beta )$, $\beta \in \mathcal {B}$, such that for any $(\alpha ,\beta )\in \mathcal {A}\times \mathcal {B}$ and $\omega ,\xi \in \{\pm 1\}$ if we put

$$\begin{aligned} \Gamma _\alpha ^\omega = \left\{ \sum _{\begin{array}{c} I\in \mathcal {D}_i\\ I\subset \Gamma _\alpha \end{array}} \varepsilon _Ih_I=\omega \right\} , \qquad \Delta _\beta ^\xi = \left\{ \sum _{\begin{array}{c} J\in \mathcal {D}_j\\ J\subset \Delta _\beta \end{array}}\theta _J k_J=\xi \right\} , \end{aligned}$$

it follows that

3.18 $$\begin{aligned} \left| \sum _{\begin{array}{c} K\in \mathcal {D}_k\\ K\subset \Gamma _\alpha ^\omega \end{array}} \frac{|K|}{|\Gamma _{\alpha }^{\omega }|} \sum _{\begin{array}{c} L\in \mathcal {D}_l\\ L\subset \Delta _\beta ^\xi \end{array}} \frac{|L|}{|\Delta _{\beta }^{\xi }|} d_{K,L} - d^{i,j}_{\Gamma _\alpha ,\Delta _\beta } \right| \le \delta + \left| d_{\Gamma _\alpha ,\Delta _\beta }^{i,j} - d_{\Gamma _\alpha ,\Delta _\beta }^{k,l} \right| . \end{aligned}$$

#### Remark 3.29

A straightforward modification of the proof of Ramsey’s Theorem yields the following. Let *M*, *N* be infinite subset of $\mathbb {N}$ and assume that we are given a finite coloring of the set

$$\begin{aligned}{}[M,N] := \{(m,n):m<n\text { with }m\in M, n\in N\}. \end{aligned}$$

Then, there exist infinite $M'\subset M$, $N'\subset N$ such that $[M',N']$ is monochromatic.

#### Proposition 3.30

Let $D:\mathcal {V}(\delta ^2)\rightarrow \mathcal {V}(\delta ^2)$ be an $\ell ^{\infty }$-bounded diagonal operator, *M*, *N* be infinite subsets of $\mathbb {N}$, $\eta = (\eta _{i,j})_{i,j=0}^\infty \subset (0,1)$. Then, there exist faithful Haar systems $\tilde{H} = (h_I)_{I\in \mathcal {D}}$, $\tilde{K} = (k_J)_{J\in \mathcal {D}}$ relative to frequencies $(m_i)_{i=0}^\infty \subset M$ and $(n_j)_{i=0}^\infty \subset N$ with $1\le n_i< m_i<n_{i+1}$, for $i\ge 0$, such that $D|_{\tilde{H}\otimes \tilde{K}}$ satisfies the superdiagonal condition for $\eta$.

#### Proof

For each $(\Gamma ,\Delta )\in \sigma (\mathcal {D}_{i_0})\times \sigma (\mathcal {D}_{j_0})$ and $i\ge i_0$, $j\ge j_0$ define

$$\begin{aligned} d^{i,j}_{\Gamma ,\Delta } = \sum _{\begin{array}{c} I\in \mathcal {D}_i\\ I\subset \Gamma \end{array}} \frac{|I|}{|\Gamma |} \sum _{\begin{array}{c} J\in \mathcal {D}_j\\ J\subset \Delta \end{array}} \frac{|J|}{|\Delta |} d_{I,J}. \end{aligned}$$

Take $n_0\in N$ arbitrary and $\tilde{k}_{[0,1)} = \sum _{L\in \mathcal {D}_{n_0}}k_L$. We will perform an induction on $k\in \mathbb {N}_0$ and in each step we will choose $m_k$, $n_{k+1}$, $M_k$, $N_{k+1}$, $(\tilde{h}_I)_{I\in \mathcal {D}_k}$, and $(\tilde{k}_J)_{J\in \mathcal {D}_{k+1}}$ such that the following are satisfied.

- $$M\supset M_0\supset \cdots \supset M_{k}$$ and $$N\supset N_0\supset \cdots \supset N_{k+1}$$.
- $$n_0<m_0<n_1<\cdots<m_k<n_{k+1}$$ and $$m_k =\min (M_k)$$, $$n_{k+1} = \min (N_{k+1})$$.
- If $$k = 0$$, for $$I = [0,1)$$, there are $$(\varepsilon _K:K\in \mathcal {D}_{m_0})\in \{\pm 1\}^{\mathcal {D}_{m_0}}$$ such that
  $$\begin{aligned} \tilde{h}_{[0,1)} = \sum _{K\in \mathcal {D}_{m_0}}\varepsilon _Kh_K \end{aligned}$$
  whereas if $k\ge 1$, for $I\in \mathcal {D}_{k}$, with $I = \pi (I)^\omega$, for some $\omega \in \{\pm 1\}$, and $\Gamma _I = \{\tilde{h}_{\pi (I)} = \omega \}$, then there are $(\varepsilon _K:K\in \mathcal {D}_{m_k},\;K\subset \Gamma _I)$ in $\{\pm 1\}$ such that
  $$\begin{aligned} \tilde{h}_I = \sum _{\begin{array}{c} K\in \mathcal {D}_{m_k}\\ K\subset \Gamma _I \end{array}}\varepsilon _Kh_K. \end{aligned}$$
- For $J\in \mathcal {D}_{k+1}$ with $J = \pi (I)^\xi$, for some $\xi \in \{\pm 1\}$, and $\Delta _J = \{\tilde{k}_{\pi (J)} = \xi \}$, there are $\{\theta _L:L\in \mathcal {D}_{n_{k+1}},\;L\subset \Delta _J\}$ in $\{\pm 1\}$ such that
  $$\begin{aligned} \tilde{k}_J = \sum _{\begin{array}{c} L\in \mathcal {D}_{n_{k+1}}\\ L\subset \Delta _J \end{array}}\theta _Lk_L. \end{aligned}$$
- For $I\in \mathcal {D}_{k}$, $J\in \mathcal {D}_{k+1}$, $\omega ,\xi \in \{\pm 1\}$, and for any $(i,j)\in [M_k\setminus \{m_k\},N_{k+1}\setminus \{n_{k+1}\}]$,
  $$\begin{aligned} \left| d^{m_{k},n_{k+1}}_{\Gamma _I,\Delta _J} - d^{i,j}_{\{\tilde{h}_I=\omega \},\{\tilde{k}_J=\xi \}}\right| < \eta _{k,k+1}. \end{aligned}$$

Let us quickly observe that these conditions yield the desired conclusion. Indeed, by (c) and (d), $\tilde{H} = (\tilde{h}_I)_{I\in \mathcal {D}}$ and $\tilde{K} = (\tilde{k}_L)_{L\in \mathcal {D}}$ are faithful Haar systems relative to the frequencies $(m_i)_{i=0}^\infty$ and $(n_j)_{j=0}^\infty$ respectively and that $D|_{\tilde{H}\otimes \tilde{K}}$ is the diagonal operator with entries $((d^{m_i,n_j}_{I,J})_{I\in \mathcal {D}_{i},J\in \mathcal {D}_j})_{i,j=0}^\infty$. Finally, by (e) and taking $i = m_{k+1}$, $l = n_{k+2}$, we obtain that for all $k\in \mathbb {N}_0$, $I\in \mathcal {D}_{k}$, $J\in \mathcal {D}_{k+1}$, and $\omega ,\xi \in \{\pm 1\}$, $|\tilde{d}_{I,J} - \tilde{d}_{I^\omega ,L^\xi }| < \eta _{k,k+1}$.

We will only describe the basis step, as it is the same as the general one. Put $\Delta _{[0,1/2)} = \{\tilde{k}_{[0,1)}=1\}$ and $\Delta _{[1/2,1)} = \{\tilde{k}_{[0,1)}=-1\}$. Using Remark 3.29, pass to infinite $N'_1\subset N$, and $M'_0\subset M$ such that for any (*i*, *j*), $(l,m)\in [M_0',N_1']$ and $J \in \mathcal {D}_1$ we have

$$\begin{aligned} |d^{i,j}_{[0,1),\Delta _J} - d^{l,m}_{[0,1),\Delta _J}| < \eta _{0,1}/2. \end{aligned}$$

Pick $m_0 \in M$ and $n_1 \in N$ sufficiently large to have

$$\begin{aligned} 2^{-m_0}\cdot 2+2^{-n_1}\cdot 2 < \frac{1}{16} \Vert D\Vert _\infty ^{-2} \big (\eta _{0,1}/2\big )^2. \end{aligned}$$

Let $M_0'' = (m_0,\infty )\cap M_0'$ and $N_1'' = (n_1,\infty )\cap N_1'$. By Remark 3.28, for any $(k,l)\in [M_0'',N_1'']$ we can find

$$\begin{aligned} \varepsilon ^{(k,l)} = (\varepsilon ^{(k,l)}_K:K\in \mathcal {D}_{m_0},K\subset [0,1)),\text { and }\theta ^{(k,l)} = (\theta ^{(k,l)}_L:L\in \mathcal {D}_{n_1},L\subset \Delta _J, J\in \mathcal {D}_1) \end{aligned}$$

such that for $J\in \mathcal {D}_1$ and $\omega ,\xi \in \{\pm 1\}$, if we put

$$\begin{aligned} \Gamma _{[0,1)}^\omega (\varepsilon ^{(k,l)}) = \left\{ \sum _{\begin{array}{c} K\in \mathcal {D}_{m_0}\\ K\subset [0,1) \end{array}}\varepsilon ^{(k,l)}_Lh_L = \omega \right\} \quad \text {and}\quad \Delta _{J}^\xi (\theta ^{(k,l)}) = \left\{ \sum _{\begin{array}{c} L\in \mathcal {D}_{n_1}\\ K\subset \Delta _J \end{array}}\theta ^{(k,l)}_L k_L = \xi \right\} \end{aligned}$$

then

3.19 $$\begin{aligned} \big | d^{m_1,n_0}_{\Gamma _I,[0,1)} - d^{j,l}_{\Gamma _I^\varepsilon (\theta _{(l,j)}), \Lambda _{[0,1)}^\delta (\phi _{(l,j)})} \big |< \frac{\eta _{1,0}}{2} + \big |d^{m_1,n_0}_{\Gamma _I,[0,1)} - d^{j,l}_{\Gamma _I,[0,1)}\big | < \eta _{1,0}. \end{aligned}$$

By Remark 3.29, we may choose infinite $\tilde{M}_0\subset M_0''$ and $\tilde{N}_1\subset N_1''$ such that on $[\tilde{M}_0,\tilde{N}_1]$, $\varepsilon ^{(k,l)}$ and $\theta ^{(k,l)}$ are constant and equal to $\varepsilon$ and $\theta$. Put $M_0 = \{m_0\}\cup \tilde{M}_0$, $N_1 = \{n_1\}\cup \tilde{N}_1$, $$\tilde{h}_{[0,1)} = \sum _{\begin{array}{c} K\in \mathcal {D}_{m_0}\\ K\subset [0,1) \end{array}}\varepsilon _Lh_L$$, and for $J\in \mathcal {D}_1$, $$\tilde{k}_J = \sum _{\begin{array}{c} L\in \mathcal {D}_{n_1}\\ J\subset \Delta _J \end{array}}\theta _Jk_J$$. Then, all desired properties are satisfied.$\square$

The proof of the following proposition is performed along the same lines as the proof of Proposition 3.30. The important difference is that in each inductive step *k* one works with $n_k$, $m_k$ (instead of $m_k$, $n_{k+1}$).

#### Proposition 3.31

Let $D:\mathcal {V}(\delta ^2)\rightarrow \mathcal {V}(\delta ^2)$ be an $\ell ^{\infty }$-bounded diagonal operator, *M*, *N* be infinite subsets of $\mathbb {N}$, $\eta = (\eta _{i,j})_{i,j=0}^\infty \subset (0,1)$. Then, there exist faithful Haar systems $\tilde{H} = (h_I)_{I\in \mathcal {D}}$, $\tilde{K} = (k_J)_{J\in \mathcal {D}}$ relative to frequencies $(m_i)_{i=0}^\infty \subset M$ and $(n_i)_{i=0}^\infty \subset N$, such that $D|_{\tilde{H}\otimes \tilde{K}}$ satisfies the diagonal condition for $\eta$.

#### Proposition 3.32

Let $D:\mathcal {V}(\delta ^2)\rightarrow \mathcal {V}(\delta ^2)$ be an $\ell ^{\infty }$-bounded diagonal operator, *M*, *N* be infinite subsets of $\mathbb {N}$, $\delta > 0$. Then, there exist faithful Haar systems $\tilde{H} = (h_I)_{I\in \mathcal {D}}$, $\tilde{K} = (k_J)_{J\in \mathcal {D}}$ relative to frequencies $(m_i)_{i=0}^\infty \subset M$ and $(n_j)_{j=0}^\infty \subset N$, such that $D|_{\tilde{H}\otimes \tilde{K}}$ satisfies the balancing condition for $\delta$.

#### Proof

Fix $1\le n_0< m_0 < n_1$, such that $m_0\in M$, $n_0,n_1\in N$, and

$$\begin{aligned} 2^{-n_0} < \frac{\delta ^2}{8\Vert D\Vert _\infty ^2}. \end{aligned}$$

Define $\tilde{h}_{[0,1)} = \sum _{K\in \mathcal {D}_{m_0}}h_K$, for $L\in \mathcal {D}_{n_1}$ let

$$\begin{aligned} D_L = \sum _{K\in \mathcal {D}_{m_0}}|K|d_{K,L}, \end{aligned}$$

and note that $|D_J|\le \Vert D\Vert _\infty$.

For $$\theta = (\theta _L)_{L\in \mathcal {D}_{n_0}}\in \{\pm 1\}^{\mathcal {D}_{n_0}}$$ define

$$\begin{aligned} \tilde{k}_{[0,1)}^\theta&= \sum _{L\in \mathcal {D}_{n_0}}\theta _Lk_L,\\ \Delta _{[0,1/2)}(\theta )&= \{\tilde{k}_{[0,1)}^\theta = 1\},\quad \quad \tilde{k}_{[0,1/2)}^\theta = \sum _{\begin{array}{c} L\in \mathcal {D}_{n_1}\\ K\subset \Delta _{[0,1/2)}(\theta ) \end{array}}k_L,\\ \Delta _{[1/2,1)}(\theta )&= \{\tilde{k}_{[0,1)}^\theta = -1\}, \quad \quad \tilde{k}_{[1/2,1)}^\theta = \sum _{\begin{array}{c} L\in \mathcal {D}_{n_1}\\ L\subset \Delta _{[1/2,1)}(\theta ) \end{array}}k_L,\\ d_{[0,1),[0,1/2)}(\theta )&= 2\cdot \sum _{\begin{array}{c} K\in \mathcal {D}_{m_0}\\ K\subset [0,1) \end{array}} |K| \sum _{\begin{array}{c} L\in \mathcal {D}_{n_1}\\ L\subset \Delta _{[0,1/2)} \end{array}} |L| d_{K,L} = 2\cdot \sum _{\begin{array}{c} L\in \mathcal {D}_{n_1}\\ L\subset \Delta _{[0,1/2)}(\theta ) \end{array}} |L| D_L,\text { and}\\ d_{[0,1),[0,1/2)}(\theta )&= 2\cdot \sum _{\begin{array}{c} K\in \mathcal {D}_{m_0}\\ K\subset [0,1) \end{array}} |K| \sum _{\begin{array}{c} L\in \mathcal {D}_{n_1}\\ L\subset \Delta _{[1/2,1)} \end{array}} |L| d_{K,L} = 2\cdot \sum _{\begin{array}{c} L\in \mathcal {D}_{n_1}\\ L\subset \Delta _{[1/2,1)}(\theta ) \end{array}} |L| D_L. \end{aligned}$$

The goal is to pick $\theta$ such that $|d_{[0,1),[0,1/2)}(\theta ) - d_{[0,1),[1/2,1)}(\theta )| < \delta$. By Lemma 3.9, we obtain

$$\begin{aligned} {{\,\mathrm{\mathbb {E}}\,}}\big (d_{[0,1),[0,1/2)}(\theta )\big ) = {{\,\mathrm{\mathbb {E}}\,}}\big (d_{[0,1),[1/2,1)}(\theta )\big ) = \sum _{K\in \mathcal {D}_{m_0}} |K| \sum _{L\in \mathcal {D}_{n_1}} |L| d_{K,L} \end{aligned}$$

as well as

$$\begin{aligned} {{\,\mathrm{\mathbb {V}}\,}}\big (d_{[0,1),[0,1/2)}(\theta )\big )\vee {{\,\mathrm{\mathbb {V}}\,}}\big (d_{[0,1),[1/2,1)}(\theta )\big ) \le 2^{-n_0}\max _{L\in \mathcal {D}_{n_1}}|D_L|^2 \le 2^{-n_0}\Vert D\Vert _\infty ^2. \end{aligned}$$

The choice of $n_0$ and Chebyshev’s inequality yield the desired $\theta$. Put $\tilde{k}_{[0,1)} = \tilde{k}_{[0,1)}^\theta$, $\tilde{k}_{[0,1/2)} = \tilde{k}_{[0,1/2)}^\theta$, and $\tilde{k}_{[1/2,1)} = \tilde{k}_{[1/2,1)}^\theta$. The choice of the remaining components of $\tilde{H}$, $\tilde{K}$ only needs to respect the restriction $n_k<m_k<n_{k+1}$ and $m_k\in M$, $n_k\in N$.$\square$

#### Proof of Theorem 3.20

Let $D:\mathcal {V}(\delta ^2)\rightarrow \mathcal {V}(\delta ^2)$ be an $\ell ^{\infty }$-bounded diagonal operator and denote

$$\begin{aligned} \lambda (D)&= \lim _{n\rightarrow \mathcal {U}} \lim _{m\rightarrow \mathcal {U}} \left( \sum _{(I,J)\in \mathcal {D}_m\times \mathcal {D}_n} |I| |J| d_{I,J}\right) ,\\ \mu (D)&= \lim _{m\rightarrow \mathcal {U}} \lim _{n\rightarrow \mathcal {U}} \left( \sum _{(I,J)\in \mathcal {D}_m\times \mathcal {D}_n} |I| |J| d_{I,J}\right) . \end{aligned}$$

For $\delta >0$, there exist infinite $M,N\subset \mathbb {N}$ (not necessarily in $\mathcal {U}$) such that

1. for every $$(n,m)\in [N,M]$$, $$\Big |\sum _{(I,J)\in \mathcal {D}_m\times \mathcal {D}_n} |I| |J| d_{I,J} - \lambda (D)\Big | < 1/n$$,
2. and for every $(m,n)\in [M,N]$, $\Big |\sum _{(I,J)\in \mathcal {D}_m\times \mathcal {D}_n} |I| |J| d_{I,J} - \mu (D)\Big | < 1/m$.

We first apply Proposition 3.26, to find faithful Haar systems $\widetilde{H}^{(1)}$ and $\widetilde{K}^{(1)}$ relative to frequencies $(m_i^{(1)})_{i=0}^\infty \subset M$ and $(n_j^{(1)})_{i=0}^\infty \subset N$, such that $D|_{\widetilde{H}^{(1)} \otimes \widetilde{K}^{(1)}}$ satisfies the upper and lower triangular conditions for appropriate $\eta$. We next apply, in that order Proposition 3.30, 3.31, and 3.32 to find $\tilde{H} = \widetilde{H}^{(4)} *\widetilde{H}^{(3)} *\widetilde{H}^{(2)}*\widetilde{H}^{(1)}$, $\tilde{K} = \widetilde{K}^{(4)} *\widetilde{K}^{(3)} *\widetilde{K}^{(2)}*\widetilde{K}^{(1)}$, such that $D_{\tilde{H}\otimes \tilde{K}}$ is $(\eta ,\delta )$-stabilized.

Regardless of the frequencies associated to $\widetilde{H}^{(i)}$, $\widetilde{K}^{(i)}$, $i=2,3,4$, by Proposition 3.3, $\tilde{H}$ is a faithful Haar system relative frequencies $(m_i)_{i=0}^\infty \subset M$, and $\tilde{K}$ is a faithful Haar system relative to frequencies $(n_j)_{j=0}^\infty \subset N$. But then, by Proposition 3.14

$$\begin{aligned} \lambda _\mathcal {U}(\tilde{D})&= \lim _{j\rightarrow \infty } \lim _{i\rightarrow \infty } \sum _{I\in \mathcal {D}_i}\sum _{J\in \mathcal {D}_j}|I||J|\tilde{d}_{I,J} = \lim _{j\rightarrow \infty } \lim _{i\rightarrow \infty } \sum _{I\in \mathcal {D}_{m_i}}\sum _{J\in \mathcal {D}_{n_j}}|I||J| d_{I,J} = \lambda _\mathcal {U}(D)\text { and}\\ \mu _\mathcal {U}(\tilde{D})&= \lim _{i\rightarrow \infty } \lim _{j\rightarrow \infty } \sum _{I\in \mathcal {D}_i}\sum _{J\in \mathcal {D}_j}|I||J|\tilde{d}_{I,J} = \lim _{i\rightarrow \infty } \lim _{j\rightarrow \infty } \sum _{I\in \mathcal {D}_{m_i}}\sum _{J\in \mathcal {D}_{n_j}}|I||J| d_{I,J} = \mu _\mathcal {U}(D). \end{aligned}$$

$$\square $$

## Haar system Hardy spaces in two parameters

We prove the basic important properties of Haar system Hardy spaces. In particular, we prove that they satisfy Definition 2.30 a for $C=1$, as well as the well boundedness of certain coordinate projections. Before we do so, we prove and collect some elementary facts about Haar system Hardy spaces.

Let $Z = Z(\varvec{\sigma },X,Y)\in \mathcal{H}\mathcal{H}(\delta ^2)$, and for each $I,J\in \mathcal {D}$, let the linear functional $\ell _{I,J}:Z\rightarrow \mathbb {R}$ be given by

$$\begin{aligned} \ell _{I,J}(z) = \int \int h_I(s)k_J(t) z(s,t)\textrm{d} t\textrm{d} s. \end{aligned}$$

We define the space $Z'$ as the closure of $\langle \{ \ell _{I,J}: I,J\in \mathcal {D} \} \rangle$ in $Z^*$ and equip it with the inherited norm, i.e.,

4.1 $$\begin{aligned} \left\| \sum _{I,J\in \mathcal {D}} a_{I,J}\ell _{I,J}\right\| _{Z'} = \sup _{\Vert z\Vert _Z\le 1} \left| \int \int \sum _{I,J\in \mathcal {D}} a_{I,J} h_I(s)k_J(t) z(s,t)\textrm{d} t\textrm{d} s\right| . \end{aligned}$$

Now we define the scalar product $\langle \cdot ,\cdot \rangle :Z'\times Z\rightarrow \mathbb {R}$ by first defining it as the linear extension of

4.2 $$\begin{aligned} \langle \ell _{I,J}, z\rangle = \int \int h_I(s)k_J(t) z(s,t)\textrm{d} t\textrm{d} s, \qquad I,J\in \mathcal {D},\text { and } z\in Z, \end{aligned}$$

and then uniquely extending it to all of $Z'$. We will as usual identify each $\ell _{I,J}$ with $h_I\otimes k_J$ and we will write $\langle h_I\otimes k_J, z\rangle$ instead of $\langle \ell _{I,J}, z\rangle$.

In the following Proposition 4.1, we calculate the norm of $\ell _{I,J}$.

### Proposition 4.1

Let $I,J\in \mathcal {D}$ and recall that $\ell _{I,J}$ is defined in (4.2). Then we have that

4.3 $$\begin{aligned} \sup _{\Vert z\Vert _Z\le 1} |\langle \ell _{I,J}, z\rangle | = \Vert \ell _{I,J}\Vert _{Z^*} = \frac{|I||J|}{\Vert h_I\Vert _X\Vert k_J\Vert _Y}. \end{aligned}$$

### Notation 4.2

We define the bijective function $\iota :\mathcal {D}\rightarrow \mathbb {N}$ by

$$\begin{aligned} \left[ \frac{i-1}{2^j},\frac{i}{2^j}\right) \overset{\iota }{\mapsto }\ 2^j + i-1. \end{aligned}$$

The function $\iota$ defines a linear order on $\mathcal {D}$ that maps each $\mathcal {D}_n$ to $\{2^n,\ldots ,2^{n+1}-1\}$. We will sometimes write $I\le J$ if $\iota (I)\le \iota (J)$, $I,J\in {\mathcal {D}}$.

Henceforth, whenever we write $\sum _{I\in \mathcal {D}}$ we will always mean that the sum is taken with this linear order $\iota$.

### Proof

The lower estimate is easily established by considering $\langle \ell _{I,J},h_I\otimes k_J\rangle$. For the upper estimate, let $z = \sum _{K,L\in \mathcal {D}} a_{K,L} h_K\otimes k_L\in \langle \{h_I\otimes k_J: I,J\in \mathcal {D}\} \rangle$ and define

4.4 $$\begin{aligned} b_K(t) = \sum _{L\in \mathcal {D}} \sigma _K^{(1)}\sigma _L^{(2)}a_{K,L} k_L(t), \qquad t\in [0,1),\ K\in \mathcal {D}. \end{aligned}$$

As in the proof of Proposition 4.5 (see (4.7) and (4.8)), the functions

$$\begin{aligned} s&\mapsto \left\| t\mapsto {{\,\mathrm{\mathbb {E}}\,}}\left| b_I(t) h_I(s) + \sum _{K< I} b_K(t) h_K(s) \right| \right\| _Y,\\ s&\mapsto \left\| t\mapsto {{\,\mathrm{\mathbb {E}}\,}}\left| b_I(t) h_I(s) - \sum _{K < I} b_K(t) h_K(s) \right| \right\| _Y \end{aligned}$$

are equimeasurable, and therefore they have the same norm in *X*. Thus, the convexity of the norm in *Z* yields

$$\begin{aligned}{} & {} \left\| h_I\otimes b_I \right\| _Z \le \frac{1}{2} \left\| h_I\otimes b_I + \sum _{K< I} h_K\otimes b_K \right\| _Z\\{} & {} + \frac{1}{2} \left\| h_I\otimes b_I - \sum _{K < I} h_K\otimes b_K \right\| _Z = \left\| \sum _{K\le I} h_K\otimes b_K\right\| _Z. \end{aligned}$$

By Proposition 4.5 and recalling (4.4), we obtain

4.5 $$\begin{aligned}{} & {} \left\| h_I\otimes b_I \right\| _Z \le \left\| \sum _{K\le I} h_K\otimes b_K\right\| _Z \le \left\| \sum _{K\in \mathcal {D}} h_K\otimes b_K\right\| _Z\nonumber \\{} & {} = \left\| \sum _{K,L\in \mathcal {D}} a_{K,L} h_K\otimes k_L\right\| _Z = \Vert z\Vert _Z. \end{aligned}$$

Using Proposition 4.5 again gives us

4.6 $$\begin{aligned} \Vert h_I\otimes b_I\Vert _Z = \Vert h_I\Vert _X \left\| {{\,\mathrm{\mathbb {E}}\,}}\left| \sum _{L\in \mathcal {D}} \sigma _L^{(2)} a_{I,L} k_L \right| \right\| _Y \ge \Vert h_I\Vert _X \left\| \int _0^1 \left| \sum _{L\le J} \sigma _L^{(2)} a_{I,L} k_L \right| \right\| _Y. \end{aligned}$$

Since the functions

$$\begin{aligned} {{\,\mathrm{\mathbb {E}}\,}}\left| \sigma _J^{(2)} a_{I,J} k_J + \sum _{L< J} \sigma _L^{(2)} a_{I,L} k_L \right| \qquad \text {and}\qquad {{\,\mathrm{\mathbb {E}}\,}}\left| \sigma _J^{(2)} a_{I,J} k_J - \sum _{L < J} \sigma _L^{(2)} a_{I,L} k_L \right| \end{aligned}$$

are equimeasurable, thus, they have the same norm in *X*. Using the convexity of the norm in *Y* yields

$$\begin{aligned} |a_{I,J}| \Vert k_J\Vert _Y&= \Vert a_{I,J} k_J\Vert _Y \le \frac{1}{2} \left\| \int _0^1 \left| \sigma _J^{(2)} a_{I,J} k_J + \sum _{L< J} \sigma _L^{(2)}(v) a_{I,L} k_L \right| \right\| _Y\\&\quad + \frac{1}{2} \left\| {{\,\mathrm{\mathbb {E}}\,}}\left| \sigma _J^{(2)} a_{I,J} k_J - \sum _{L < J} \sigma _L^{(2)} a_{I,L} k_L \right| \right\| _Y\\&= \left\| {{\,\mathrm{\mathbb {E}}\,}}\left| \sum _{L\le J} \sigma _L^{(2)} a_{I,L} k_L \right| \right\| _Y. \end{aligned}$$

Combining the latter estimate with (4.6) and (4.5) yields

$$\begin{aligned} |a_{I,J}| \Vert h_I\Vert _X \Vert k_J\Vert _Y \le \Vert z\Vert _Z, \end{aligned}$$

and noting that $|\langle \ell _{I,J}, z\rangle | = |I| |J| |a_{I,J}|$ establishes (4.3).$\square$

### Remark 4.3

Let $Z = Z(\varvec{\sigma },X,Y)\in \mathcal{H}\mathcal{H}(\delta ^2)$. By Proposition 4.1, identifying $h_I\otimes k_J$ with $\ell _{I,J}$ in $Z'$, we can represent any $z'\in Z'$ as

$$\begin{aligned} z' = \sum _{I,J\in \mathcal {D}} a_{I,J} h_I\otimes k_J, \end{aligned}$$

where the convergence of the series is understood in the norm $\Vert \cdot \Vert _{Z'}$ given by

$$\begin{aligned} \Vert z'\Vert _{Z'} = \sup _{\Vert z\Vert _Z\le 1} \left| \int \int z'(s,t) z(s,t)\textrm{d} t\textrm{d} s\right| . \end{aligned}$$

Altogether, $\langle Z', Z, \langle \cdot ,\cdot \rangle \rangle$ is a dual pair of Banach spaces and by the definition of $Z'$ and Proposition 4.5 we have

$$\begin{aligned} |\langle z', z\rangle | \le \Vert z'\Vert _{Z'} \Vert z\Vert _Z, \ z'\in Z,\ z\in Z \qquad \text {and}\qquad \Vert z\Vert _Z = \sup _{\Vert z'\Vert _{Z'}\le 1} \langle z', z\rangle , \ z\in Z. \end{aligned}$$

### Remark 4.4

If we define $X'$ and $Y'$ analogously to $Z'$, then by Proposition 4.1 and the fact that $\Vert h_{[0,1)}\Vert _X = \Vert k_{[0,1)}\Vert _Y = 1$ (see Definition 2.6) we have

$$\begin{aligned} \Vert h_I\otimes k_J\Vert _{Z'}\Vert h_I\otimes k_J\Vert _Z = |I| |J|, \qquad \Vert h_I\Vert _{X'}\Vert h_I\Vert _X = |I|, \qquad \Vert k_J\Vert _{X'}\Vert k_J\Vert _X = |J|, \end{aligned}$$

for all $$I,J\in \mathcal {D}$$.

### Basic operators on Haar system Hardy spaces

Here, we collect estimates for bi-parameter basis projections, the sub-restriction operator and dyadic scaling operators.

#### Proposition 4.5

Let $Z = Z(\mathbf {\sigma },X,Y)\in \mathcal{H}\mathcal{H}(\delta ^2)$. Given $I\in \mathcal {D}$, we define the projection $P_{\le I}, P_{\ge I}:H\rightarrow H$ by

$$\begin{aligned} P_{\le I} \left( \sum _{K\in \mathcal {D}} a_K h_K\right) = \sum _{K\le I} a_K h_K \qquad \text {and}\qquad P_{\ge I} \left( \sum _{K\in \mathcal {D}} a_K h_K\right) = \sum _{K\ge I} a_K h_K. \end{aligned}$$

Then for all $I,J\in \mathcal {D}$, we have the estimates

$$\begin{aligned} \Vert P_{\le I}\otimes P_{\le J}:Z\rightarrow Z\Vert&\le 1,&\Vert P_{\ge I}\otimes P_{\ge J}:Z\rightarrow Z\Vert&\le 4,\\ \Vert P_{\le I}\otimes P_{\ge J}:Z\rightarrow Z\Vert&\le 2,&\Vert P_{\ge I}\otimes P_{\le J}:Z\rightarrow Z\Vert&\le 2. \end{aligned}$$

#### Remark 4.6

Given $$I\in \mathcal {D}$$, we introduce the following additional notation: We define $$P_{< I} = 0$$, if $$I=[0,1)$$, and $$P_{< I} = P_{\le J}$$, if $$\iota (J) = \iota (I) + 1$$ and put $$P_{\ge I} = I - P_{< I}$$, $$P_{> I} = I - P_{\le I}$$ as well as $$p_I = P_{\le I} - P_{< I}$$. Moreover, given $$k\in \mathbb {N}$$, we define $${{\,\mathrm{\mathbb {E}}\,}}_k = P_{\le I}$$, where $$I\in \mathcal {D}$$ is the largest (with respect to the standard linear order) dyadic interval with $$|I|\ge 2^{-k+1}$$.

Thus, by Proposition 4.5, the projections $P_{\ge I}$, $P_{> I}$, $p_I$ are all bounded by 2 and ${{\,\mathrm{\mathbb {E}}\,}}_k$ is bounded by 1 on any one-parameter Haar system Hardy space. Additionally, we note that tensoring any two of these operators yields a projection on *Z* bounded by either 1, 2 or 4.

#### Proof of Proposition 4.5

It suffices to estimate on a vector $z = \sum _{K,L\in \mathcal {D}} a_{K,L} h_K\otimes k_L\in \mathcal {V}(\delta ^2)$, with $a_{K,L}\in \mathbb {R}$. Observe that

4.7 $$\begin{aligned} \begin{aligned} \Vert P_{\le I}\otimes P_{\le J}z\Vert _{Z}&= \left\| s\mapsto \left\| t\mapsto {{\,\mathrm{\mathbb {E}}\,}}\left| \sum _{K\le I}\sum _{L\le J} \sigma _K^{(1)}\sigma _L^{(2)} a_{K,L} h_K(s) k_L(t) \right| \right\| _Y \right\| _X\\&= \left\| s\mapsto \left\| t\mapsto {{\,\mathrm{\mathbb {E}}\,}}\left| \sum _{K\le I} h_K(s) b_K(t) \right| \right\| _Y \right\| _X =\left\| \sum _{K\le I}h_K\otimes b_K\right\| _{Z}, \end{aligned} \end{aligned}$$

where we defined

4.8 $$\begin{aligned} b_K(t) = \sum _{L\le J} \sigma _K^{(1)}\sigma _L^{(2)} a_{K,L} k_L(t), \qquad t\in [0,1),\ K\in \mathcal {D}. \end{aligned}$$

Note that the functions

$$\begin{aligned} s&\mapsto \left\| t\mapsto {{\,\mathrm{\mathbb {E}}\,}}\left| \sum _{K< I} h_K(s)b_K(t) + h_I(s)b_I(t) \right| \right\| _Y,\\ s&\mapsto \left\| t\mapsto {{\,\mathrm{\mathbb {E}}\,}}\left| \sum _{K < I} h_K(s)b_K(t) - h_I(s)b_I(t) \right| \right\| _Y \end{aligned}$$

are equimeasurable, and therefore they have the same norm in *X*. Thus, by convexity of the norm in *Z*, (4.7) and (4.8), we obtain

$$\begin{aligned} \begin{aligned} \left\| \sum _{K< I} h_K\otimes b_K \right\| _{Z}&\le \frac{1}{2} \left\| \sum _{K< I} h_K\otimes b_K + h_K\otimes b_I\right\| _{Z} + \frac{1}{2} \left\| \sum _{K < I}h_K\otimes b_K - h_K\otimes b_I \right\| _{Z}\\&= \left\| \sum _{K\le I}h_K\otimes b_K\right\| _{Z}. \end{aligned} \end{aligned}$$

Iterating the latter inequality yields

4.9 $$\begin{aligned} \begin{aligned} \Vert P_{\le I}\otimes P_{\le J}z\Vert _{Z}&\le \left\| \sum _{K\in \mathcal {D}^{1}}h_K\otimes b_K \right\| _{Z^\Omega } = \left\| \sum _{K\in \mathcal {D}^{1}} \sum _{L\le J} a_{K,L} h_K\otimes k_L \right\| _{Z} = \Vert I_X\otimes P_{\le J} z\Vert _{Z}. \end{aligned} \end{aligned}$$

Similarly, we note that

4.10 $$\begin{aligned} \begin{aligned} \Vert I_X\otimes P_{\le J} w\Vert _{Z}&= \left\| s\mapsto \int _0^1 \left\| t\mapsto {{\,\mathrm{\mathbb {E}}\,}}\left| \sum _{K\in \mathcal {D}} \sum _{L\le J} \sigma _K^{(1)}\sigma _L^{(2)}a_{K,L} h_K(s) k_L(t) \right| \right\| _Y \right\| _X\\&= \left\| s\mapsto \left\| t\mapsto {{\,\mathrm{\mathbb {E}}\,}}\left| \sum _{L\le J} c_L(s) k_L(t) \right| \right\| _Y \right\| _X = \left\| \sum _{L\le J} k_L \otimes c_L \right\| _Z, \end{aligned} \end{aligned}$$

where we put

4.11 $$\begin{aligned} c_L(s) = \sum _{K\in \mathcal {D}} h_K(s) \sigma _K^{(1)}\sigma _L^{(2)}a_{K,L}, \qquad s\in [0,1),\ L\in \mathcal {D}. \end{aligned}$$

Then for fixed $s\in [0,1)$, the functions

$$\begin{aligned} t\mapsto {{\,\mathrm{\mathbb {E}}\,}}\left| \sum _{L< J}k_L(t) c_L(s) +k_J(t) c_J(s) \right| ,\quad t\mapsto {{\,\mathrm{\mathbb {E}}\,}}\left| \sum _{L < J} k_L(t) c_L(s) - k_J(t)c_J(s) \right| , \end{aligned}$$

are equimeasurable and therefore have the same norm in *Y*. Again, using the convexity of the norm in *Z* yields

$$\begin{aligned}{} & {} \left\| \sum _{L< J} c_L k_L\right\| _Z \le \frac{1}{2} \left\| \sum _{L< J} k_L\otimes c_L + k_J\otimes c_J\right\| _Z\\{} & {} + \frac{1}{2} \left\| \sum _{L < J} k_L\otimes c_L - k_J\otimes c_J\right\| _Z \le \left\| \sum _{L\le J} k_L\otimes c_L\right\| _Z. \end{aligned}$$

Recalling (4.9), (4.10) and iterating the above inequality, we obtain

$$\begin{aligned} \Vert P_{\le I}\otimes P_{\le J}z\Vert _Z \le \left\| \sum _{L\in \mathcal {D}} k_L\otimes c_L\right\| _Z = \left\| \sum _{K,L\in \mathcal {D}} a_{K,L}h_K\otimes k_L \right\| _Z = \Vert z\Vert _Z. \end{aligned}$$

The estimate for $I_X\otimes P_{\le J}$ follows by choosing $I\in \mathcal {D}$ so large that $I_X\otimes P_{\le J} z = P_{\le I}\otimes P_{\le J} z$, and the estimate for $P_{\le I}\otimes I_Y$ follows by choosing $J\in \mathcal {D}$ so large that $P_{\le I}\otimes I_Yz = P_{\le I}\otimes P_{\le J}z$. The other estimates follow immediately from the above estimates. $\square$

#### Proposition 4.7

Let $Z = Z(\mathbf {\sigma },X,Y)\in \mathcal{H}\mathcal{H}(\delta ^2)$ and let $K_0,L_0\in \mathcal {D}$. Define the sub-restriction operator $S_{K_0, L_0}:Z\rightarrow Z$ by

$$\begin{aligned} S_{K_0, L_0} \left( \sum _{K,L\in \mathcal {D}} a_{K,L} h_K\otimes k_L \right) = \sum _{\begin{array}{c} K\subset K_0\\ L\subset L_0 \end{array}} a_{K,L} h_K\otimes k_L. \end{aligned}$$

Then $S_{K_0, L_0}$ is a Haar multiplier with $\Vert S_{K_0, L_0}:Z\rightarrow Z\Vert \le 4$.

#### Proof

Consider $z = \sum _{K,L\in \mathcal {D}} a_{K,L} h_K\otimes k_L\in \mathcal {V}(\delta ^2)$ with $a_{K,L}\in \mathbb {R}$. Note that

$$\begin{aligned} \Vert S_{K_0, L_0} z\Vert _{Z} = \left\| s\mapsto \left\| t\mapsto {{\,\mathrm{\mathbb {E}}\,}}\left| \sum _{\begin{array}{c} K\subset K_0\\ L\subset L_0 \end{array}} \sigma _K^{(1)}\sigma _L^{(2)}a_{K,L} h_K(s) k_L(t) \right| \right\| _Y \right\| _X. \end{aligned}$$

Suppose that $|K_0| = 2^{-k_0}$ and $|L_0| = 2^{-l_0}$, then for fixed $s,t\in [0,1)$, we have

$$\begin{aligned} \left| \sum _{\begin{array}{c} K\subset K_0\\ L\subset L_0 \end{array}} \sigma _K^{(1)}\sigma _L^{(2)}a_{K,L} h_K(s) k_L(t) \right| \le \left| \sum _{\begin{array}{c} |K|\le 2^{-k_0}\\ |L|\le 2^{-l_0} \end{array}} \sigma _K^{(1)}\sigma _L^{(2)}a_{K,L} h_K(s) k_L(t) \right| . \end{aligned}$$

Taking the expectation ${{\,\mathrm{\mathbb {E}}\,}}$ on both sides of the above inequality and using Proposition 2.7 (v) twice yields

$$\begin{aligned} \Vert S_{K_0, L_0}z\Vert _{Z}&\le \left\| s\mapsto \left\| t\mapsto {{\,\mathrm{\mathbb {E}}\,}}\left| \sum _{\begin{array}{c} |K|\le 2^{-k_0}\\ |L|\le 2^{-l_0} \end{array}} \sigma _K^{(1)}\sigma _L^{(2)}a_{K,L} h_K(s) k_L(t) \right| \right\| _Y \right\| _X\\&= \left\| \sum _{\begin{array}{c} |K|\le 2^{-k_0}\\ |L|\le 2^{-l_0} \end{array}} a_{K,L}\otimes h_K\otimes k_L \right\| _{Z} = \left\| \left( (I_X - {{\,\mathrm{\mathbb {E}}\,}}_{k_0})\otimes (I_Y - {{\,\mathrm{\mathbb {E}}\,}}_{l_0})\right) z \right\| _{Z}. \end{aligned}$$

Applying Proposition 4.5 (see Remark 4.6 for the definition of ${{\,\mathrm{\mathbb {E}}\,}}_{k_0}$, ${{\,\mathrm{\mathbb {E}}\,}}_{l_0}$) proves the estimate for $S_{K_0, L_0}$.$\square$

#### Proposition 4.8

Let $Z = Z(\mathbf {\sigma },X,Y)\in \mathcal{H}\mathcal{H}(\delta ^2)$, $I_0,J_0\in \mathcal {D}$ with $|I_0| = |J_0| = 2^{-l_0}$, and let $\rho _0:[0,1)\rightarrow I_0$ and $\tau _0:[0,1)\rightarrow J_0$ denote the unique increasing, affine linear and bijective functions. Then the operators $\Delta _{I_0, J_0}, \Upsilon _{I_0, J_0}:Z\rightarrow Z$ defined by

$$\begin{aligned} \Delta _{I_0, J_0} \left( \sum _{I,J\in \mathcal {D}} a_{I,J} h_I\otimes k_J\right)&= \sum _{I,J\in \mathcal {D}} a_{I,J} h_{\rho _0(I)}\otimes k_{\tau _0(J)},\\ \Upsilon _{I_0, J_0} \left( \sum _{I,J\in \mathcal {D}} a_{I,J} h_I\otimes k_J\right)&= \sum _{\begin{array}{c} I\subset I_0\\ J\subset J_0 \end{array}} a_{I,J} h_{\rho _0^{-1}(I)}\otimes k_{\tau _0^{-1}(J)}, \end{aligned}$$

satisfy the estimates

$$\begin{aligned} \Vert \Delta _{I_0, J_0} z\Vert _Z \le \Vert z\Vert _Z \quad \text {and}\quad \Vert \Upsilon _{I_0, J_0} z\Vert _Z \le 4^{l_0} \Vert S_{I_0, J_0} z\Vert _Z \le 4^{l_0+1} \Vert z\Vert _Z, \qquad z\in Z. \end{aligned}$$

Moreover, we have that $\Upsilon _{I_0, J_0} \Delta _{I_0, J_0} = I_Z$ and $S_{I_0, J_0}\Delta _{I_0, J_0} = \Delta _{I_0, J_0}$.

#### Proof

Let $$N\in \mathbb {N}$$ and $$z = \sum _{\begin{array}{c} I,J\in \mathcal {D}\\ |I|,|J|\ge 2^{-N+1} \end{array}} a_{I,J} h_I\otimes k_J$$.$$\square $$

#### Estimate for $\Delta _{I_0, J_0}$

 For $s,t s,t\in [0,1)$ we define

$$\begin{aligned} f_1(s,t)&= {{\,\mathrm{\mathbb {E}}\,}}\left| \sum _{I,J\in \mathcal {D}} \sigma _I^{(1)}\sigma _J^{(2)}a_{I,J} h_I(s) k_J(t) \right| , \text { and } \\ f_2(s,t)&= {{\,\mathrm{\mathbb {E}}\,}}\left| \sum _{I,J\in \mathcal {D}} \sigma _{\rho _0(I)}^{(1)}\sigma _{\tau _0(J)}^{(2)}a_{I,J} h_I(s) k_J(t) \right| , \end{aligned}$$

and observe that

$$\begin{aligned} \Vert z\Vert _Z = \left\| s\mapsto \left\| t\mapsto f_1(s,t) \right\| _Y \right\| _X = \left\| s\mapsto \left\| t\mapsto f_2(s,t) \right\| _Y \right\| _X. \end{aligned}$$

Now, we pick a measurable set *A* such that $|A\cap I| = 2^{-l_0} |I|$ for all $I\in \mathcal {D}$ with $|I|\ge 2^{-N}$ and observe that by Proposition 2.7 (v).

$$\begin{aligned} \Vert z\Vert _Z \ge \left\| s\mapsto \left\| t\mapsto \chi _A(t)f_2(s,t) \right\| _Y \right\| _X. \end{aligned}$$

Next, we define the function

$$\begin{aligned} f_3(s,t) = {{\,\mathrm{\mathbb {E}}\,}}\left| \sum _{I,J\in \mathcal {D}} \sigma _{\rho _0(I)}^{(1)}\sigma _{\tau _0(J)}^{(2)}a_{I,J} h_{I}(s) k_{\tau _0(J)}(t) \right| , \qquad s,t\in [0,1) \end{aligned}$$

and note that for fixed *s* the functions $t\mapsto \chi _A(t) f_2(s,t)$ and $t\mapsto f_3(s,t)$ are equimeasurable. Thus, by Proposition 2.7 (v)

$$\begin{aligned} \Vert z\Vert _Z \ge \left\| s\mapsto \left\| t\mapsto f_3(s,t) \right\| _Y \right\| _X \ge \left\| s\mapsto \int _0^1 \left\| t\mapsto \chi _A(s) f_3(s,t) \right\| _Y \right\| _X. \end{aligned}$$

Next, we define for $s\in [0,1)$

$$\begin{aligned} f_4(s)&= \left\| t\mapsto \chi _A(s) f_3(s,t) \right\| _Y, \text { and } \\ f_5(s)&= \left\| t\mapsto {{\,\mathrm{\mathbb {E}}\,}}\left| \sum _{I,J\in \mathcal {D}} \sigma _{\rho _0(I)}^{(1)}\sigma _{\tau _0(J)}^{(2)}a_{I,J} h_{\rho _0(I)}(s) k_{\tau _0(J)}(t) \right| \right\| _Y, \end{aligned}$$

and observe that $f_4$ and $f_5$ are equimeasurable, which yields

$$\begin{aligned} \Vert z\Vert _Z \ge \Vert f_5\Vert _X. \end{aligned}$$

Finally, we note that $\Vert f_5\Vert _X = \Vert \Delta _{I_0, J_0} z\Vert _Z$ and conclude $\Vert \Delta _{I_0, J_0} z\Vert _Z\le \Vert z\Vert _Z$.

#### Estimate for $\Upsilon _{I_0, J_0}$

 First, we pick a measurable partition $A_k$, $1\le k \le 2^{l_0}$, of [0, 1) such that $|A_k\cap I| = 2^{-l_0} |I|$ for all $I\in \mathcal {D}$ with $|I|\ge 2^{-l_0}$ and $1\le k \le 2^{l_0}$. Secondly, we define the functions

$$\begin{aligned} g_1(s,t)&= {{\,\mathrm{\mathbb {E}}\,}}\left| \sum _{\begin{array}{c} I\subset I_0\\ J\subset J_0 \end{array}} \sigma _{\rho _0^{-1}(I)}^{(1)}\sigma _{\tau _0^{-1}(J)}^{(2)} a_{I,J} h_{\rho _0^{-1}(I)}(s) k_{\tau _0^{-1}(J)}(t) \right| ,{} & {} s,t\in [0,1)\\ g_2(s,t)&= {{\,\mathrm{\mathbb {E}}\,}}\left| \sum _{\begin{array}{c} I\subset I_0\\ J\subset J_0 \end{array}} \sigma _{I}^{(1)}\sigma _{J}^{(2)} a_{I,J} h_{\rho _0^{-1}(I)}(s) k_{\tau _0^{-1}(J)}(t) \right| ,{} & {} s,t\in [0,1) \end{aligned}$$

and note that

$$\begin{aligned} \Vert \Upsilon _{I_0, J_0}z\Vert _Z = \left\| s\mapsto \left\| t\mapsto g_1(s,t) \right\| _Y \right\| _X = \left\| s\mapsto \left\| t\mapsto g_2(s,t) \right\| _Y \right\| _X. \end{aligned}$$

Since the $A_k$, $1\le k\le 2^{l_0}$ are a measurable partition of the unit interval, we have

$$\begin{aligned} \Vert \Upsilon _{I_0, J_0}z\Vert _Z&= \left\| s\mapsto \left\| t\mapsto \sum _{k=1}^{2^{l_0}} \chi _{A_k}(t) g_2(s,t) \right\| _Y \right\| _X\\&\le \sum _{k=1}^{2^{l_0}} \left\| s\mapsto \left\| t\mapsto \chi _{A_k}(t) g_2(s,t) \right\| _Y \right\| _X. \end{aligned}$$

Next, we put

$$\begin{aligned} g_3(s,t) = {{\,\mathrm{\mathbb {E}}\,}}\left| \sum _{\begin{array}{c} I\subset I_0\\ J\subset J_0 \end{array}} \sigma _I^{(1)}\sigma _J^{(2)}a_{I,J} h_{\rho _0^{-1}(I)}(s) k_{J}(t) \right| \qquad s,t\in [0,1), \end{aligned}$$

and observe that for fixed $s\in [0,1)$ and $1\le k\le 2^{l_0}$, the functions $t\mapsto \chi _{A_k}(t) g_2(s,t)$ and $t\mapsto g_3(s,t)$ are equimeasurable. Hence, by Proposition 2.7 (v) we obtain

$$\begin{aligned} \Vert \Upsilon _{I_0, J_0}z\Vert _Z \le 2^{l_0} \left\| s\mapsto \left\| t\mapsto g_3(s,t) \right\| _Y \right\| _X. \end{aligned}$$

Again, since the $A_k$, $1\le k\le 2^{l_0}$ are a measurable partition of the unit interval

$$\begin{aligned} \Vert \Upsilon _{I_0, J_0}z\Vert _Z&\le 2^{l_0} \left\| s\mapsto \left\| t\mapsto \sum _{k=1}^{2^{l_0}} \chi _{A_k}(s) g_3(s,t) \right\| _Y \right\| _X\\&\le 2^{l_0} \sum _{k=1}^{2^{l_0}} \left\| s\mapsto \left\| t\mapsto \chi _{A_k}(s) g_3(s,t) \right\| _Y \right\| _X. \end{aligned}$$

Now, we define the functions

$$\begin{aligned} g_4^{(k)}(s)&= \left\| t\mapsto \chi _{A_k}(s) g_3(s,t) \right\| _Y,{} & {} s\in [0,1),\ 1\le k\le 2^{l_0},\\ g_5(s)&= \Bigl \Vert t\mapsto {{\,\mathrm{\mathbb {E}}\,}}\Bigl | \sum _{\begin{array}{c} I\subset I_0\\ J\subset J_0 \end{array}} \sigma _{I}^{(1)}\sigma _{J}^{(2)}a_{I,J} h_{I}(s) k_{J}(t) \Bigr | \textrm{d} v \textrm{d} u_2 \Bigr \Vert _Y,{} & {} s\in [0,1), \end{aligned}$$

and observe that $g_4^{(k)}$ and $g_5$ are equimeasurable for all $1\le k\le 2^{l_0}$. Again, using Proposition 2.7 (v) yields

$$\begin{aligned} \Vert \Upsilon _{I_0, J_0}z\Vert _Z \le 4^{l_0} \Vert g_5\Vert _X. \end{aligned}$$

Finally, we note that $\Vert g_5\Vert _X = \Vert S_{I_0, J_0} z\Vert _Z$ and appeal to Proposition 4.7 to conclude $\Vert \Upsilon _{I_0, J_0}z\Vert _Z\le 4^{l_0} \Vert S_{I_0, J_0} z\Vert _Z\le 4^{l_0+1} \Vert z\Vert _Z$.

The additional claims that $\Upsilon _{I_0, J_0} \Delta _{I_0, J_0} = I_Z$ and $S_{I_0, J_0}\Delta _{I_0, J_0} = \Delta _{I_0, J_0}$ are obvious. $\square$

### Block bases and projections on Haar system Hardy spaces

Here, we prove that Haar system Hardy spaces satisfy Definition 2.30 a for $C = 1$.

#### Theorem 4.9

Let $Z = Z(\mathbf {\sigma },X,Y)\in \mathcal{H}\mathcal{H}(\delta ^2)$ and let $(\widetilde{h}_I: I\in \mathcal {D})$ and $(\widehat{k}_J: J\in \mathcal {D})$ be faithful Haar systems in *X* and *Y*, respectively. Let $B,A:Z\rightarrow Z$ be given by

4.12 $$\begin{aligned} Bz = \sum _{I,J\in \mathcal {D}} \frac{\langle h_I\otimes k_J, z \rangle }{|I| |J|} \widetilde{h}_I\otimes \widehat{k}_J \qquad \text {and}\qquad Az = \sum _{I,J\in \mathcal {D}} \frac{\langle \widetilde{h}_I\otimes \widehat{k}_J, z \rangle }{|I| |J|} h_I\otimes k_J. \end{aligned}$$

Then the operators *A*, *B* satisfy

$$\begin{aligned} I_Z = AB \qquad \text {and}\qquad \Vert B\Vert , \Vert A\Vert \le 1. \end{aligned}$$

In order to prove Theorem 4.9, we need the following lemma (we refer to [40, Lemma 4.3] for a proof).

#### Lemma 4.10

Let $X\in \mathcal {H}(\delta )$ and let $\mathcal {F}$ denote a $\sigma$-algebra generated by the finite unions of dyadic intervals $A_i$, $1\le i\le m$. By ${{\,\mathrm{\mathbb {E}}\,}}^{\mathcal {F}}$ we denote the conditional expectation with respect to $\mathcal {F}$. Then

$$\begin{aligned} \Vert {{\,\mathrm{\mathbb {E}}\,}}^{\mathcal {F}} x\Vert _X \le \Vert x\Vert _X, \qquad x\in \langle \{h_I : I\in \mathcal {D}\} \rangle . \end{aligned}$$

#### Proof of Theorem 4.9

Let $z = \sum _{K,L\in \mathcal {D}} a_{K,L} h_K\otimes k_L\in \langle \{h_K\otimes k_L: K,L\in \mathcal {D}\} \rangle$ and suppose that

4.13 $$\begin{aligned} \widetilde{h}_I = \sum _{K\in \mathcal {A}_I} \theta _K h_K \qquad \text {and}\qquad \widehat{k}_J = \sum _{L\in \mathcal {B}_J} \varepsilon _L k_L \end{aligned}$$

for suitable collections of dyadic intervals $\mathcal {A}_I$, $\mathcal {B}_J$, $I,J\in \mathcal {D}$. For any $I,J\in \mathcal {D}$, we put $A_I = \bigcup \mathcal {A}_I$ and $B_J = \bigcup \mathcal {B}_J$. Pick $N\in \mathbb {N}$ such that $a_{K,L} = 0$, whenever there exists $I_0\in \mathcal {D}_{N-1}$ and $K_0\in \mathcal {A}_{I_0}$ such that $K\subset K_0$ or there exists $J_0\in \mathcal {D}_{N-1}$ and $L_0\in \mathcal {B}_{J_0}$ such that $L\subset L_0$. We define the collections $\mathcal {A} = \bigcup _{I\in \mathcal {D}}\mathcal {A}_I$ and $\mathcal {B} = \bigcup _{J\in \mathcal {D}}\mathcal {B}_J$. By the definition of the norm in *Z*

4.14 $$\begin{aligned} \Vert z\Vert _Z = \left\| s\mapsto \left\| t\mapsto {{\,\mathrm{\mathbb {E}}\,}}\left| \sum _{K,L\in \mathcal {D}} \sigma _K^{(1)} \sigma _L^{(2)} a_{K,L} h_K(s) k_L(t) \right| \right\| _Y \right\| _X. \end{aligned}$$

#### Estimates for B

By the definitions of the operator *B* and the norm in *Z* and by (4.13), we obtain that is equal to

$$\begin{aligned} \Vert B z\Vert _Z = \left\| s\mapsto \left\| t\mapsto {{\,\mathrm{\mathbb {E}}\,}}\left| \sum _{I,J\in \mathcal {D}} a_{I,J} \sum _{\begin{array}{c} K\in \mathcal {A}_I\\ L\in \mathcal {B}_J \end{array}} \sigma _K^{(1)} \sigma _L^{(2)} \theta _K \varepsilon _L h_K(s) k_L(t) \right| \right\| _Y \right\| _X. \end{aligned}$$

For fixed *s*, *t*, note that for each $J\in \mathcal {D}$ there exists at most one $L\in \mathcal {B}_J$ such that $L\ni t$. Hence for such *L*, replacing the Rademacher $\sigma _L^{(2)}$ by $\sigma _J^{(2)}$ yields

$$\begin{aligned} \Vert B z\Vert _Z = \left\| s\mapsto \left\| t\mapsto {{\,\mathrm{\mathbb {E}}\,}}\left| \sum _{I,J\in \mathcal {D}} \sigma _J^{(2)} a_{I,J} \sum _{\begin{array}{c} K\in \mathcal {A}_I\\ L\in \mathcal {B}_J \end{array}} \sigma _K^{(1)}\theta _K \varepsilon _L h_K(s) k_L(t) \right| \right\| _Y \right\| _X. \end{aligned}$$

Exploiting similarly that for fixed *s*, and $I\in \mathcal {D}$, there exists at most one $K\in \mathcal {A}_I$ with $I\ni s$, we can replace $\sigma _K^{(1)}$ with $\sigma _I^{(1)}$ and obtain

4.15 $$\begin{aligned} \begin{aligned} \Vert B z\Vert _Z&= \left\| s\mapsto \left\| t\mapsto {{\,\mathrm{\mathbb {E}}\,}}\left| \sum _{I,J\in \mathcal {D}} \sigma _I^{(1)} \sigma _J^{(2)} a_{I,J} \sum _{\begin{array}{c} K\in \mathcal {A}_I\\ L\in \mathcal {B}_J \end{array}} \theta _K \varepsilon _L h_K(s) k_L(t) \right| \right\| _Y \right\| _X\\&= \left\| s\mapsto \left\| t\mapsto {{\,\mathrm{\mathbb {E}}\,}}\left| \sum _{I,J\in \mathcal {D}} \sigma _I^{(1)} \sigma _J^{(2)} a_{I,J} \widetilde{h}_I(s) \widehat{k}_J(t) \right| \right\| _Y \right\| _X. \end{aligned} \end{aligned}$$

Recall (4.13) for the latter equality. Note that by equimeasurability in *Y*, we first replace $\widehat{k}_J$ by $k_J$ in (4.15) to obtain

$$\begin{aligned} \Vert B z\Vert _Z = \left\| s\mapsto \left\| t\mapsto {{\,\mathrm{\mathbb {E}}\,}}\left| \sum _{I,J\in \mathcal {D}} \sigma _I^{(1)} \sigma _J^{(2)} a_{I,J} \widetilde{h}_I(s) k_J(t) \right| \right\| _Y \right\| _X, \end{aligned}$$

and then use equimeasurability in *X* to replace $\widetilde{h}_I$ by $h_I$:

$$\begin{aligned} \Vert B z\Vert _Z = \left\| s\mapsto \left\| t\mapsto {{\,\mathrm{\mathbb {E}}\,}}\left| \sum _{I,J\in \mathcal {D}} \sigma _I^{(1)} \sigma _J^{(2)} a_{I,J} h_I(s) k_J(t) \right| \right\| _Y \right\| _X = \Vert z\Vert _Z. \end{aligned}$$

#### Estimates for A

Observe that by the faithfulness of the Haar systems $(\widetilde{h}_I: I\in \mathcal {D})$ and $(\widehat{k}_J: J\in \mathcal {D})$, the collections of sets $\{A_{I_0}: I_0\in \mathcal {D}_N\}$ and $\{B_{J_0}: J_0\in \mathcal {D}_N\}$ are both partitions of [0, 1). Let $\mathcal {F}$ denote the $\sigma$-algebra generated by $\{A_{I_0}: I_0\in \mathcal {D}_N\}$ and let $\mathcal {G}$ denote the $\sigma$-algebra generated by $\{B_{J_0}: J_0\in \mathcal {D}_N\}$.

First, let us fix *s*, *t* and split the random function in (4.14) into two parts:

4.16 $$\begin{aligned} \Vert z\Vert _Z = \left\| s\mapsto \left\| t\mapsto {{\,\mathrm{\mathbb {E}}\,}}\left| f(s,t) + g(s,t) \right| \right\| _Y \right\| _X, \end{aligned}$$

where we put

4.17 $$\begin{aligned} \begin{aligned} f_1(s,t)&= \sum _{I,J\in \mathcal {D}^{N-1}} \sum _{\begin{array}{c} K\in \mathcal {A}_{I}\\ L\in \mathcal {B}_J \end{array}} \sigma _K^{(1)} \sigma _L^{(2)} a_{K,L} h_K(s) k_L(t),\\ g(s,t)&= \sum _{(K,L)\notin \mathcal {A}\times \mathcal {B}} \sigma _K^{(1)} \sigma _L^{(2)} a_{K,L} h_K(s) k_L(t) \end{aligned} \end{aligned}$$

Secondly, define

$$\begin{aligned} f_1'(s,t) = \sum _{I,J\in \mathcal {D}^{N-1}} \sum _{\begin{array}{c} K\in \mathcal {A}_{I}\\ L\in \mathcal {B}_J \end{array}} \sigma _K'^{(1)} \sigma _L'^{(2)} a_{K,L} h_K(s) k_L(t), \end{aligned}$$

where $(\sigma _K'^{(1)})$ is an independent copy of $(\sigma _K^{(1)})$ and $(\sigma _L'^{(2)})$ is an independent copy of $(\sigma _L^{(2)})$. We denote the expectation with respect to these copies by ${{\,\mathrm{\mathbb {E}}\,}}'$. Consequently, $f_1(s,t) + g(s,t)$ is equimeasurable to $f_1'(s,t) + g(s,t)$ (with respect to the product measure of the probability space with its independent copy), and we obtain

$$\begin{aligned} \Vert z\Vert _Z = \left\| s\mapsto \left\| t\mapsto {{\,\mathrm{\mathbb {E}}\,}}'{{\,\mathrm{\mathbb {E}}\,}}\left| f_1'(s,t) + g(s,t) \right| \right\| _Y \right\| _X. \end{aligned}$$

For fixed *s*, *t*, we observe that for each $I,J\in \mathcal {D}$, there exists at most one $K\in \mathcal {A}_I$ with $s\in K$ and at most one $L\in \mathcal {B}_J$ with $t\in L$. Hence, the random function

4.18 $$\begin{aligned} f_2'(s,t) = \sum _{I,J\in \mathcal {D}^{N-1}} \sigma _I^{(1)} \sigma _J^{(2)} \sum _{\begin{array}{c} K\in \mathcal {A}_{I}\\ L\in \mathcal {B}_J \end{array}} a_{K,L} h_K(s) k_L(t) \end{aligned}$$

is equimeasurable to $f_1'(s,t)$, and thus

4.19 $$\begin{aligned} \Vert z\Vert _Z = \left\| s\mapsto \left\| t\mapsto {{\,\mathrm{\mathbb {E}}\,}}'{{\,\mathrm{\mathbb {E}}\,}}\left| f_2'(s,t) + g(s,t) \right| \right\| _Y \right\| _X. \end{aligned}$$

Now, applying Lemma 4.10 in *X* and *Y* to (4.19) together with Proposition 2.7 (v) yields

$$\begin{aligned} \Vert z\Vert _Z \ge \left\| s\mapsto {{\,\mathrm{\mathbb {E}}\,}}^{\mathcal {F}}_s \left\| t\mapsto {{\,\mathrm{\mathbb {E}}\,}}^{\mathcal {G}}_t {{\,\mathrm{\mathbb {E}}\,}}'{{\,\mathrm{\mathbb {E}}\,}}\left| f_2'(s,t) + g(s,t) \right| \right\| _Y \right\| _X. \end{aligned}$$

Hence, by Fubini’s theorem and Jensen’s inequality, we obtain

4.20 $$\begin{aligned} \Vert z\Vert _Z \ge \left\| s\mapsto \left\| t\mapsto {{\,\mathrm{\mathbb {E}}\,}}'{{\,\mathrm{\mathbb {E}}\,}}\left| \bigl ({{\,\mathrm{\mathbb {E}}\,}}_s^{\mathcal {F}}{{\,\mathrm{\mathbb {E}}\,}}_t^{\mathcal {G}} f_2'\bigr )(s,t) + \bigl ({{\,\mathrm{\mathbb {E}}\,}}_s^{\mathcal {F}}{{\,\mathrm{\mathbb {E}}\,}}_t^{\mathcal {G}} g\bigr )(s,t) \right| \right\| _Y \right\| _X. \end{aligned}$$

We claim the following identities are true:

- ${{\,\mathrm{\mathbb {E}}\,}}^{\mathcal {F}} h_K = \theta _K \frac{|K|}{|I|}\widetilde{h}_I$, whenever $K\in \mathcal {A}_I$ for some $I\in \mathcal {D}^N$;
- ${{\,\mathrm{\mathbb {E}}\,}}^{\mathcal {G}} k_L = \varepsilon _L \frac{|L|}{|J|}\widehat{k}_J$, whenever $L\in \mathcal {B}_J$ for some $J\in \mathcal {D}^{N}$;
- ${{\,\mathrm{\mathbb {E}}\,}}^{\mathcal {F}} h_K = 0$ if $K\notin \mathcal {A}$ as well as ${{\,\mathrm{\mathbb {E}}\,}}^{\mathcal {G}} k_L = 0$ if $L\notin \mathcal {B}$.

To see this, we note that by the faithfulness of our Haar system $(\widetilde{h}_I: I\in \mathcal {D})$

4.21 $$\begin{aligned} |A_{I_0}|^{-1} \chi _{A_{I_0}} = \sum _{I\supsetneq I_0} \widetilde{h}_I(A_{I_0}) |A_I|^{-1} \widetilde{h}_I, \qquad I_0\in \mathcal {D}_N. \end{aligned}$$

Suppose now that $K\in \mathcal {A}_I$ for some $I\in \mathcal {D}^{N-1}$. Observe that whenever $K\cap A_{I_0}\ne \emptyset$ for some $I_0\in \mathcal {D}_N$, then necessarily $I_0\subset I$. This observation together with the identity (4.21) and the fact that $|A_I| = |I|$ and (4.13) yield

$$\begin{aligned} {{\,\mathrm{\mathbb {E}}\,}}^{\mathcal {F}} h_K&= \sum _{\begin{array}{c} I_0\in \mathcal {D}_{N}\\ I_0\subset I \end{array}} \langle \chi _{A_{I_0}}, h_K \rangle |A_{I_0}|^{-1} \chi _{A_{I_0}} = \sum _{\begin{array}{c} I_0\in \mathcal {D}_{N}\\ I_0\subset I \end{array}} \widetilde{h}_I(A_{I_0}) \theta _K \frac{|K|}{|I|} \chi _{A_{I_0}}\\&= \theta _K \frac{|K|}{|I|} \sum _{\begin{array}{c} I_0\in \mathcal {D}_{N}\\ I_0\subset I \end{array}} \widetilde{h}_I \chi _{A_{I_0}} = \theta _K \frac{|K|}{|I|} \widetilde{h}_I, \end{aligned}$$

as claimed in (a). For the final claim (c), suppose that $K\notin \mathcal {A}$ and observe that (4.21) and (4.13) yield immediately $\langle \chi _{A_{I_0}}, h_K \rangle = 0$ for all $I_0\in \mathcal {D}_N$, and hence ${{\,\mathrm{\mathbb {E}}\,}}^{\mathcal {F}} h_K = 0$. The proofs for (b) and the second part of (c) and the are completely analogous and therefore omitted.

Combining (a)–(c) with (4.17) and (4.18) yields

4.22 $$\begin{aligned} \begin{aligned} \bigl ({{\,\mathrm{\mathbb {E}}\,}}_s^{\mathcal {F}}{{\,\mathrm{\mathbb {E}}\,}}_t^{\mathcal {G}} f_2'\bigr )(s,t)&= \sum _{I,J\in \mathcal {D}^{N-1}} \sigma _I'^{(1)} \sigma _J'^{(2)} \sum _{\begin{array}{c} K\in \mathcal {A}_{I}\\ L\in \mathcal {B}_J \end{array}} a_{K,L} \bigl ({{\,\mathrm{\mathbb {E}}\,}}_s^{\mathcal {F}}h_K\bigr )(s) \bigl ({{\,\mathrm{\mathbb {E}}\,}}_t^{\mathcal {G}}k_L\bigr )(t)\\&= \sum _{I,J\in \mathcal {D}^{N-1}} \sigma _I'^{(1)} \sigma _J'^{(2)} \sum _{\begin{array}{c} K\in \mathcal {A}_{I}\\ L\in \mathcal {B}_J \end{array}} a_{K,L} \frac{|K| |L|}{|I| |J|} \theta _K \varepsilon _L \widetilde{h}_I(s) \widehat{k}_J(t), \end{aligned} \end{aligned}$$

as well as

4.23 $$\begin{aligned} \bigl ({{\,\mathrm{\mathbb {E}}\,}}_s^{\mathcal {F}}{{\,\mathrm{\mathbb {E}}\,}}_t^{\mathcal {G}} g\bigr )(s,t) = 0. \end{aligned}$$

We insert (4.22) and (4.23) into (4.20) and obtain

$$\begin{aligned} \Vert z\Vert _Z&\ge \left\| s\mapsto \left\| t\mapsto {{\,\mathrm{\mathbb {E}}\,}}' \left| \sum _{I_0,J_0\in \mathcal {D}_{N-1}} \sum _{\begin{array}{c} I\supset I_0\\ J\supset J_0 \end{array}} \sigma _I'^{(1)} \sigma _J'^{(2)} \sum _{\begin{array}{c} K\in \mathcal {A}_I\\ L\in \mathcal {B}_J \end{array}} a_{K,L} \frac{|K| |L|}{|I| |J|} \theta _K \varepsilon _L \widetilde{h}_I(s) \widehat{k}_J(t) \right| \right\| _Y \right\| _X\\&\ge \left\| s\mapsto \left\| t\mapsto {{\,\mathrm{\mathbb {E}}\,}}' \left| \sum _{I_0,J_0\in \mathcal {D}_{N-1}} \sum _{\begin{array}{c} I\supset I_0\\ J\supset J_0 \end{array}} \sigma _I'^{(1)} \sigma _J'^{(2)} \frac{\langle \widetilde{h}_I\otimes \widehat{h}_{J}, z\rangle }{|I| |J|} \widetilde{h}_I(s) \widehat{k}_J(t) \right| \right\| _Y \right\| _X. \end{aligned}$$

Repeating the argument below (4.15), we can exchange $\widetilde{h}_I$ by $h_I$ and $\widehat{k}_J$ by $k_J$, and thus

$$\begin{aligned} \Vert z\Vert _Z \ge \left\| s\mapsto \left\| t\mapsto {{\,\mathrm{\mathbb {E}}\,}}' \left| \sum _{I_0,J_0\in \mathcal {D}_{N-1}} \sum _{\begin{array}{c} I\supset I_0\\ J\supset J_0 \end{array}} \sigma _I'^{(1)} \sigma _J'^{(2)} \frac{\langle \widetilde{h}_I\otimes \widetilde{k}_J, z \rangle }{|I| |J|} h_I(s) k_J(t) \right| \right\| _Y \right\| _X = \Vert Qz\Vert _Z. \end{aligned}$$

By the faithfulness of our Haar systems, it is clear that $I_Z = AB$. $\square$

## Haar multipliers, pointwise multipliers and Capon’s projection

In this section we prove the required proximity results between Haar multipliers and pointwise multipliers that were stated in Sect. 2.4, which yield that Haar system Hardy spaces are 1-Capon spaces (see Definition 2.30).

### Theorem 5.1

Let $Z = Z(\varvec{\sigma },X,Y)$ be a Haar system Hardy space and let $\mathcal {V}_Z$ denote the space $\mathcal {V}(\delta ^2)$ equipped with the norm of *Z*, and put $\mathcal {V}_{Z^{\Omega }} = \mathcal {O}(\mathcal {V}_Z)$. Suppose that the Haar multiplier $D:\mathcal {V}_Z\rightarrow \mathcal {V}_Z$ given by $D h_I\otimes k_J = d_{I,J} h_I\otimes k_J$, $I,J\in \mathcal {D}$ has a finite bi-tree variation semi-norm, i.e., $\Vert D\Vert _{\mathrm {T^2}\textrm{S}} < \infty$.

Then the following estimates are true:

5.1a $$\begin{aligned} \Vert (\mathcal {O}D - M_1^D\mathcal {O})\mathcal {C}:\mathcal {V}_Z\rightarrow Z^\Omega \Vert&\le 4 \Vert D\Vert _{\mathrm {T^2}\textrm{S}},\end{aligned}$$

5.1b $$\begin{aligned} \Vert (\mathcal {O}D - M_2^D\mathcal {O})(I_Z - \mathcal {C}):\mathcal {V}_Z\rightarrow Z^\Omega \Vert&\le 4 \Vert D\Vert _{\mathrm {T^2}\textrm{S}},\end{aligned}$$

5.1c $$\begin{aligned} \Vert (M_1^D- M_2^D)\mathcal {O}\mathcal {C}:\mathcal {V}_Z \rightarrow Z^\Omega \Vert&\le \Vert D\Vert + \Vert D\Vert _{\infty } + 8\Vert D\Vert _{\mathrm {T^2}\textrm{S}}. \end{aligned}$$

### Proof

To this end, let $z = \sum _{I,J\in \mathcal {D}} a_{I,J} h_I\otimes k_J\in \mathcal {V}_Z$ with $a_{I,J}\in \mathbb {R}$ and let $s,t\in [0,1)$ be fixed.$\square$

**Estimate for **$\Vert (\mathcal {O}D - M_1^D\mathcal {O})\mathcal {C}:\mathcal {V}_Z\rightarrow Z^\Omega \Vert$. Observe that for fixed $s,t\in [0,1)$ and $\sigma \in \varvec{\sigma }$, we have

$$\begin{aligned} \bigl |\bigl ((\mathcal {O}D - M_1^D\mathcal {O})\mathcal {C}z\bigr )(s,t,\sigma )\bigr |&= \left| \sum _{j=0}^{\infty } \sum _{i=j}^{\infty } \sum _{\begin{array}{c} I\in \mathcal {D}_{i}\\ J\in \mathcal {D}_j \end{array}} \bigl (d_{I,J} - m_1^D(s,t)\bigr ) \sigma _I^{(1)} \sigma _J^{(2)} a_{I,J} h_I(s) k_J(t) \right| \\&\le \left| \sum _{j=0}^{\infty } \sum _{i=j}^{\infty } \bigl (d_{I_j(s),J_j(t)} - m_1^D(s,t)\bigr ) \sigma _{I_i(s)}^{(1)} \sigma _{J_j(t)}^{(2)} a_{I_i(s),J_j(t)} h_{I_i(s)}(s) k_{J_j(t)}(t) \right| \\&\quad + \left| \sum _{j=0}^{\infty } \sum _{i=j}^{\infty } \bigl (d_{I_i(s),J_j(t)} - d_{I_j(s),J_j(t)}\bigr ) \sigma _{I_i(s)}^{(1)} \sigma _{J_j(t)}^{(2)} a_{I_i(s),J_j(t)} h_{I_i(s)}(s) k_{J_j(t)}(t) \right| \\ {}&\qquad =: A_1 + B_1. \end{aligned}$$

We will now estimate $A_1$ and $B_1$, separately. First, note that by the definitions of $I_i(s)$, $J_j(t)$, $m_1^D$ as well as Remark 4.6 and Definition 2.11, we obtain

$$\begin{aligned} A_1&= \left| \sum _{j=0}^{\infty } \sum _{i=j}^{\infty } \sum _{k=j}^{\infty } \bigl (d_{I_k(s),J_k(t)} - d_{I_{k+1}(s),J_{k+1}(t)}\bigr ) \sigma _{I_i(s)}^{(1)} \sigma _{J_j(t)}^{(2)} a_{I_i(s),J_j(t)} h_{I_i(s)}(s) k_{J_j(t)}(t) \right| \\&\le \sum _{k=0}^{\infty } \sum _{j=0}^k \bigl |d_{I_k(s),J_k(t)} - d_{I_{k+1}(s),J_{k+1}(t)}\bigr | \cdot \left| \sum _{i=j}^{\infty } \sigma _{I_i(s)}^{(1)} \sigma _{J_j(t)}^{(2)} a_{I_i(s),J_j(t)} h_{I_i(s)}(s) k_{J_j(t)}(t) \right| \\&= \sum _{k=0}^{\infty } \sum _{j=0}^k \bigl |d_{I_k(s),J_k(t)} - d_{I_{k+1}(s),J_{k+1}(t)}\bigr | \cdot \bigl | \bigl (\mathcal {O} (I_{Z^{\Omega }} - {{\,\mathrm{\mathbb {E}}\,}}_j)\otimes ({{\,\mathrm{\mathbb {E}}\,}}_{j+1} - {{\,\mathrm{\mathbb {E}}\,}}_j) z\bigr ) (s,t)\bigr |. \end{aligned}$$

Similarly, using Remark 4.6 and Definition 2.11 yields

$$\begin{aligned} B_1&= \left| \sum _{j=0}^{\infty } \sum _{i=j}^{\infty } \sum _{k=j}^{i-1} \bigl (d_{I_{k+1}(s),J_j(t)} - d_{I_k(s),J_j(t)}\bigr ) \sigma _{I_i(s)}^{(1)} \sigma _{J_j(t)}^{(2)} a_{I_i(s),J_j(t)} h_{I_i(s)}(s) k_{J_j(t)}(t) \right| \\&\le \sum _{k=0}^{\infty } \sum _{j=0}^k \bigl |d_{I_{k+1}(s),J_j(t)} - d_{I_k(s),J_j(t)}\bigr | \cdot \left| \sum _{i=j\vee k+1}^{\infty } \sigma _{I_i(s)}^{(1)} \sigma _{J_j(t)}^{(2)} a_{I_i(s),J_j(t)} h_{I_i(s)}(s) k_{J_j(t)}(t) \right| \\&= \sum _{k=0}^{\infty } \sum _{j=0}^k \bigl |d_{I_{k+1}(s),J_j(t)} - d_{I_k(s),J_j(t)}\bigr | \cdot \bigl | \bigl (\mathcal {O} (I_{Z^{\Omega }} - {{\,\mathrm{\mathbb {E}}\,}}_{j\vee k+1})\otimes ({{\,\mathrm{\mathbb {E}}\,}}_{j+1} - {{\,\mathrm{\mathbb {E}}\,}}_j) z\bigr ) (s,t)\bigr |. \end{aligned}$$

By the monotonicity of the norm in $Z^\Omega$ and Proposition 4.5 we obtain

$$\begin{aligned}&\Vert (\mathcal {O}D - M_1^D\mathcal {O})\mathcal {C} z\Vert _{Z^{\Omega }} \le \Vert A_1\Vert _{Z^{\Omega }} + \Vert B_1\Vert _{Z^{\Omega }}\\&\quad \le \sum _{k=0}^{\infty } (k+1) \max _{\begin{array}{c} I,J\in \mathcal {D}_k\\ \omega ,\xi \in \{\pm 1\} \end{array}} |d_{I,J} - d_{I^\omega ,J^\xi }| \cdot 4 \Vert z\Vert _Z + \sum _{k=0}^{\infty } \sum _{j=0}^k \max _{\begin{array}{c} I\in \mathcal {D}_k\\ J\in \mathcal {D}_j\\ \omega \in \{\pm 1\} \end{array}} |d_{I^{\omega },J} - d_{I,J}| \cdot 4 \Vert z\Vert _Z\\&\quad \le 4 \Vert D\Vert _{\mathrm {T^2}\textrm{S}}\cdot \Vert z\Vert _{Z}, \end{aligned}$$

which establishes the first inequality in (5.1).

**Estimate for **$\Vert (\mathcal {O}D - M_2^D\mathcal {O})(I_Z - \mathcal {C}):\mathcal {V}_Z\rightarrow Z^\Omega \Vert$. For fixed $s,t\in [0,1)$ and $\sigma \in \varvec{\sigma }$, we obtain

$$\begin{aligned} \bigl |\bigl ((\mathcal {O}D&- M_1^D\mathcal {O})\mathcal {C}z\bigr )(s,t,\sigma )\bigr | = \left| \sum _{i=0}^{\infty } \sum _{j=i+1}^{\infty } \sum _{\begin{array}{c} I\in \mathcal {D}_{i}\\ J\in \mathcal {D}_j \end{array}} \bigl (d_{I,J} - m_2^D(s,t)\bigr ) \sigma _I^{(1)} \sigma _J^{(2)} a_{I,J} h_I(s) k_J(t) \right| \\&\le \left| \sum _{i=0}^{\infty } \sum _{j=i+1}^{\infty } \bigl (d_{I_i(s),J_{i+1}(t)} - m_2^D(s,t)\bigr ) \sigma _{I_i(s)}^{(1)} \sigma _{J_j(t)}^{(2)} a_{I_i(s),J_j(t)} h_{I_i(s)}(s) k_{J_j(t)}(t) \right| \\&\qquad + \left| \sum _{i=0}^{\infty } \sum _{j=i+1}^{\infty } \bigl (d_{I_i(s),J_j(t)} - d_{I_i(s),J_{i+1}(t)}\bigr ) \sigma _{I_i(s)}^{(1)} \sigma _{J_j(t)}^{(2)} a_{I_i(s),J_j(t)} h_{I_i(s)}(s) k_{J_j(t)}(t) \right| \\&=: A_2 + B_2. \end{aligned}$$

By the definitions of $I_i(s)$, $J_j(t)$, $m_2^D$ as well as Remark 4.6 and Definition 2.11, we obtain

$$\begin{aligned} A_2&= \left| \sum _{i=0}^{\infty } \sum _{j=i+1}^{\infty } \sum _{k=i}^{\infty } \bigl (d_{I_k(s),J_{k+1}(t)} - d_{I_{k+1}(s),J_{k+2}(t)}\bigr ) \sigma _{I_i(s)}^{(1)} \sigma _{J_j(t)}^{(2)} a_{I_i(s),J_j(t)} h_{I_i(s)}(s) k_{J_j(t)}(t) \right| \\&\le \sum _{k=0}^{\infty } \sum _{i=0}^k |d_{I_k(s),J_{k+1}(t)} - d_{I_{k+1}(s),J_{k+2}(t)}| \cdot \left| \sum _{j=i+1}^{\infty } \sigma _{I_i(s)}^{(1)} \sigma _{J_j(t)}^{(2)} a_{I_i(s),J_j(t)} h_{I_i(s)}(s) k_{J_j(t)}(t) \right| \\&= \sum _{k=0}^{\infty } \sum _{i=0}^k |d_{I_k(s),J_{k+1}(t)} - d_{I_{k+1}(s),J_{k+2}(t)}| \cdot \bigl | \bigl (\mathcal {O} ({{\,\mathrm{\mathbb {E}}\,}}_{i+1} - {{\,\mathrm{\mathbb {E}}\,}}_i)\otimes (I_{Z^{\Omega }} - {{\,\mathrm{\mathbb {E}}\,}}_{i+1}) z\bigr ) (s,t)\bigr |. \end{aligned}$$

Similarly, using Remark 4.6 and Definition 2.11 yields

$$\begin{aligned} B_2&= \left| \sum _{i=0}^{\infty } \sum _{j=i+1}^{\infty } \sum _{k=i+1}^{j-1} \bigl (d_{I_i(s),J_{k+1}(t)} - d_{I_i(s),J_k(t)}\bigr ) \sigma _{I_i(s)}^{(1)} \sigma _{J_j(t)}^{(2)} a_{I_i(s),J_j(t)} h_{I_i(s)}(s) k_{J_j(t)}(t) \right| \\&\le \sum _{k=1}^{\infty } \sum _{i=0}^{k-1} |d_{I_i(s),J_{k+1}(t)} - d_{I_i(s),J_k(t)}| \cdot \left| \sum _{j=k+1}^{\infty } \sigma _{I_i(s)}^{(1)} \sigma _{J_j(t)}^{(2)} a_{I_i(s),J_j(t)} h_{I_i(s)}(s) k_{J_j(t)}(t) \right| \\&= \sum _{k=1}^{\infty } \sum _{i=0}^{k-1} |d_{I_i(s),J_{k+1}(t)} - d_{I_i(s),J_k(t)}| \cdot \bigl | \bigl (\mathcal {O} ({{\,\mathrm{\mathbb {E}}\,}}_{i+1} - {{\,\mathrm{\mathbb {E}}\,}}_i)\otimes (I_{Z^{\Omega }} - {{\,\mathrm{\mathbb {E}}\,}}_{k+1}) z\bigr ) (s,t)\bigr |. \end{aligned}$$

By the monotonicity of the norm in $Z^\Omega$, and Proposition 4.5 we obtain

$$\begin{aligned}&\Vert (\mathcal {O}D - M_2^D\mathcal {O})(I_Z - \mathcal {C}) z\Vert _{Z^\Omega } \le \Vert A_2\Vert _{Z^{\Omega }} + \Vert B_2\Vert _{Z^{\Omega }}\\&\quad \le \sum _{k=0}^{\infty } (k+1) \max _{\begin{array}{c} I\in \mathcal {D}_k\\ J\in \mathcal {D}_{k+1}\\ \omega ,\xi \in \{\pm 1\} \end{array}} |d_{I,J} - d_{I^\omega ,J^\xi }| \cdot 4 \Vert z\Vert _Z + \sum _{k=1}^{\infty } \sum _{i=0}^{k-1} \max _{\begin{array}{c} I\in \mathcal {D}_i\\ J\in \mathcal {D}_k\\ \xi \in \{\pm 1\} \end{array}} |d_{I,J^{\xi }} - d_{I,J}| \cdot 4 \Vert z\Vert _Z\\&\quad \le 4 \Vert D\Vert _{\mathrm {T^2}\textrm{S}}\cdot \Vert z\Vert _{Z}, \end{aligned}$$

which establishes the second estimate in (5.1).

**Estimate for **$\Vert (M_2^D - M_1^D)\mathcal {O}\mathcal {C}:\mathcal {V}_Z\rightarrow Z^\Omega \Vert$. Observe that by (5.1), and by the definition of $M_2^D$, we have $\Vert M_2^D\Vert \le \Vert D\Vert$, and thus

$$\begin{aligned} \Vert D z\Vert _Z&= \Vert \mathcal {O} D z\Vert _{Z^{\Omega }} = \Vert \mathcal {O} D \mathcal {C} z + \mathcal {O} D (I_Z-\mathcal {C})\Vert _ z{Z^{\Omega }}\\&\ge \Vert M_1^D \mathcal {O} \mathcal {C} z + M_2^D\mathcal {O} (I_Z-\mathcal {C}) z\Vert _{Z^{\Omega }} - 8\Vert D\Vert _{\mathrm {T^2}\textrm{S}}\cdot \Vert z\Vert _Z\\&= \Vert (M_1^D - M_2^D) \mathcal {O} \mathcal {C} z + M_2^D\mathcal {O} z\Vert _{Z^{\Omega }} - 8\Vert D\Vert _{\mathrm {T^2}\textrm{S}}\cdot \Vert z\Vert _Z\\&\ge \Vert (M_1^D - M_2^D) \mathcal {O} \mathcal {C} z\Vert _{Z^{\Omega }} - \Vert M_2^D\mathcal {O} z\Vert _{Z^{\Omega }} - 8\Vert D\Vert _{\mathrm {T^2}\textrm{S}}\cdot \Vert z\Vert _Z\\&\ge \Vert (M_1^D - M_2^D) \mathcal {O} \mathcal {C} z\Vert _{Z^{\Omega }} - \Vert D\Vert _{\infty }\cdot \Vert z\Vert _Z - 8\Vert D\Vert _{\mathrm {T^2}\textrm{S}}\cdot \Vert z\Vert _Z, \end{aligned}$$

as claimed. $$\square $$

### Corollary 5.2

Assuming the conditions of Theorem 5.1 and assuming that

$$\begin{aligned} \Vert D\Vert _{{\mathrm {T^2}\textrm{S}}} < |\lambda _\mathcal {U} (D)-\mu _\mathcal {U}(D)|/8 \end{aligned}$$

it follows that

$$\begin{aligned} \Vert \mathcal {C}\Vert _{\mathcal {L}(Z)} \le \frac{\Vert D\Vert +\Vert D\Vert _\infty + 8 \Vert D\Vert _{{\mathrm {T^2}\textrm{S}}}}{|\lambda _\mathcal {U} (D) - \mu _\mathcal {U}(D)| - 8\Vert D\Vert _{{\mathrm {T^2}\textrm{S}}}}. \end{aligned}$$

and thus, $\mathcal {C}:Z\rightarrow Z$ is bounded if $\Vert D:Z\rightarrow Z\Vert < \infty$.

In particular, $\mathcal {C}$ is bounded on *Z* if and only if there exists a bounded Haar multiplier $D:Z\rightarrow Z$ such that $\lambda _\mathcal {U}(D)\ne \mu _\mathcal {U}(D)$.

### Proof

Put $\lambda = \lambda _\mathcal {U}(D)$ and $\mu = \mu _\mathcal {U}(D)$ note that

$$\begin{aligned} \Vert (\lambda - \mu ) \mathcal {C} z\Vert _Z&= \Vert (\lambda I_{Z^{\Omega }} - M_1^D) \mathcal {O} \mathcal {C} z - (\mu I_{Z^{\Omega }} - M_2^D) \mathcal {O} \mathcal {C} z + (M_1^D - M_2^D)\mathcal {O} \mathcal {C} z\Vert _{Z^{\Omega }}\\&\le \Vert (\lambda I_{Z^{\Omega }} - M_1^D) \mathcal {O} \mathcal {C} z\Vert _{Z^{\Omega }} + \Vert (\mu I_{Z^{\Omega }} - M_2^D) \mathcal {O} \mathcal {C} z\Vert _{Z^{\Omega }}\\&+ \Vert (M_1^D - M_2^D)\mathcal {O} \mathcal {C} z\Vert _{Z^{\Omega }}\\&\le 8\Vert D\Vert _{\mathrm {T^2}\textrm{S}}\cdot \Vert \mathcal {C} z\Vert _Z + (\Vert D\Vert + \Vert D\Vert _{\infty } + 8\Vert D\Vert _{\mathrm {T^2}\textrm{S}})\cdot \Vert z\Vert _{Z^{\Omega }}. \end{aligned}$$

Hence, if $8\Vert D\Vert _{\mathrm {T^2}\textrm{S}} < |\lambda - \mu |$, then

$$\begin{aligned} \Vert \mathcal {C} z\Vert _Z \le \frac{\Vert D\Vert + \Vert D\Vert _{\infty } + 8\Vert D\Vert _{\mathrm {T^2}\textrm{S}}}{|\lambda - \mu | - 8\Vert D\Vert _{\mathrm {T^2}\textrm{S}}}\cdot \Vert z\Vert _{Z^{\Omega }}. \end{aligned}$$

For the final part, assume that there exists a bounded Haar multiplier $D:Z\rightarrow Z$ such that $\lambda _\mathcal {U}(D)\ne \mu _\mathcal {U}(D)$. By Theorems 3.20 and 4.9, we may pass to a bounded Haar multiplier $\tilde{D}:Z\rightarrow Z$ such that $\Vert \tilde{D}\Vert _{\mathrm {T^2}\textrm{S}} < |\lambda _\mathcal {U}(D) - \mu _\mathcal {U}(D)|/8$ and $\lambda _\mathcal {U}(\tilde{D}) = \lambda _\mathcal {U}(D)$, $\mu _\mathcal {U}(\tilde{D}) = \mu _\mathcal {U}(D)$, which yields the conclusion.$\square$

### Theorem 5.3

Let $Z = Z(\mathbf {\sigma },X,Y)\in \mathcal{H}\mathcal{H}(\delta ^2)$ and $\mathcal {V}_Z = \mathcal {V}(\delta ^2)$ with the norm induced by *Z*. Suppose that $m:[0,1)^2\rightarrow \mathbb {R}$ is continuous almost everywhere with $\Vert m\Vert _{L^\infty ([0,1)^2)}\le 1$, and let the pointwise multiplication operator $M:\mathcal {V}_Z\rightarrow Z^\Omega$ be given by

$$\begin{aligned} (Mw)(s,t,\sigma ) = m(s,t)\cdot w(s,t,\sigma ), \qquad s,t\in [0,1),\ \sigma \in \varvec{\sigma }. \end{aligned}$$

If $\mathcal {C}:Z\rightarrow Z$ is unbounded but $M\mathcal {O}\mathcal {C}:\mathcal {V}_Z \rightarrow Z^\Omega$ is bounded, then $M=0$.

### Proof

Suppose that $|\{ |m|> 0\}| > 0$, then there exists $k_0\in \mathbb {N}$ such that $|\{ |m|> 1/k_0\}| > 0$. By the almost everywhere continuity of *m*, there exist dyadic intervals $I_0,J_0\in \mathcal {D}$ with $|I_0| = |J_0| = 2^{-l_0}$ such that $I_0\times J_0{\setminus } N\subset \{ |m| > 1/k_0\}$, where $|N| = 0$. Define the pointwise multiplier $R_{I_0, J_0}:Z^\Omega \rightarrow Z^\Omega$ by $(R_{I_0, J_0} w)(s,t,\sigma ) = \chi _{I_0\times J_0}(s,t) w(s,t,\sigma )$ and let $z\in \mathcal {V}_Z$. By the monotonicity of the norm in $Z^\Omega$, we obtain

5.2 $$\begin{aligned} \begin{aligned} \Vert M\mathcal {O}\mathcal {C}z\Vert _{Z^\Omega }&\ge \Vert R_{I_0, J_0} M\mathcal {O}\mathcal {C}z\Vert _{Z^\Omega }\\&= \left\| s\mapsto \left\| t\mapsto {{\,\mathrm{\mathbb {E}}\,}}\bigl | \chi _{I_0\times J_0}(s,t) m(s,t) (\mathcal {O}\mathcal {C}z)(s,t,\sigma ) \bigr | \right\| _Y \right\| _X\\&\ge \frac{1}{k_0} \left\| s\mapsto \left\| t\mapsto {{\,\mathrm{\mathbb {E}}\,}}\bigl | \chi _{I_0\times J_0}(s,t) (\mathcal {O}\mathcal {C} w)(s,t,\sigma ) \bigr | \right\| _Y \right\| _X\\&= \frac{1}{k_0} \Vert R_{I_0, J_0} \mathcal {O}\mathcal {C} z\Vert _{Z^\Omega }. \end{aligned} \end{aligned}$$

In the following, we will use the identity

5.3 $$\begin{aligned} I_{Z} = (I_X - {{\,\mathrm{\mathbb {E}}\,}}_{l_0})\otimes (I_Y - {{\,\mathrm{\mathbb {E}}\,}}_{l_0}) + (I_X - {{\,\mathrm{\mathbb {E}}\,}}_{l_0})\otimes {{\,\mathrm{\mathbb {E}}\,}}_{l_0} + {{\,\mathrm{\mathbb {E}}\,}}_{l_0}\otimes (I_Y - {{\,\mathrm{\mathbb {E}}\,}}_{l_0}) + {{\,\mathrm{\mathbb {E}}\,}}_{l_0}\otimes {{\,\mathrm{\mathbb {E}}\,}}_{l_0} \end{aligned}$$

to split $R_{I_0, J_0} \mathcal {O}\mathcal {C}z$ into parts and estimate them separately. Let $z = \sum _{I,J} a_{I,J} h_I\otimes k_J\in \mathcal {V}_Z$ with $a_{I,J}\in \mathbb {R}$.

First, observe that by the definition of ${{\,\mathrm{\mathbb {E}}\,}}_{l_0}$ and $\mathcal {C}$

5.4 $$\begin{aligned} R_{I_0, J_0}\mathcal {O}(I_X - {{\,\mathrm{\mathbb {E}}\,}}_{l_0})\otimes {{\,\mathrm{\mathbb {E}}\,}}_{l_0}\mathcal {C}z = 0. \end{aligned}$$

Secondly, the definitions of $R_{I_0, J_0}$, $S_{I_0, J_0}$, ${{\,\mathrm{\mathbb {E}}\,}}_{l_0}$ and the monotonicity of the norm in $Z^\Omega$ yield

$$\begin{aligned} \Vert R_{I_0, J_0}\mathcal {O}({{\,\mathrm{\mathbb {E}}\,}}_{l_0}\otimes (I_Y - {{\,\mathrm{\mathbb {E}}\,}}_{l_0})) \mathcal {C}z\Vert _{Z^\Omega }&= \left\| \sum _{\begin{array}{c} |I|> 2^{-l_0}\\ |J|\le 2^{-l_0} \end{array}} a_{I,J} ((\chi _{I_0}h_I)\otimes (\chi _{J_0}k_J))\otimes (\sigma _I^{(1)}\otimes \sigma _J^{(2)}) \right\| _{Z^\Omega }\\&\le \sum _{|I|> 2^{-l_0}} \left\| \sum _{J\subset J_0} a_{I,J} ((\chi _{I_0} h_I)\otimes k_J)\otimes (\sigma _I^{(1)}\otimes \sigma _J^{(2)}) \right\| _{Z^\Omega }\\&\le \sum _{|I|> 2^{-l_0}} \left\| \sum _{J\subset J_0} a_{I,J} (h_I\otimes k_J)\otimes (\sigma _I^{(1)}\otimes \sigma _J^{(2)}) \right\| _{Z^\Omega }\\&= \sum _{|I|> 2^{-l_0}} \left\| \sum _{J\subset J_0} a_{I,J} h_I\otimes k_J \right\| _{Z}\\&= \sum _{|I| > 2^{-l_0}} \bigl \Vert S_{I, J_0}(p_I\otimes I_Y)z \bigr \Vert _{Z}. \end{aligned}$$

By Propositions 4.5 and 4.7, we obtain

5.5 $$\begin{aligned} \Vert R_{I_0, J_0}\mathcal {O}{{\,\mathrm{\mathbb {E}}\,}}_{l_0}\otimes (I_Y - {{\,\mathrm{\mathbb {E}}\,}}_{l_0}) \mathcal {C}z\Vert _{Z^\Omega } \le \sum _{|I| > 2^{-l_0}} 8 \Vert z\Vert _{Z} \le 2^{l_0+3} \Vert z\Vert _{Z}. \end{aligned}$$

Thirdly, note that by Proposition 4.5

$$\begin{aligned} 4\Vert z\Vert _{Z}&\ge \Vert p_I\otimes p_Jz\Vert _{Z} = \left\| a_{I,J} (h_I\otimes k_J)\otimes (\sigma _I^{(1)}\otimes \sigma _J^{(2)})\right\| _{Z^\Omega }, \end{aligned}$$

hence, by the above inequality, we obtain

$$\begin{aligned} \Vert R_{I_0, J_0}\mathcal {O}({{\,\mathrm{\mathbb {E}}\,}}_{l_0}\otimes {{\,\mathrm{\mathbb {E}}\,}}_{l_0}) \mathcal {C}z\Vert _{Z^\Omega }&= \left\| \sum _{|I|,|J|> 2^{-l_0}} a_{I,J} (\chi _{I_0}h_I\otimes \chi _{J_0}k_J)\otimes (\sigma _I^{(1)}\otimes \sigma _J^{(2)}) \right\| _{Z^\Omega }\\&\le \sum _{|I|,|J|> 2^{-l_0}} \left\| a_{I,J} (h_I\otimes k_J)\otimes (\sigma _I^{(1)}\otimes \sigma _J^{(2)}) \right\| _{Z^\Omega }\\&\le \sum _{|I|,|J| > 2^{-l_0}} 4 \Vert z\Vert _{Z^\Omega }. \end{aligned}$$

We record the estimate

5.6 $$\begin{aligned} \Vert R_{I_0, J_0}\mathcal {O}{{\,\mathrm{\mathbb {E}}\,}}_{l_0}\otimes {{\,\mathrm{\mathbb {E}}\,}}_{l_0} \mathcal {C} z\Vert _{Z^\Omega } \le 4^{l_0+1} \Vert z\Vert _{Z}. \end{aligned}$$

Combining (5.2), (5.3), (5.4), (5.5) and (5.6) yields

$$\begin{aligned} k_0\cdot \Vert M\mathcal {O}\mathcal {C}z\Vert _{Z^\Omega }&\ge \Vert R_{I_0, J_0} \mathcal {O}(I_X - {{\,\mathrm{\mathbb {E}}\,}}_{l_0})\otimes I_Y - {{\,\mathrm{\mathbb {E}}\,}}_{l_0}) \mathcal {C} z \Vert _{Z^\Omega } - \Vert R_{I_0, J_0} \mathcal {O}(I_X - {{\,\mathrm{\mathbb {E}}\,}}_{l_0})\otimes {{\,\mathrm{\mathbb {E}}\,}}_{l_0} \mathcal {C} z\Vert _{Z^\Omega }\\&\quad - \Vert R_{I_0, J_0} \mathcal {O}{{\,\mathrm{\mathbb {E}}\,}}_{l_0}\otimes (I_Y - {{\,\mathrm{\mathbb {E}}\,}}_{l_0}) \mathcal {C} z\Vert _{Z^\Omega } - \Vert R_{I_0, J_0} \mathcal {O}{{\,\mathrm{\mathbb {E}}\,}}_{l_0}\otimes {{\,\mathrm{\mathbb {E}}\,}}_{l_0} \mathcal {C} z\Vert _{Z^\Omega }\\&\ge \Vert R_{I_0, J_0} \mathcal {O}(I_X - {{\,\mathrm{\mathbb {E}}\,}}_{l_0})\otimes (I_Y - {{\,\mathrm{\mathbb {E}}\,}}_{l_0}) \mathcal {C} z \Vert _{Z^\Omega } - (2^{l_0+3} + 4^{l_0+1})\Vert z\Vert _{Z}. \end{aligned}$$

Note that by definition of $R_{I_0, J_0}$, ${{\,\mathrm{\mathbb {E}}\,}}_{l_0}$ and $S_{I_0, J_0}$, we obtain

$$\begin{aligned} R_{I_0, J_0} \mathcal {O}(I_X - {{\,\mathrm{\mathbb {E}}\,}}_{l_0})\otimes (I_Y - {{\,\mathrm{\mathbb {E}}\,}}_{l_0}) \mathcal {C}z = \mathcal {O}S_{I_0, J_0} \mathcal {C}z, \end{aligned}$$

and thus, since $\mathcal {O}$ is an isometry, the latter estimate yields

5.7 $$\begin{aligned} \Vert S_{I_0, J_0} \mathcal {C}z\Vert _{Z} = \Vert \mathcal {O}S_{I_0, J_0} \mathcal {C}z\Vert _{Z^\Omega } \le (k_0\cdot \Vert M\mathcal {O}\mathcal {C}\Vert + 2^{l_0+3} + 4^{l_0+1})\cdot \Vert z\Vert _{Z}. \end{aligned}$$

On the other hand, since the Capon projection $\mathcal {C}$ commutes with both $\Delta _{I_0, J_0}$ and $S_{I_0, J_0}$, we obtain by Proposition 4.8, and (5.7) that

$$\begin{aligned} \Vert \mathcal {C} z\Vert _{Z}&= \Vert \Upsilon _{I_0, J_0}\Delta _{I_0, J_0}\mathcal {C} z\Vert _{Z} \le \Vert \Upsilon _{I_0, J_0}\Vert \cdot \Vert \mathcal {C}\Delta _{I_0, J_0} z\Vert _{Z} = \Vert \Upsilon _{I_0, J_0}\Vert \cdot \Vert \mathcal {C}S_{I_0, J_0}\Delta _{I_0, J_0} z\Vert _{Z}\\&\le \Vert \Upsilon _{I_0, J_0}\Vert \cdot \Vert S_{I_0, J_0}\mathcal {C}\Vert \cdot \Vert \Delta _{I_0, J_0} z\Vert _{Z} \le 4^{l_0}\cdot \bigl ( k_0\cdot \Vert M\mathcal {O}\mathcal {C}\Vert + (2^{l_0+3} + 4^{l_0+1}) \bigr ) \cdot \Vert z\Vert _{Z}. \end{aligned}$$

Contrary to our hypothesis, this implies the boundedness of the Capon projection $\mathcal {C}:Z\rightarrow Z$; hence, $|\{ |m| > 0\}| = 0$, i.e., $M = 0$. $\square$

## Data Availability

We do not analyze or generate any datasets, because our work proceeds within a theoretical and mathematical approach.
